# Benzothiazinone analogs as Anti-*Mycobacterium tuberculosis* DprE1 irreversible inhibitors: Covalent docking, validation, and molecular dynamics simulations

**DOI:** 10.1371/journal.pone.0314422

**Published:** 2024-11-25

**Authors:** Mahmoud A. A. Ibrahim, Doaa G. M. Mahmoud, Alaa H. M. Abdelrahman, Khlood A. A. Abdeljawaad, Gamal A. H. Mekhemer, Tamer Shoeib, Mohamed A. El-Tayeb, Peter A. Sidhom, Paul W. Paré, Mohamed-Elamir F. Hegazy

**Affiliations:** 1 Faculty of Science, Chemistry Department, Computational Chemistry Laboratory, Minia University, Minia, Egypt; 2 School of Health Sciences, University of KwaZulu-Natal, Westville Campus, Durban, South Africa; 3 Department of Chemistry, The American University in Cairo, New Cairo, Egypt; 4 Department of Botany and Microbiology, College of Science, King Saud University, Riyadh, Saudi Arabia; 5 Faculty of Pharmacy, Department of Pharmaceutical Chemistry, Tanta University, Tanta, Egypt; 6 Department of Chemistry & Biochemistry, Texas Tech University, Lubbock, TX, United States of America; 7 Department of Pharmaceutical Biology, Institute of Pharmaceutical and Biomedical Sciences, Johannes Gutenberg University, Mainz, Germany; Jhargram Raj College, INDIA

## Abstract

*Mycobacterium tuberculosis* is a lethal human pathogen, with the key flavoenzyme for catalyzing bacterial cell-wall biosynthesis, decaprenylphosphoryl-D-ribose oxidase (DprE1), considered an Achilles heal for tuberculosis (TB) progression. Inhibition of DprE1 blocks cell wall biosynthesis and is a highly promising antitubercular target. Macozinone (PBTZ169, a benzothiazinone (BTZ) derivative) is an irreversible DprE1 inhibitor that has attracted considerable attention because it exhibits an additive activity when combined with other anti-TB drugs. Herein, 754 BTZ analogs were assembled in a virtual library and evaluated against the DprE1 target using a covalent docking approach. After validation of the employed covalent docking approach, BTZ analogs were screened. Analogs with a docking score less than –9.0 kcal/mol were advanced for molecular dynamics (MD) simulations, followed by binding energy evaluations utilizing the MM-GBSA approach. Three BTZ analogs–namely, PubChem-155-924-621, PubChem-127-032-794, and PubChem-155-923-972– exhibited higher binding affinities against DprE1 compared to PBTZ169 with Δ*G*_binding_ values of –77.2, –74.3, and –65.4 kcal/mol, versus –49.8 kcal/mol, respectively. Structural and energetical analyses were performed for the identified analogs against DprE1 throughout the 100 ns MD simulations, and the results demonstrated the great stability of the identified BTZ analogs. Physicochemical and ADMET characteristics indicated the oral bioavailability of the identified BTZ analogs. The obtained *in-silico* results provide promising anti-TB inhibitors that are worth being subjected to *in-vitro* and *in-vivo* investigations.

## Introduction

*Mycobacterium tuberculosis* is a slow-growing pathogenic bacterium that requires up to six months of antibiotic treatment for eradication [1]. With the development of antibiotic resistance, it persists as a health threat, especially in low- and middle-income nations [2–4]. The resurgence of tuberculosis (TB) as a global pandemic has been influenced in part by a lack of an effective vaccine [5] as well as by a significant increase in TB drug resistance [6]. At the same time, there has been a global refocusing over the last twenty years by health organizations that have resulted in a significant reduction in TB-related mortality [7].

A principal flavoenzyme in *M*. *tuberculosis* cell-wall formation is decaprenyl-phosphoryl-D-ribose oxidase (DprE1); indeed, several potent covalent and noncovalent DprE1 inhibitors have been identified [8,9], including isoniazid, rifampin, pyrazinamide, ethambutol, and streptomycin that showed promise as anti-TB agents [10,11]. Currently, four DprE1 inhibitors have entered clinical trials–namely, BTZ043, TBA-7371, OPC-167832, and macozinone (PBTZ169). OPC-167832 and TBA-7371 are noncovalent inhibitors that function as highly effective antimycobacterial agents [12,13]. Among the covalent-specific DrpE1 inhibitors, 8-nitrobenzothiazinone (BTZ) analogs, including macozinone (PBTZ169), demonstrated promising inhibitory activity in *in-vitro* tests [14–16]. The electrophilic nitro group of BTZ is the apparent covalent warhead, which forms an irreversible covalent bond with the nucleophilic CYS387 residue in the enzyme active site [17]. According to a previous study, PBTZ169 was three to seven times more effective than BTZ043 against *M*. *tuberculosis* [18]. A previous study indicated that PBTZ169 could significantly improve patient survivorship in cases suffering from multidrug-resistant TB [19]. In fact, PBTZ169 is currently undergoing phase I/II clinical trials for the treatment of TB [20], and a PBTZ169, pyrazinamide, bedaquiline cocktail is being considered as a prospective anti-TB regime [18].

**Fig 1:**
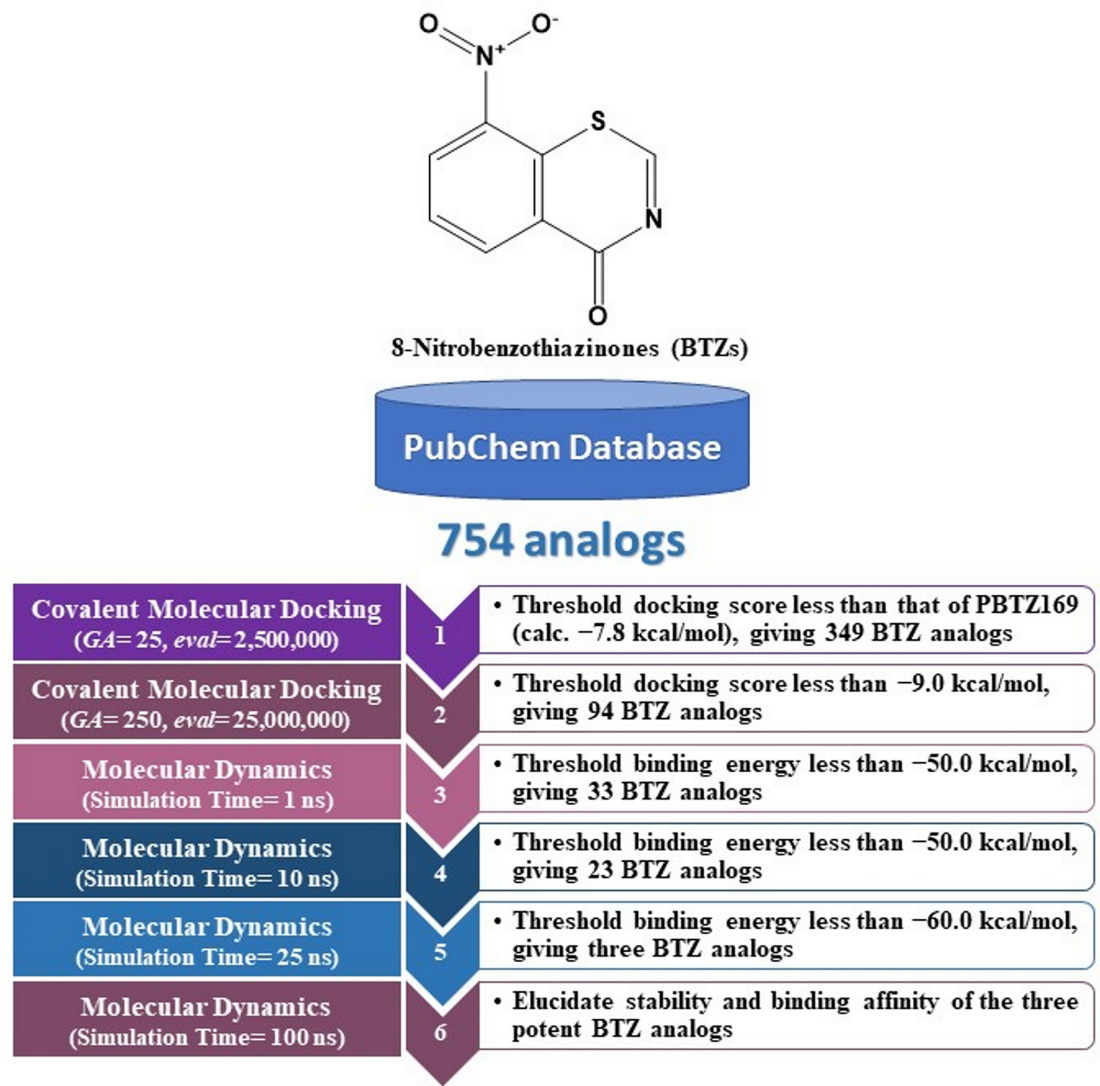
Schematic diagram of the utilized *in-silico* approaches in the virtual screening strategy of BTZ analogs.

In the current study, 754 BTZ analogs were obtained from the PubChem database and virtually screened as prospective DprE1 inhibitors using covalent docking computations. Based on the covalent docking scores, molecular dynamics (MD) simulations for potent BTZ analogs in complex with DprE1 were examined. Furthermore, post-MD analyses were inspected, and the corresponding binding energies were computed utilizing the MM-GBSA approach. Physicochemical and pharmacokinetic features of the identified BTZ analogs were also predicted. A schematic diagram of the *in-silico* methods employed for screening BTZ analogs is shown in Fig 1. Such *in-silico* computations provide insight with regard to the suitability of the identified BTZ analogs for the future development of potential anti-TB drug candidates.

## Computational methodology

### DprE1 preparation

The crystal structure of *M*. *tuberculosis* DprE1 in complex with PBTZ169 ligand (PDB code: 4NCR [18]) was utilized as a template for all covalent molecular docking and molecular dynamics computations. The non-terminal missing residues were constructed using the Modeller software [21], which are located at 272–283 and 315–330. DprE1 enzyme was prepared by eliminating all ions, inhibitor, and water molecules. The protonation states were then examined using the H++ web server [22]. Besides, all missing hydrogen atoms were added with the following parameters: external dielectric = 80, salinity = 0.15, internal dielectric = 10, and pH = 7.0. The quality of the modeled structure was evaluated based on the Ramachandran plot occupancy of residues, utilizing the PROCHECK server [23].

### Covalent inhibitors preparation

In the current study, a total of 785 BTZ analogs were retrieved from the PubChem database (https://pubchem.ncbi.nlm.nih.gov) in an SDF format. The duplicated compounds were eliminated according to the International Chemical Identifier key (InChIKey), giving 754 BTZ analogs [24]. Subsequently, the three-dimensional (3D) structures of BTZ analogs were created using Omega2 software with a maximum of 200 conformers generated within a 10 kcal/mol energy window [25,26]. The ionization state of each BTZ analog was examined using the Fixpka tool within the QUACPAC software [27]. The geometrical structures were subsequently minimized by the MMFF94S force field within SZYBKI software [28,29]. The charges of the investigated BTZ analogs were estimated using the Gasteiger-Marsili method [30].

### Covalent docking

All covalent docking calculations were conducted using AutoDock4.2.6 software [31]. The applied docking protocol keeps the macromolecule rigid while permitting flexibility in the ligand. For preparation purposes, the DprE1 enzyme was converted into a pdbqt file [32]. Except for the number of genetic algorithm (*GA*) runs and the maximum number of energy evaluations (*eval*), all covalent docking parameters were adjusted to their default settings. Fast and expensive accuracy levels of calculations were employed to virtually screen the BTZ analogs. For docking computations, the *GA* and *eval* parameters were increased from 25 and 2,500,000 to 250 and 25,000,000 for fast and expensive levels of accuracy, respectively. In both levels of calculations, the grid box was placed at 40 Å × 40 Å × 40 Å. The grid spacing value of 0.375 Å was used. The grid box was centered on 17.176, −20.119, and 1.875 (XYZ coordinates). The predicted docking modes for each BTZ analog were processed using a built-in clustering analysis with an RMSD tolerance of 1.0 Å. The lowest energy conformation from the largest cluster was chosen as the representative docking mode.

### MD simulations

Molecular dynamics (MD) simulations of the most potent BTZ analogs complexed with the DprE1 enzyme were carried out using AMBER20 software [33]. The details of the utilized MD simulations are described in Ref. [34–38]. DprE1 enzyme and BTZ analogs were characterized using the AMBER force field of 14SB and the General AMBER force field (GAFF2), respectively [39,40]. For atomic charges calculations, the irreversible covalent inhibitors with CYS387 residue were capped using acetyl and methylamide groups and subjected to geometrical optimization at B3LYP/6-31G* level of theory using Gaussian09 software [41]. A restrained electrostatic potential (RESP) approach at HF/6-31G* level was then utilized to assign the atomic partial charges of the optimized inhibitors [42]. The parameters of covalent inhibitors with CYS387 residue (included in the covalent bond exhibition) and atom types were characterized and defined utilizing an antechamber module within the AMBER package. The docked BTZ analog-DprE1 complexes were solvated in an octahedron box of TIP3P water molecules. Sodium or chloride counterions were inserted to neutralize all solvated systems. Moreover, the ionic strength of the solution was also tuned to 0.15 M NaCl. Nevertheless, all prepared DprE1-analogs complexes were minimized for 5000 cycles. The minimized systems were gently heated up to 310 K for 50 ps. The DprE1-analogs complexes were equilibrated with a simulation time of 10 ns. After that, the production stages were run on the equilibrated systems throughout 1, 10, 25, and 100 ns. The snapshots were assembled each 10 ps through all production stages. The Particle Mesh Ewald (PME) method was employed to handle long-range electrostatic interactions under periodic boundary conditions, with a cutoff of 12 Å [43]. To maintain the temperature at 298 K, Langevin dynamics were used with a collision frequency (gamma_ln) set to 1.0. Pressure control was achieved using a Berendsen barostat with a relaxation time of 2 ps [44]. Bonds involving hydrogen atoms were constrained using the SHAKE algorithm, with a time step of 2 fs [45]. All calculations, including MD simulations, quantum mechanics computations, and covalent molecular docking estimations, were undertaken using the CompChem GPU (pmemd.cuda)/CPU hybrid cluster (hpc.compchem.net). All graphical representations were generated with the assistance of BIOVIA Discovery Studio Visualizer [46].

### Binding energy computations

The binding energy of the most promising BTZ analogs in complex with DprE1 enzyme was computed using the MM-GBSA (molecular mechanics-generalized Born surface area) approach [47]. The modified GB model proposed by Onufriev *et al*. (igb = 2) was used to calculate the polar solvation energy [48]. The following equation can be used to compute the MM-GBSA binding energy:

$${\Delta G}_{binding}=G_{Complex}-{(G}_{DprE1}{+G}_{BTZ\;analog})$$ Eq (1)

where energy term (*G*) is given as follows:

$$G=G_{solve}+E_{MM}-TS$$ Eq (2)

$$E_{MM}=E_{vdW}+E_{int}+E_{ele}$$ Eq (3)

$$E_{int}=E_{angle}+E_{bond}+E_{torsion}$$ Eq (4)

*E*_MM_ stands for molecular mechanics gas-phase energy. *G*_solv_ indicates the solvation energy. *E*_int_ is the internal MM energy involving angle, bond, and dihedral energies. *E*_vdW_ indicates van der Waals energy. *E*_ele_ is electrostatic energy. Entropy contributions were disregarded owing to the high computational cost [49,50].

### Physicochemical features

The online SWISS-ADME (https://www.swissadme.ch) server was employed to investigate the physicochemical features of the most potent BTZ analogs as DprE1 irreversible inhibitors. These physicochemical characteristics included MW (molecular weight), HBD (number of H-bond donors), TPSA (topological polar surface area), HBA (number of H-bond acceptors), and Mlog*P* (n-octanol/water partition coefficient).

### Anticipation of the pharmacokinetic and toxicity characteristics

The online pkCSM server was used to estimate the pharmacokinetic features (http://biosig.unimelb.edu.au/pkcsm/prediction). Pharmacokinetic characteristics involve absorption, distribution, metabolism, and excretion. Besides, the toxicity feature was predicted. Absorption (A) is evaluated based on water solubility (log*S*). The distribution (D) was assessed based on Log BB (blood-brain barrier) and CNS (central nervous system) permeability. The Cytochromes P450 (CYPs) models were used to predict metabolism (M). For excretion (E) property, total clearance was evaluated. The toxicity of the identified BTZ analogs was predicted according to AMES toxicity.

## Results and discussion

### Validation test

Before the virtual screening of the BTZ analogs against DprE1, an assessment of the efficiency of the utilized covalent docking protocol to predict the correct docking mode and binding affinity of DprE1 inhibitors was initially evaluated based on test sets I and II, respectively. As well, the DprE1 enzyme was validated based on the Ramachandran plot (S1 Fig). Notably, the Ramachandran plot predicts the structural stereochemical properties of the protein. As illustrated in S1 Fig, 92.1% of the residues fall within the most favored regions, 7.7% in the additionally allowed regions, and 0.3% in the generously allowed regions.

#### Test set I

**Fig 2:**
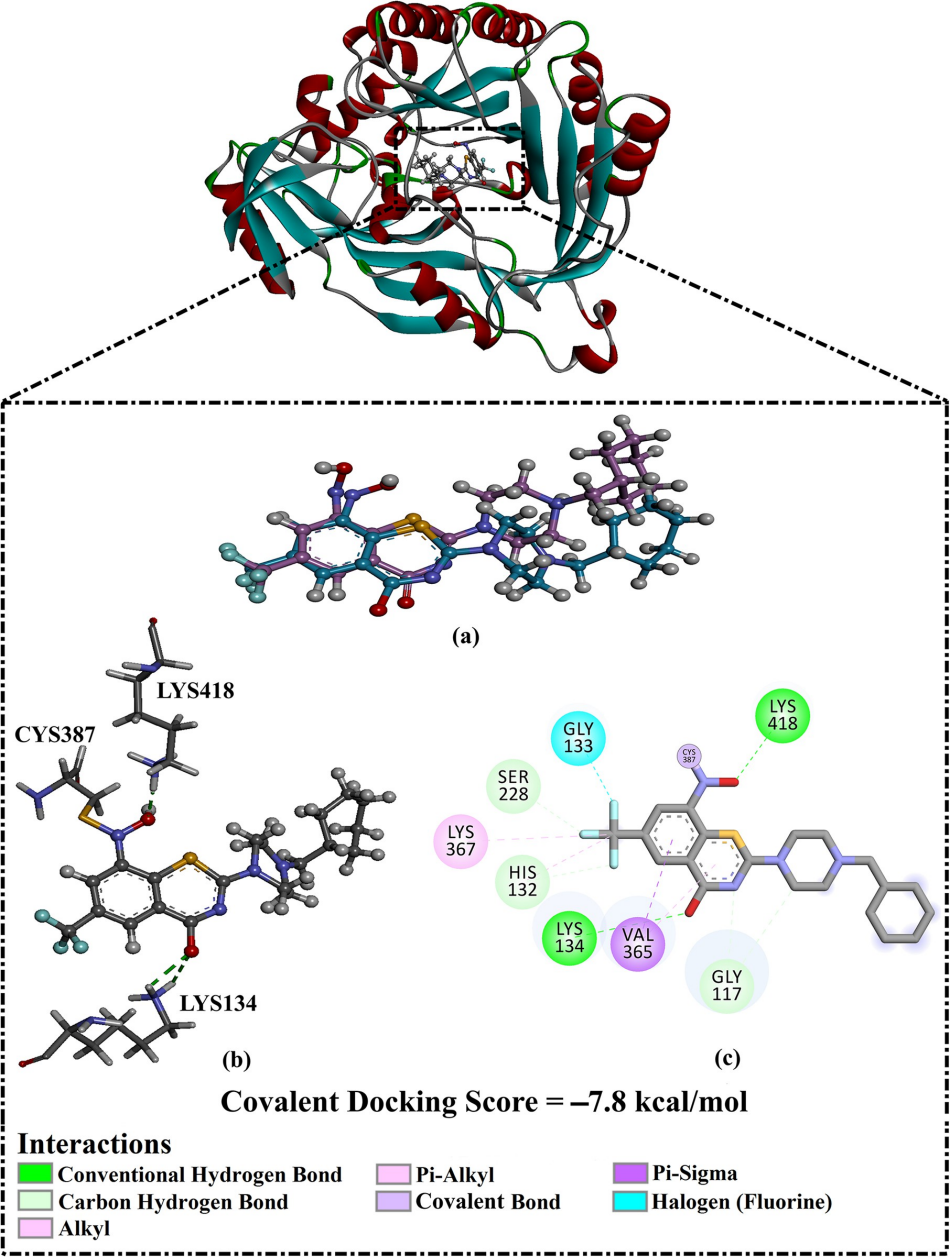
(a) Superimposition illustration of the predicted docking pose (in pink) and the native binding mode of PBTZ169 (in dark cyan), (b) 3D and (c) 2D molecular interactions of the expected docking pose of PBTZ169 complexed with DprE1 enzyme.

The co-crystallized PBTZ169 inhibitor was re-docked utilizing covalent docking. The predicted docking mode was then examined and compared to the native structure to validate the efficiency of the employed docking technique to predict the docking mode of the DprE1 inhibitor (Fig 2). The predicted docking pose of PBTZ169 inside the DprE1 active site was similar to its native binding mode with an RMSD value of 0.73 Å. The computed PBTZ169 docking score in a complex with DprE1 was −7.8 kcal/mol. The good score is attributed to PBTZ169’s ability to form a covalent bond with the SH group of CYS387 (1.16 Å) (Fig 2). In addition, the oxygen of the thiazine-4-one and NO_2_ group exhibited two H-bonds with the NH_3_ group of LYS134 (2.52 Å) and LYS418 (2.00 Å). As well, PBTZ169 demonstrated carbon-hydrogen bonds with HIS132, GLY117, and SER228 (Fig 2). PBTZ169 also displayed halogen, pi-sigma, alkyl, and pi-alkyl interactions with GLY133, VAL362, LYS367, and HIS132 residues, respectively (Fig 2).

#### Test set II

**Fig 3:**
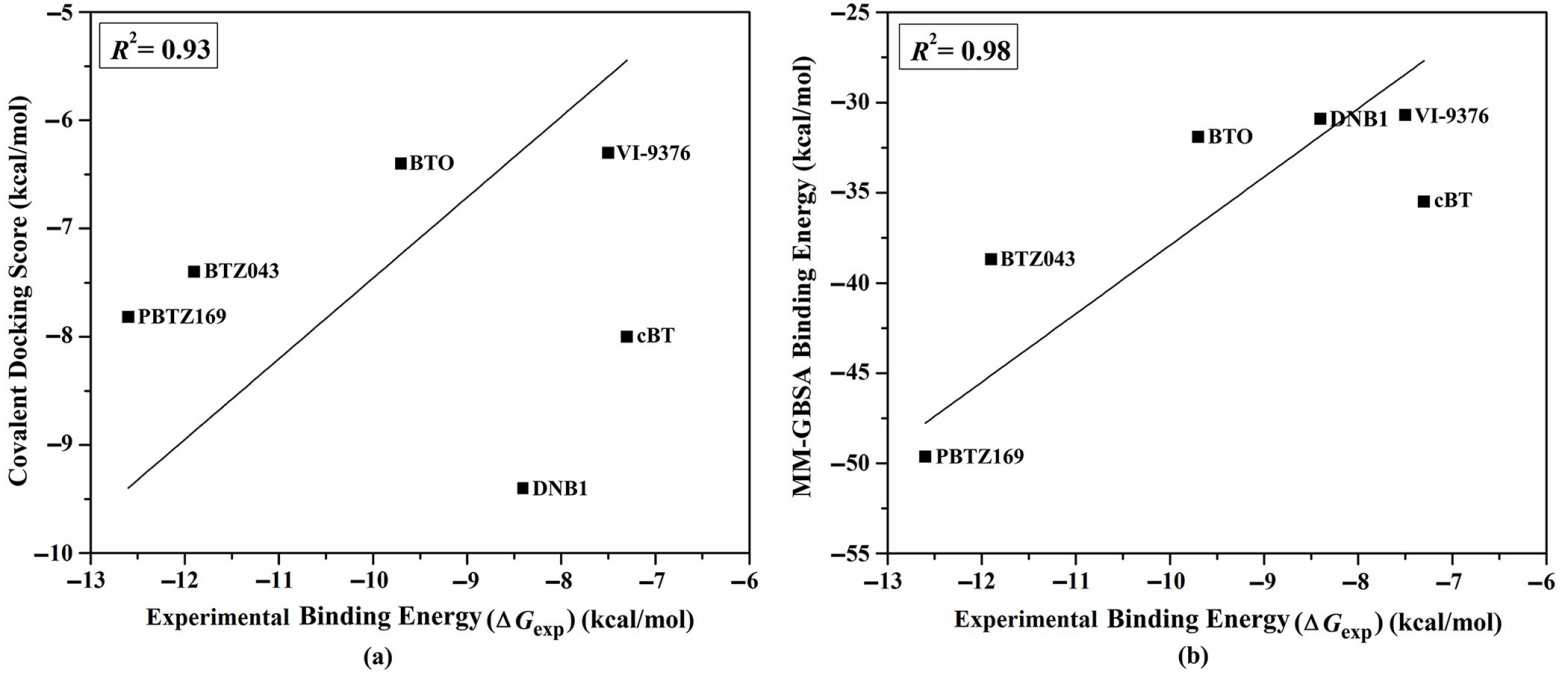
(a) Predicted covalent docking scores and (b) estimated binding energy of test Set II inhibitors complexed with DprE1 enzyme relative to the comparable to the experimental binding energy (Δ*G*_exp_).

Test set II included six irreversible covalent inhibitors–namely BTZ043, PBTZ169, DNB1, VI-9376, BTO, and cBT–with known MIC values with DprE1 [9]. For test set II inhibitors, covalent docking calculations were performed to assess the efficiency of the utilized docking protocol to predict the binding affinity of DprE1 inhibitors. The predicted covalent docking scores were then compared with the corresponding experimental Δ*G*_exp_ (S1 Table and Fig 3A). These docked complexes were subjected to MD simulations over 100 ns. Besides, the corresponding binding energies were computed using the MM-GBSA approach. The estimated binding affinities were compared to Δ*G*_exp_ values (S1 Table and Fig 3B).

Favorable correlations between calculated covalent docking scores and binding affinities as well as Δ*G*_exp_ were observed with correlation coefficient (*R*^2^) values of 0.93 and 0.98, respectively (S1 Table and Fig 3). These findings imply that AutoDock4.2.6 software precisely portends inhibitor-DprE1 binding score and docking pose. Thus, the validated covalent docking protocol was employed to screen potential DprE1 inhibitors.

### Virtual screening of BTZ analogs

Computational techniques are used in various scientific fields, especially in drug discovery and molecular biology [51,52]. Virtual screening (VS) is a powerful computational technique used in the early stages of drug discovery. VS allows researchers to filter large libraries of chemical compounds to identify potential bioactive molecules that could interact with specific biological targets [53–55]. In the current study, an *in-house* database containing 754 BTZ analogs was visually screened using AutoDock4.2.6 software (Fig 1). To reduce computational time and cost, the covalent docking computations of the corrected analogs were executed using fast covalent parameters (i.e., *GA* = 25 and *eval* = 2,500,000). The fast docking scores of these analogs were computed and gathered in S2 Table. As enrolled in S2 Table, 349 out of 754 BTZ analogs demonstrated covalent docking scores less than that of PBTZ169 (calc. −7.8 kcal/mol). These BTZ analogs were selected and subjected to expensive covalent docking computations (i.e., *GA* = 250 and *eval* = 25,000,000). The computed expensive docking scores are listed in S3 Table. According to expensive docking computations, only 94 BTZ analogs manifested covalent docking scores < −9.0 kcal/mol. 2D representations of the most promising 94 BTZ analogs complexed with the DprE1 enzyme are depicted in S2 Fig. Besides, the 2D chemical structures, docking scores, and binding features of the three most potent BTZ analogs with the DprE1 enzyme are summarized in Table 1. It is worth noting that these three analogs were selected based on the calculated MM-GBSA binding energy during the 100 ns MD course detailed in the following sections.

**Table 1:** Predicted covalent docking scores (in kcal/mol) and binding features of PBTZ169 and the most potent BTZ analogs within the DprE1 active site^a^.

No.	PubChem Code	2D Chemical Structure	Covalent Docking Score (kcal/mol)		Binding Features^b^
			Fast	Expensive	
	**PBTZ169** **(PubChem-573-313-86)**	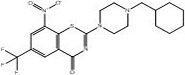	−7.8	−7.8	CYS387 (1.16Å: Covalent bond), LYS134 (2.52 Å: H-bond), LYS418 (2.00 Å: H-bond)
1	**PubChem-155-924-621**	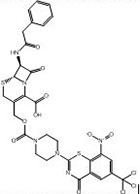	−15.0	−15.7	CYS387 (1.41 Å: Covalent bond), TRP17 (2.76 Å: H-bond), GLN334 (2.63 Å: H-bond), ASP389 (2.23 Å: H-bond), LYS418 (2.11 Å: H-bond)
2	**PubChem-127-032-794**	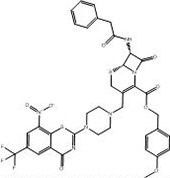	−14.3	−14.7	CYS387 (1.42 Å: Covalent bond), THR118 (2.68 Å: H-bond), HIS132 (2.85 Å: H-bond), LYS418 (1.80 Å: H-bond)
3	**PubChem-155-923-972**	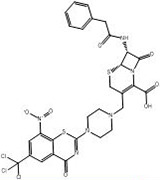	−12.8	−13.3	CYS387 (1.16 Å: Covalent bond), ARG58 (2.08 Å: H-bond), THR118 (2.16 Å: H-bond), GLN326 (2.08 Å: H-bond), GLY328 (2.55 Å: H-bond), LYS418 (2.40 Å: H-bond)

^a^ Data ranked in accordance with the expensive covalent docking scores.

^b^ Intermolecular H-bonds (in Å) and covalent bonds are only mentioned.

According to data listed in S2 Fig and Table 1, most of the examined BTZ analogs showed nearly identical docking poses within the active site, forming an irreversible covalent bond with CYS387 and multiple H-bonds with THR118, HIS132, and LYS418 residues.

**Fig 4:**
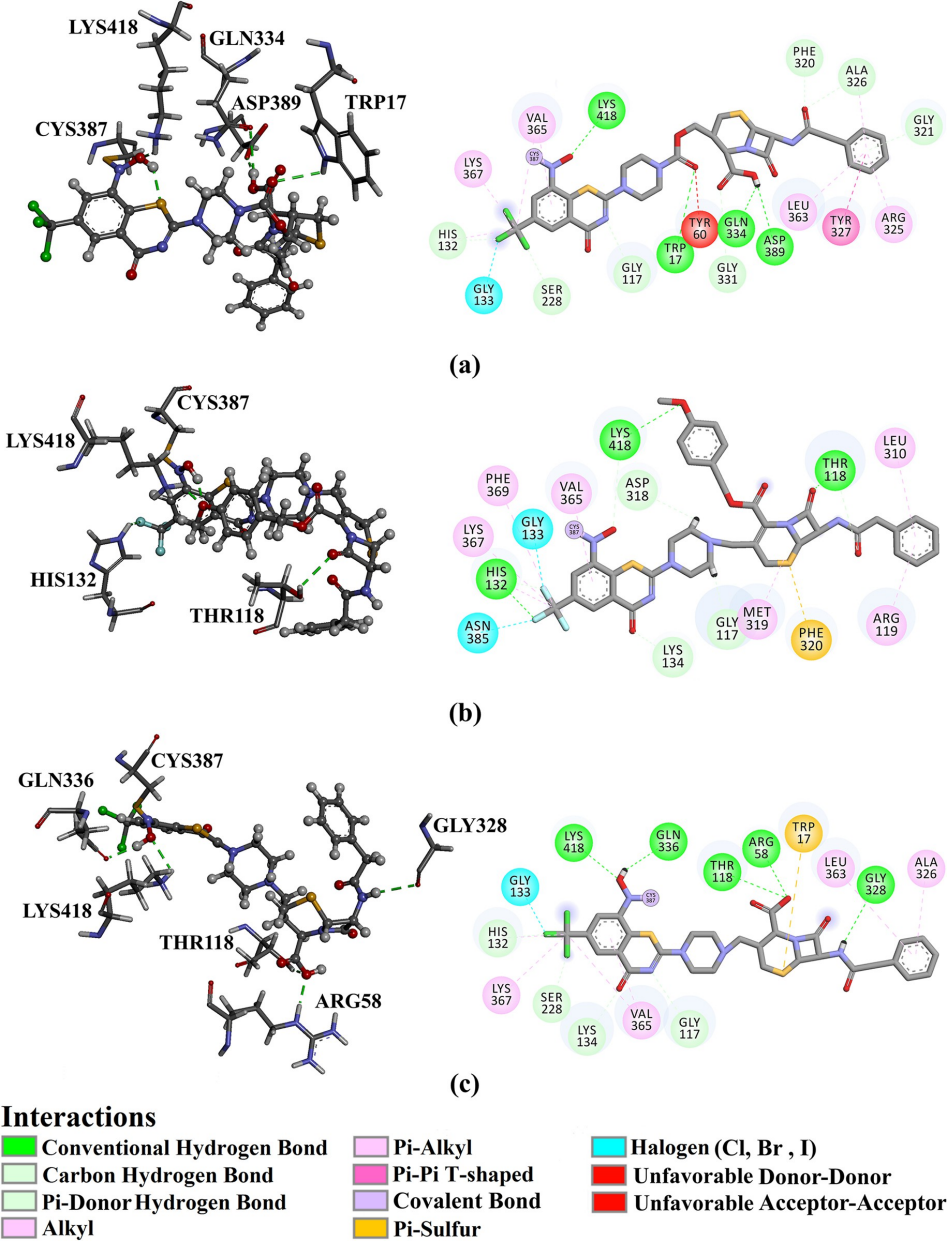
3D and 2D representations of (a) PubChem-155-924-621, (b) PubChem-127-032-794, and (c) PubChem-155-923-972 complexed with DprE1 enzyme.

Fig 4 illustrates 2D and 3D representations of PubChem-155-924-621, PubChem-127-032-794, and PubChem-155-923-972; the nitro (NO_2_) group of the three BTZ analogs formed an irreversible covalent bond with the SH group of CYS387 (1.41, 1.42, and 1.16 Å, respectively) (Fig 4).

PubChem-155-924-621 showed a great binding affinity with DprE1 with a covalent docking score of −15.7 kcal/mol, demonstrating three H-bonds with proximal residues within the active site (Table 1). Examining the docking pose indicated that carbonyl oxygen formed an H-bond with the NH group of TRP17 (2.76 Å) (Table 1 and Fig 4). In addition, the hydroxyl group of the carboxylic acid group exhibited two H-bonds with carbonyl and carboxylic groups of GLN334 (2.63 Å) and ASP389 (2.23 Å) (Table 1 and Fig 4).

PubChem-127-032-794 also demonstrated a promising binding affinity with DprE1 with a covalent docking score of −14.7 kcal/mol. The strong binding of PubChem-127-032-794 with the enzyme is ascribed to the presence of three H-bonds (Table 1 and Fig 4). More precisely, the oxygen of azetidine-one formed an H-bond with the hydroxyl of THR118 (2.86 Å) (Fig 4). An H-bond was predicted between fluorine and the NH group of the HIS132 (2.85 Å) (Fig 4). Furthermore, the oxygen of the anisole ring established an H-bond with the NH_3_ group of LYS418 (1.80 Å) (Table 1 and Fig 4).

Lastly, PubChem-155-923-972 displayed a good binding affinity with the DprE1 with a covalent docking score of −13.3 kcal/mol. The oxygen of the nitro group contributed to two H-bonds with the NH_3_ of LYS418 (2.40 Å) and the carbonyl group of GLN326 (2.08 Å) (Table 1 and Fig 4). The NH group established an H-bond with the carbonyl group of GLY328 (2.55 Å) (Table 1 and Fig 4). In addition, the hydroxyl of the carboxylic group formed two H-bonds with the NH group of ARG58 (2.08 Å) and the OH group of THR118 (2.16 Å).

### Molecular dynamics (MD) simulations

**Fig 5:**
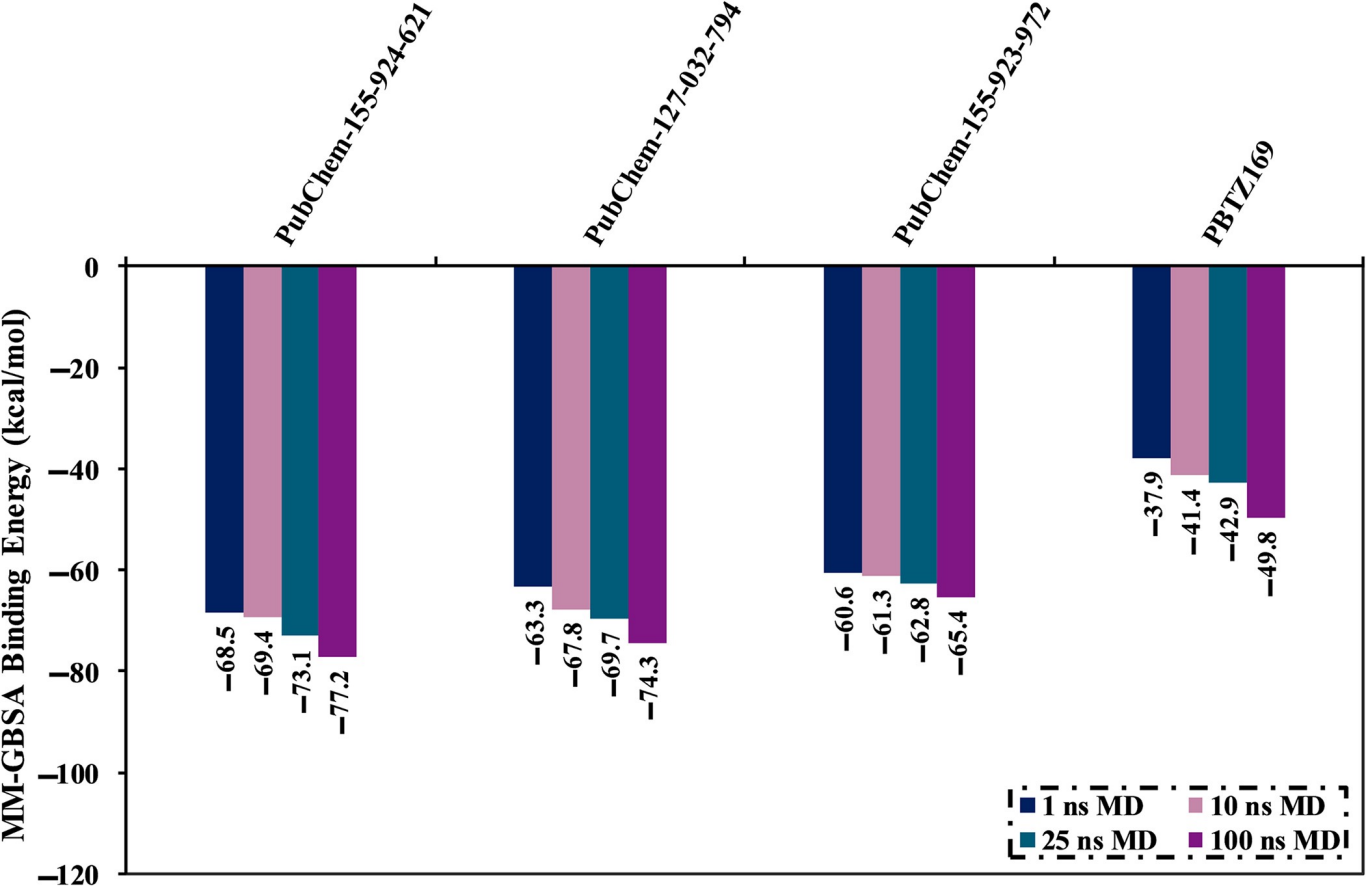
Calculated binding energies of PubChem-155-924-621, PubChem-127-032-794, PubChem-155-923-972, and PBTZ169 complexed with DprE1 enzyme over 1, 10, 25, and 100 ns MD simulations.

MD simulations probe the constancy of ligand-target complexes, the reliability of ligand-target binding affinities, and the conformational elasticity [56,57]. 94 BTZ analogs with covalent docking scores < −9.0 kcal/mol were chosen and submitted to MD simulations. It is worth noting that the docking score of –9.0 kcal/mol was chosen as the threshold value to shortlist the potent BTZ analogs. To minimize time and computational costs, the MD simulations were run over 1 ns. The corresponding binding affinities are listed in S4 Table. From S4 Table, 33 out of 94 BTZ analogs demonstrated binding energies (Δ*G*_binding_) less than −50.0 kcal/mol. To obtain more credible DprE1 binding affinities, these 33 BTZ analogs were further submitted to MD simulations throughout 10 ns. The corresponding binding affinities were evaluated and collected in S5 Table. Notably, 23 BTZ analogs manifested binding energies < −50.0 kcal/mol (S5 Table). The MD simulations of these analogs complexed with DprE1 were prolonged to 25 ns. Additionally, the corresponding binding affinities were calculated (S6 Table). Interestingly, out of these 23 BTZ analogs, PubChem-155-924-621, PubChem-127-032-794, and PubChem-155-923-972 exposed binding energies (Δ*G*_binding_) with values of −73.1, −69.7, and −62.8 kcal/mol, respectively, compared to PBTZ169 (Δ*G*_binding_ = −42.9 kcal/mol). In order to obtain more rigorous binding energies, MD simulations for those three analogs were elongated to 100 ns. Moreover, the corresponding binding affinities were calculated (see Fig 5). Interestingly, there was no noticeable difference in the estimated binding energies for the identified BTZ analogs complexed with DprE1 throughout the 25 and 100 ns MD simulations. Compared to PBTZ169 (Δ*G*_binding_ = −49.8 kcal/mol), the computed binding energies of PubChem-155-924-621, PubChem-127-032-794, and PubChem-155-923-972 were −77.2, −74.3, and −65.4 kcal/mol, respectively.

**Fig 6:**
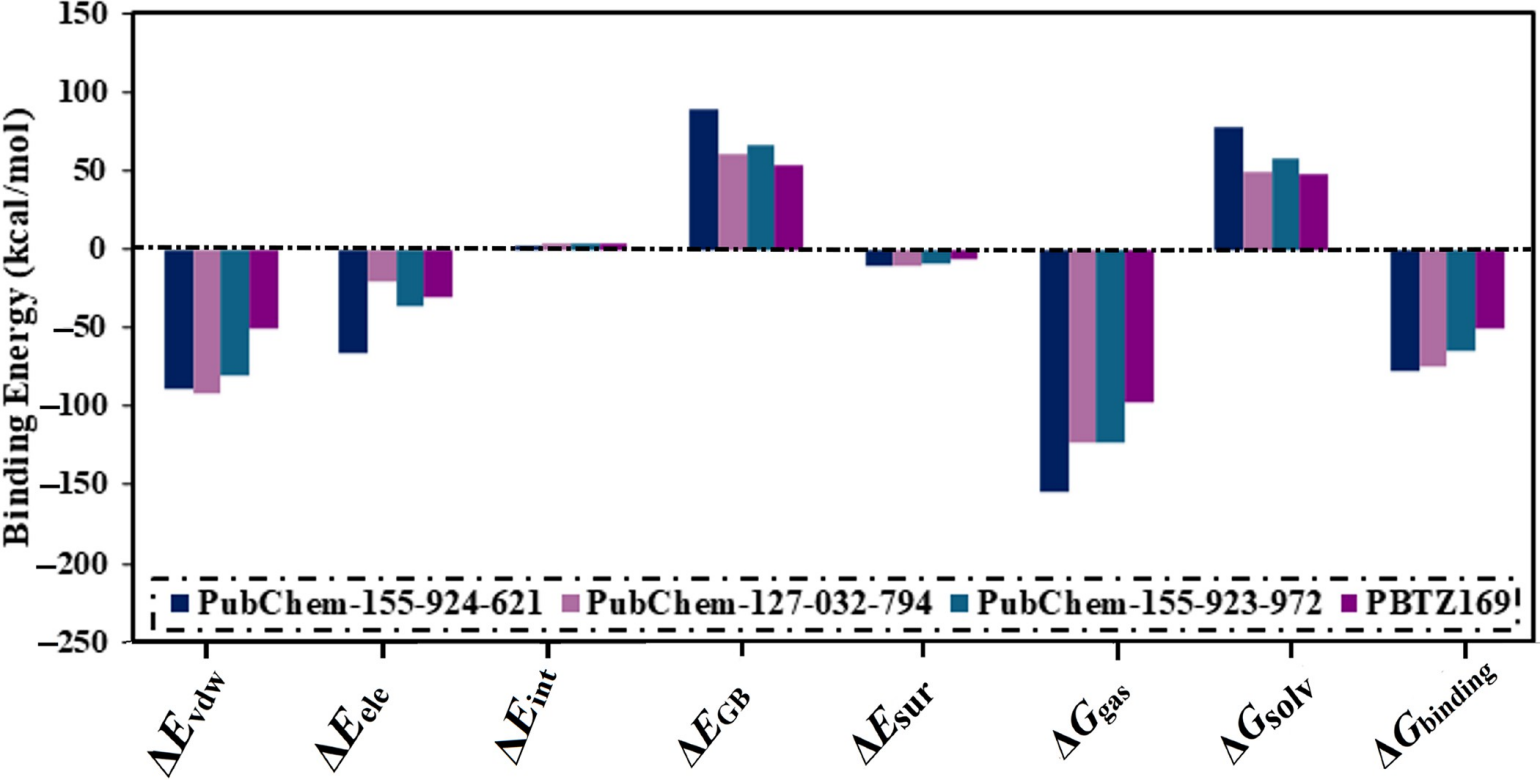
Binding energy decomposition for PubChem-155-924-621, PubChem-127-032-794, PubChem-155-923-972, and PBTZ169 complexed with DprE1 enzyme throughout 100 ns MD simulations.

To examine the binding for these selected BTZ analogs, the computed binding affinities were further decomposed (Fig 6). From Fig 6, Δ*E*_vdW_ was the predominant contributor for PubChem-155-924-621, PubChem-127-032-794, PubChem-155-923-972, and PBTZ169 complexed with DprE1 with an average value of −89.3, −91.3, −79.6, and −50.7 kcal/mol, respectively (Fig 6). In addition, Δ*E*_ele_ was appropriate with values of −66.4, −19.9, −35.6, and −29.8 kcal/mol for PubChem-155-924-621-, PubChem-127-032-794-, PubChem-155-923-972-, and PBTZ169-DprE1 complexes, respectively (Fig 6). By comparing the average values of Δ*E*_vdW_ and Δ*E*_ele_, it was indicated that Δ*E*_vdW_ approximately was one and a half-fold of Δ*E*_ele_. Notably, Δ*E*_int_ has a tiny contributor for the investigated BTZ analogs complexed with the enzyme, with values ranging from 2.8 to 4.3 kcal/mol (Fig 6).

**Fig 7:**
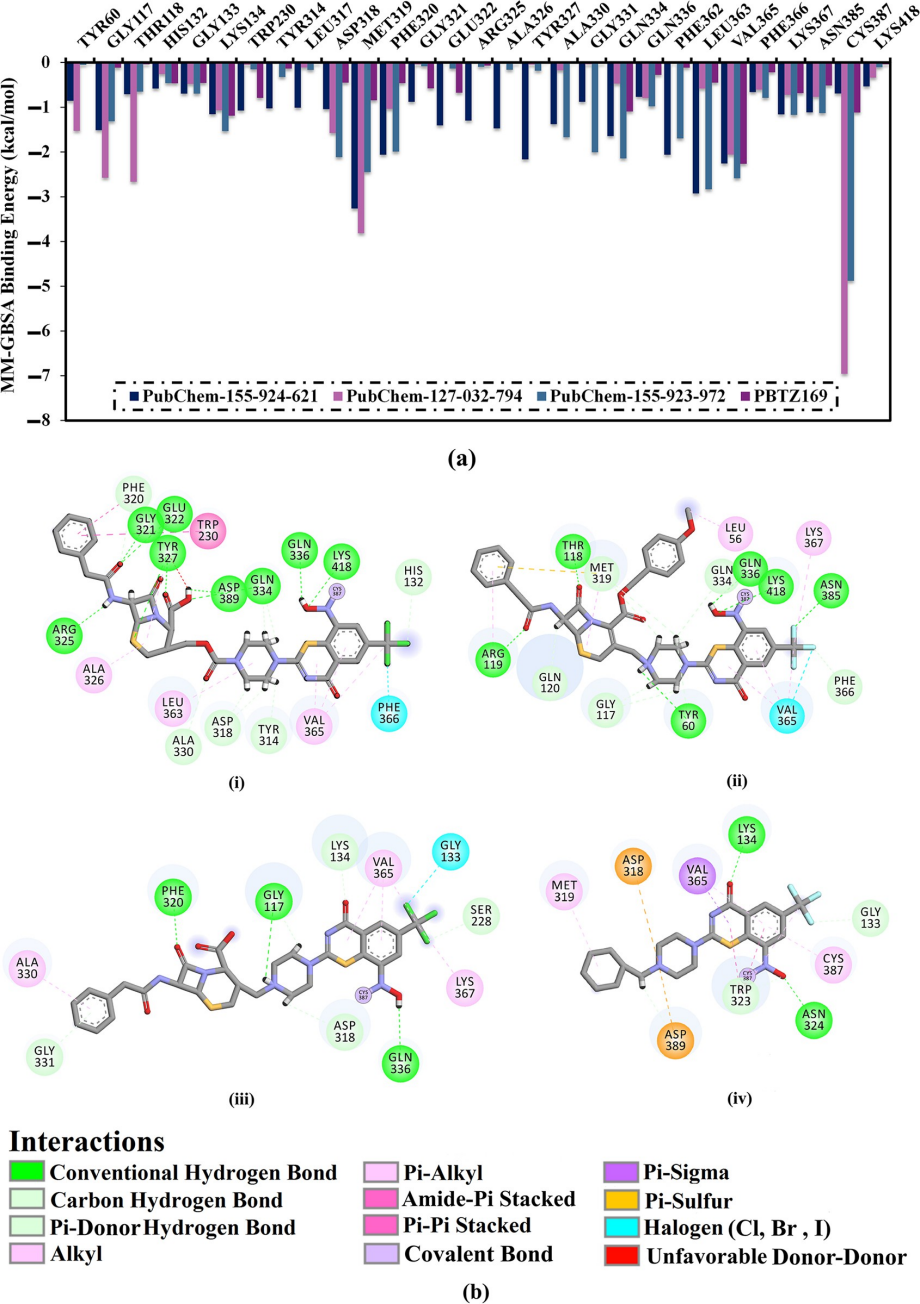
(a) The energy contribution of the most significant residues to the overall binding energy and (b) 2D molecular interactions of binding modes of (i) PubChem-155-924-621, (ii) PubChem-127-032-794, (iii) PubChem-155-923-972, and (iv) PBTZ169 complexed with DprE1 enzyme according to the final snapshot throughout a 100 ns MD simulation.

In addition, a per-residue energy decomposition analysis was implemented to investigate the amino acid residues that exhibit prominent participation with DprE1. Only residues with binding energy contributions lower than −0.50 kcal/mol were considered and are displayed in Fig 7A. MET319, PHE320, GLN334, VAL365, and CYS387 interact with PubChem-155-924-621, PubChem-127-032-794, and PubChem-155-923-972, and PBTZ169. CYS387 had a significant role in Δ*G*_binding_ with values of −0.69, −6.95, −4.88, and −1.12 kcal/mol for PubChem-155-924-621-, PubChem-127-032-794-, PubChem-155-923-972-, and PBTZ169-DprE1 complexes, respectively (Fig 7A). Inspecting the final trajectory of the BTZ analogs and PBTZ169 complexed with DprE1 indicated preserved H-bonds with key residues over the MD simulations (Fig 7B). Noteworthy, the three investigated BTZ analogs complexed with DprE1 have approximately identical interaction patterns with proximal residues, implying propinquity in the binding mode of these studied complexes.

### Post-MD analyses

To inspect the conformational changes and the stability of the most promising BTZ analogs complexed with DprE1, post-MD analyses were employed during the 100 ns MD course. Post-MD analyses involved the binding energy per trajectory, the number of H-bonds, CoM distance, RMSD, RMSF, Rg, and SASA [58,59].

#### Binding energy per trajectory

**Fig 8:**
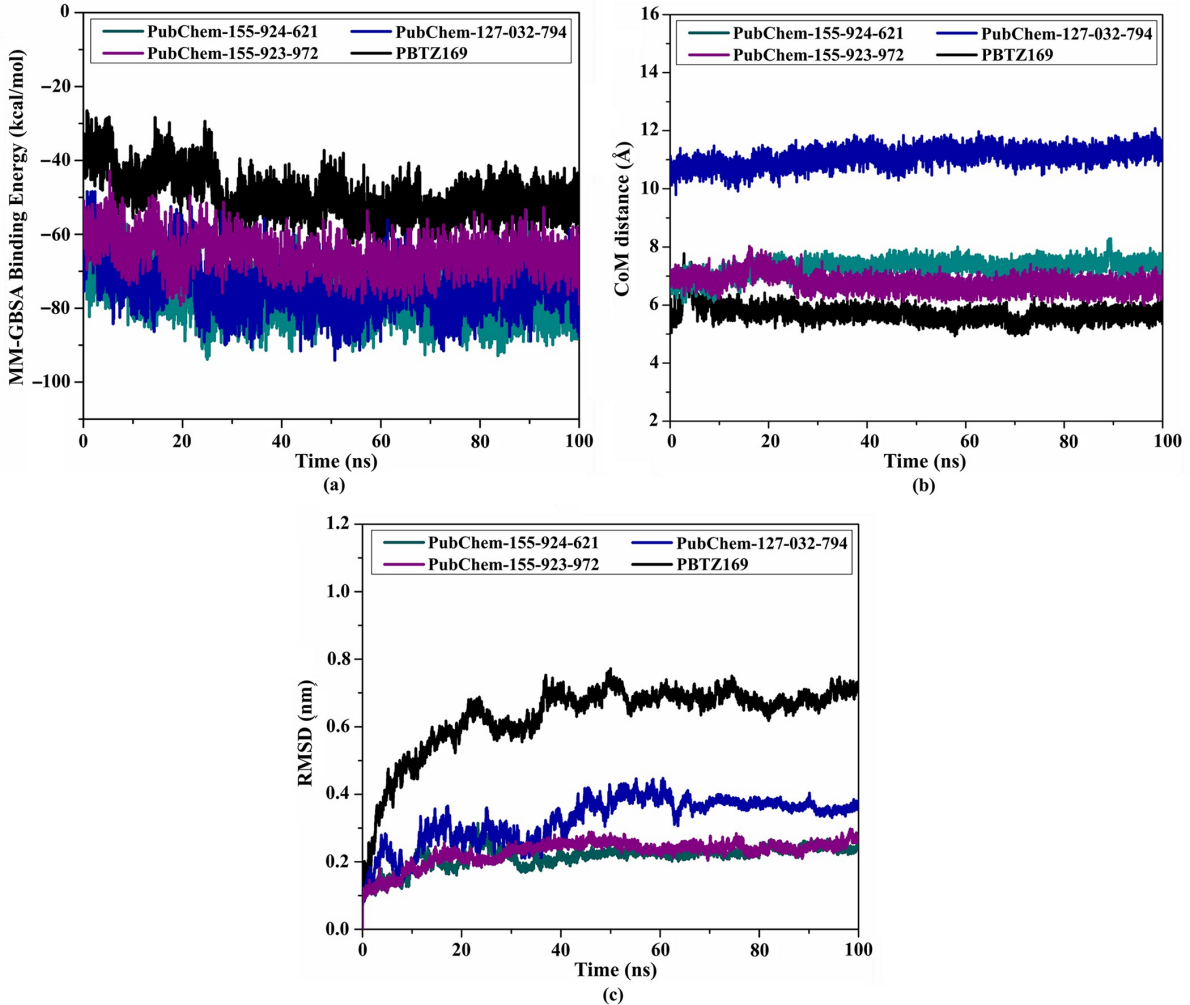
(a) Estimated binding energy per trajectory, (b) CoM distances, and (c) RMSD of the backbone atoms from the initial conformational of PubChem-155-924-621 (in dark cyan), PubChem-127-032-794 (in blue), PubChem-155-923-972 (in violet), and PBTZ169 (in black) towards DprE1 enzyme during the 100 ns MD course.

To estimate the constancy of PBTZ169, PubChem-155-924-621, PubChem-127-032-794, and PubChem-155-923-972 complexed with the DprE1 active site, the correlation between MM-GBSA binding affinity and time was executed throughout the simulation time of 100 ns (Fig 8A). The most intriguing aspect of Fig 8A is the high stability of PubChem-155-924-621, PubChem-127-032-794, PubChem-155-923-972, and PBTZ169 complexed with the enzyme with Δ*G*_binding_ values of −77.2, −74.3, −65.4, and −49.8 kcal/mol, respectively. Based on these outcomes, the three BTZ analogs and PBTZ169 complexed with DprE1 kept immutability over 100 ns MD course.

#### CoM distance

The center-of-mass (CoM) distance between the selected BTZ analogs and CYS387 was adopted throughout a 100 ns MD simulation to understand the constancy of DprE1-analog complexes (Fig 8B). As depicted in Fig 8B, the gauged CoM distance was steady for PubChem-155-924-621, PubChem-127-032-794, PubChem-155-923-972, and PBTZ169 complexed with DprE1 with an average value of 7.3, 11.1, 6.8, and 5.7 Å, respectively. These outcomes demonstrated that PBTZ169 and the investigated BTZ analogs bind tightly with the DprE1 enzyme.

#### Root-mean-square deviation (RMSD)

To examine the structural and positional variations inside the DprE1 active site and inspect the structural stabilization of the complexes, RMSD was measured (Fig 8C). The average RMSD values for PubChem-155-924-621, PubChem-127-032-794, PubChem-155-923-972, and PBTZ169 complexed with DprE1 enzyme were 0.23, 0.32, 0.23, and 0.63 nm, respectively (Fig 8C). The RMSD analysis displayed that the investigated complexes tended to exist in an equilibrium state after 20 ns until the end of the 100 ns MD simulations, and the proposed analogs were tightly bonded without changing the overall topology of DprE1.

#### Root-mean-square fluctuation (RMSF)

**Fig 9:**
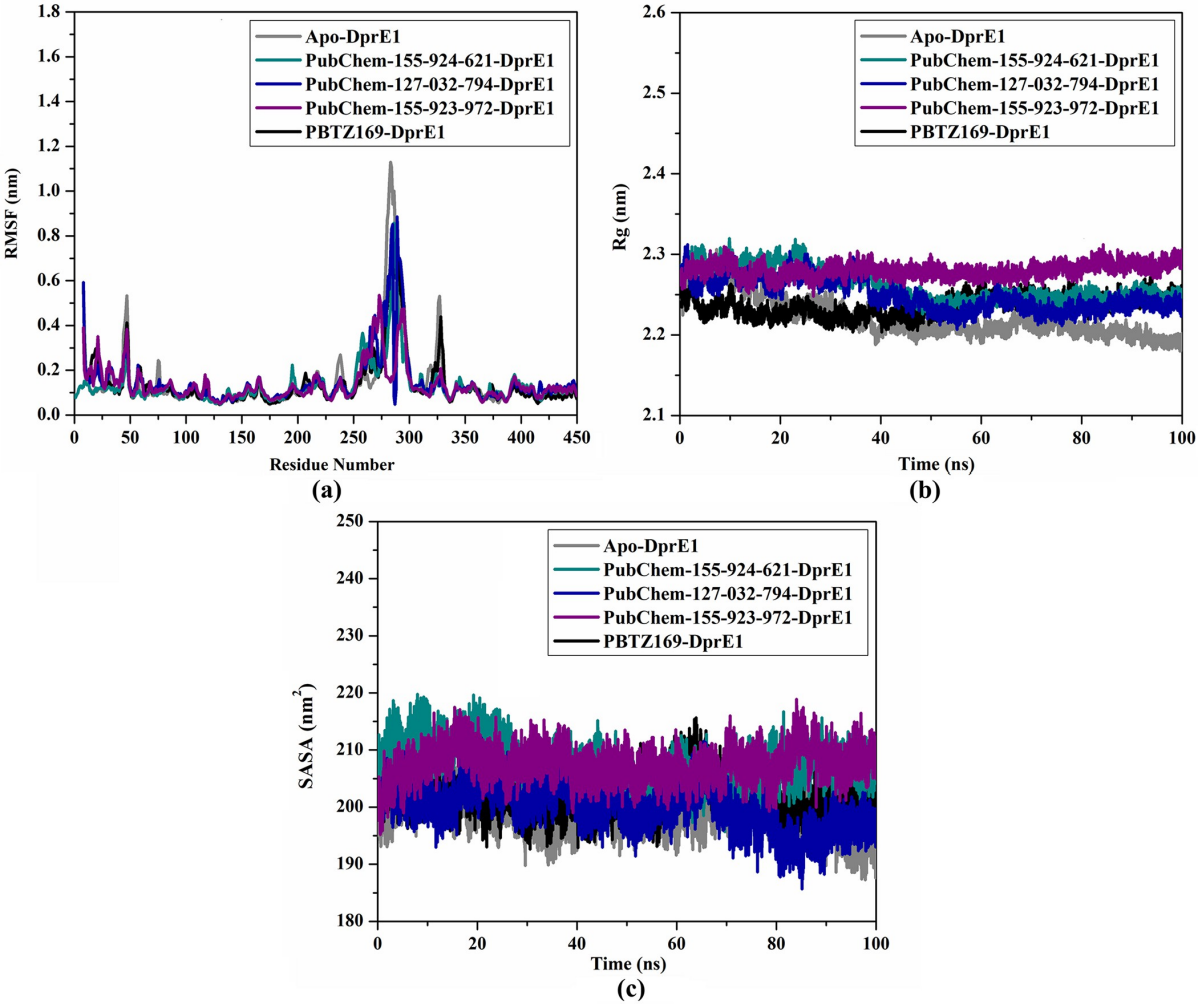
Estimated (a) RMSF, (b) Rg, and (c) SASA for apo- (in grey), PubChem-155-924-621- (in dark cyan), PubChem-127-032-794- (in blue), PubChem-155-923-972- (in violet), and PBTZ169-DprE1 (in black) over 100 ns MD simulations.

To assess the conformational variation and stability of the backbone in apo-, PubChem-155-924-621-, PubChem-127-032-794-, PubChem-155-923-972-, and PBTZ169-DprE1, the RMSF analysis of C_α_ atoms was measured (Fig 9A). As shown in Fig 9A, the residues remained stable for the investigated systems throughout the 100 ns MD simulations. Nevertheless, a greater fluctuation was observed at residues from 260 to 290 and from 310 to 330, indicating great flexibility in these regions. The average RMSF values for apo-, PubChem-155-924-621-, PubChem-127-032-794-, PubChem-155-923-972-, and PBTZ169-DprE1 were found to be 0.14, 0.13, 0.15, 0.14, and 0.14 nm, respectively (Fig 9A).

#### Radius of gyration (Rg)

The Rg analysis was executed to inspect the compactness of DprE1 in its apo form and complex form with identified BTZ analogs over 100 ns MD simulation. The Rg analysis provided insights into the overall folding and unfolding behavior of the DprE1 structure upon binding with the identified BTZ analogs (Fig 9B). The average Rg values were 2.22, 2.26, 2.25, 2.28, and 2.24 nm for apo-, PubChem-155-924-621-, PubChem-127-032-794-, PubChem-155-923-972-, and PBTZ169-DprE1, respectively (Fig 9B). The Rg results indicated that DprE1 maintained its compactness when bound to the BTZ analogs and PBTZ169 throughout 100 ns MD simulations. These findings unveiled that the binding of PubChem-155-924-621, PubChem-127-032-794, PubChem-155-923-972, and PBTZ169 significantly stabilized the DprE1 structure.

*Solvent-accessible surface area (SASA)*. SASA analysis was performed to gain a deeper understanding of the interactions between the complexes and the solvent over the course of the 100 ns MD simulations. Fig 9C illustrates the graph for SASA *vs*. simulation time for apo-, PubChem-155-924-621-, PubChem-127-032-794-, PubChem-155-923-972-, and PBTZ169-DprE1. As depicted in Fig 9C, the average SASA values were found to be 198.91, 207.56, 199.90, 207.46, and 202.04 nm^2^ for the apo-, PubChem-155-924-621-, PubChem-127-032-794-, PubChem-155-923-972-, and PBTZ169-DprE1, respectively. These findings demonstrated that no significant changes in the SASA values were observed for DprE1 due to its complexation with BTZ analogs. These results revealed that the BTZ analogs did not notably affect the solvent exposure of the DprE1 enzyme.

#### H-bond number

**Fig 10:**
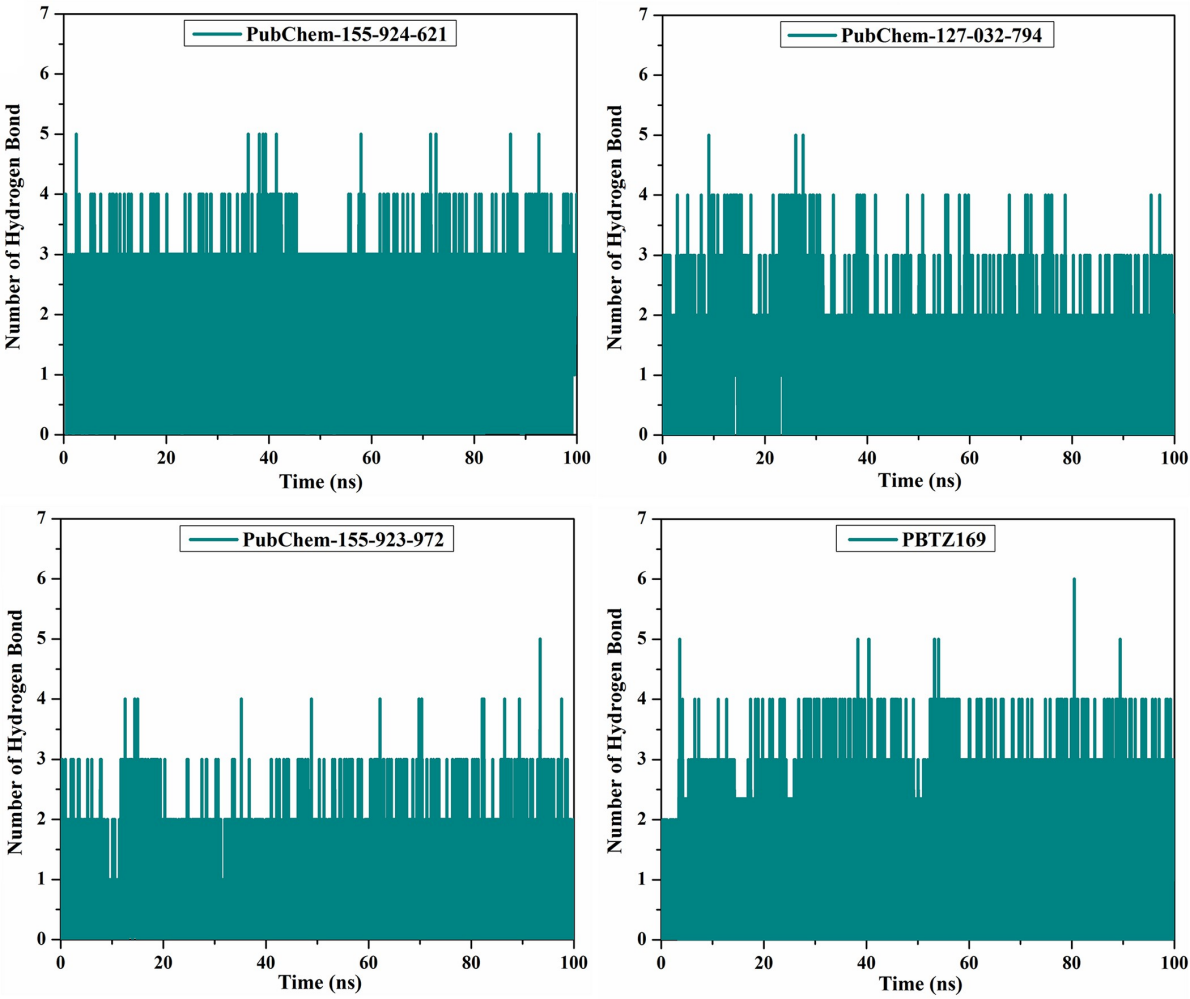
Number of H-bonds for PubChem-155-924-621, PubChem-127-032-794, PubChem-155-923-972, and PBTZ169 complexed with DprE1 enzyme during the 100 ns MD simulations.

H-bond analysis was performed to estimate the number of H-bonds between the identified BTZ analogs and DprE1 enzyme over a 100 ns MD course (Fig 10). Interestingly, PubChem-155-924-621, PubChem-127-032-794, PubChem-155-923-972, and PBTZ169 complexed with DprE1 revealed an average number of H-bonds was 2, 1, 1, and 2, respectively. Generally, these post-dynamics studies endorsed the inherent steadiness of the investigated BTZ analogs in complex with DprE1 enzyme over a 100 ns MD course.

### Physicochemical features

**Fig 11:**
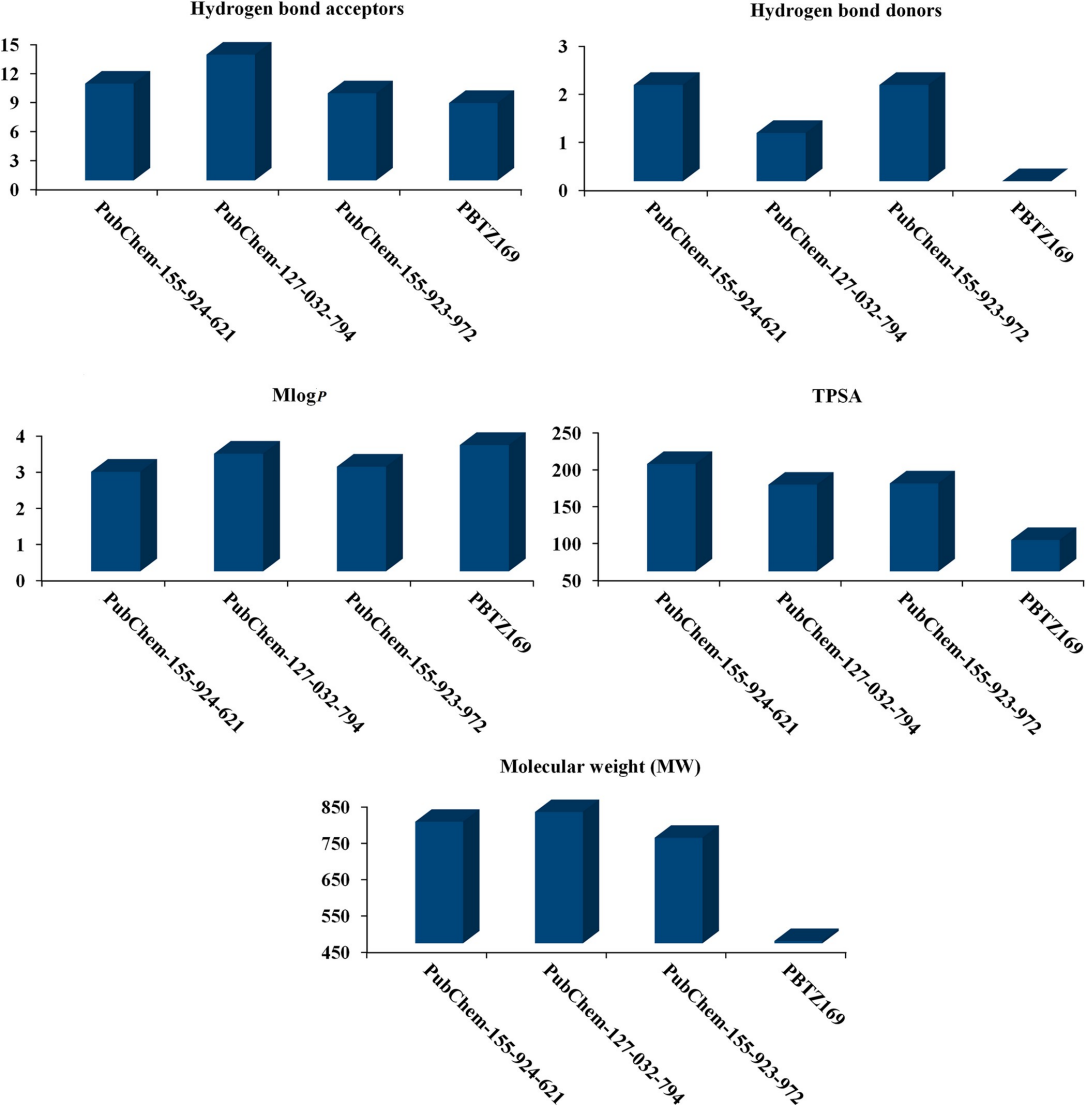
Estimated physiochemical features of PubChem-155-924-621, PubChem-127-032-794, PubChem-155-923-972, and PBTZ169 as DprE1 inhibitors.

The druggability of each compound was determined by its physicochemical characteristics [60]. To predict the bioavailability and physicochemical features of the examined BTZ analogs as DprE1 inhibitors, the SWISS-ADME server was used. Fig 11 illustrates the anticipated physicochemical properties of the examined BTZ analogs. MLog*P* values for PubChem-155-924-621, PubChem-127-032-794, PubChem-155-923-972, and PBTZ169 were 2.75, 3.25, 2.89, and 3.49, respectively, demonstrating that these analogs exhibited high lipophilicity. Molecular weight (MW) ranged from 750 to 850 dalton (Fig 11). However, PBTZ169 demonstrated MW with a value of 456.5 dalton. Additionally, the number of H-bond acceptors (HBA) for PubChem-155-924-621, PubChem-127-032-794, PubChem-155-923-972, and PBTZ169 was 10, 13, 9, and 8, respectively (Fig 11). Notably, this increase in molecular weight and hydrogen bond acceptors is unlikely to significantly impact molecule transmission and diffusion, as many FDA-approved drugs have deviated from the conventional molecular weight limit of 500 and the number of hydrogen bond acceptors of 10 [61]. The number of HBD for PubChem-155-924-621, PubChem-127-032-794, PubChem-155-923-972, and PBTZ169 was less than 5 (Fig 11). The TPSA values of the identified analogs ranged from 110 to 200 Å^2^, demonstrating their good oral absorption and membrane permeability [62].

### Pharmacokinetic characteristics

The pkCSM server was employed to evaluate the pharmacokinetic properties of identified BTZ analogs. ADMET properties of the investigated analogs are compiled in Table 2. Log*S* values of PubChem-155-924-621, PubChem-127-032-794, PubChem-155-923-972, and PBTZ169 were −2.99, −4.1, −2.9, and −5.6, respectively, and were considered to be reasonably soluble (Table 2). PubChem-155-924-621, PubChem-127-032-794, PubChem-155-923-972, and PBTZ169 demonstrated log BB with values of −2.4, −2.1, −2.0, and −0.9, respectively. The log PS value for CNS permeability ranged from −4 to −2, indicating impenetrability. The majority of the identified analogs were predicted to be unable to cross the CNS or permeate the BBB barrier (Table 2). In terms of metabolism (M), PBTZ169 and PubChem-127-032-794 were inhibitors of CYP3A4 enzyme, whereas PubChem-155-924-621 and PubChem-155-923-972 were non-inhibitors of CYP3A4 enzyme (Table 2). Additionally, the result of the metabolism test indicates that PBTZ169 and the identified analogs act as substrates for the CYP3A4 enzyme (Table 2). For excretion (E) property, the estimated total clearance for PubChem-155-924-621, PubChem-127-032-794, PubChem-155-923-972, and PBTZ169 was 0.26, −0.3, −0.53, and 0.02, respectively (Table 2). According to toxicity (T) analysis, PubChem-155-924-621 and PubChem-155-923-972 were non-toxic, while PBTZ169 and PubChem-127-032-794 were toxic (Table 2). All the examined BTZ analogs revealed satisfied outcomes, with some values even better than PBTZ169, as indicated in Table 2.

**Table 2:** Pharmacokinetic and toxicity profile for the identified BTZ analogs and PBTZ169.

PubChem Code	Absorption (A)	Distribution (D)		Metabolism (M)		Excretion (E)	Toxicity (T)
	Log*S*	Blood Brain Barrier (BBB)	CNS Permeability	CYP3A4 Substrate	CYP3A4 Inhibitor	Total Clearance	AMES Toxicity
**PBTZ169**	−5.6	−0.9	−2.1	Yes	Yes	0.02	Yes
**PubChem-155-924-621**	−2.99	−2.4	−3.6	Yes	No	0.26	No
**PubChem-127-032-794**	−4.1	−2.1	−3.5	Yes	Yes	−0.3	Yes
**PubChem-155-923-972**	−2.9	−2.0	−3.6	Yes	No	−0.53	No

## Conclusions

DprE1 is reported to be a crucial and very effective target for inhibiting tuberculosis (TB). As a prospective DprE1 irreversible covalent inhibitor, BTZ analogs have received increased attention because they demonstrate additive activity when combined with other anti-TB drugs. To identify effective DprE1 inhibitors, a library of 754 BTZ analogs was constructed and screened against DprE1 using covalent docking computations. Based on covalent docking scores, the most promising BTZ analogs with docking scores < −9.0 kcal/mol were subjected to MD simulations, followed by binding energy evaluations using the MM-GBSA approach. In accordance with the MM-GBSA computations, PubChem-155-924-621, PubChem-127-032-794, and PubChem-155-923-972 demonstrated promising Δ*G*_binding_ with values of −77.2, −74.3, and −65.4 kcal/mol, respectively, compared to PBTZ169 (Δ*G*_binding_ = −49.8 kcal/mol). Post-dynamics analyses showed that the identified BTZ analogs demonstrated high stability over 100 ns MD simulations. The predicted physicochemical and ADMET properties of the identified BTZ analogs proposed the promising oral bioavailability of PubChem-155-924-621, Pub-Chem-127-032-794, and PubChem-155-923-972 as potential tuberculosis drug candidates. These findings indicated that PubChem-155-924-621, PubChem-127-032-794, and PubChem-155-923-972 may be potent DprE1 inhibitors that warrant additional *in-vitro* and *in-vivo* assays. The current *in-silico* results established that these compounds are recommended for clinical investigations against tuberculosis.

## Supporting information

S1 Fig Ramachandran plot for validating the investigated DprE1 enzyme using the PROCHECK server. (DOCX)

S2 Fig 2D representations of the binding modes of the top 94 potent BTZ analogs complexed with DprE1 enzyme. (DOCX)

S1 Table Chemical structures, minimum inhibitory concentration (MIC) value, computed covalent docking scores, and MM-GBSA binding energies (in kcal/mol) over 100 ns MD simulations for the test set II inhibitors towards DprE1 enzyme. (DOCX)

S2 Table Calculated fast covalent docking scores (in kcal/mol) for PBTZ169 and the top 754 potent BTZ analogs against DprE1 active site. (DOCX)

S3 Table Calculated fast and expensive covalent docking scores (in kcal/mol) for PBTZ169 and the top 349 potent BTZ analogs against the DprE1 active site. (DOCX)

S4 Table Estimated fast and expensive covalent docking scores and MM-GBSA binding energies (in kcal/mol) over 1 ns MD simulations for PBTZ169 and the top 94 potent BTZ analogs within the DprE1 active site. (DOCX)

S5 Table Estimated fast and expensive covalent docking scores and MM-GBSA binding energies (in kcal/mol) over 1 and 10 ns MD simulations for PBTZ169 and the top 33 potent BTZ analogs within DprE1 active site. (DOCX)

S6 Table Estimated fast and expensive covalent docking scores and MM-GBSA binding energies (in kcal/mol) over 1, 10, and 25 ns MD simulations for PBTZ169 and the top 23 potent BTZ analogs within DprE1 active site. (DOCX)

## Abbreviations

DprE1: Decaprenylphosphoryl-D-ribose oxidase
TB: Tuberculosis
M. tuberculosis: *Mycobacterium tuberculosis*
BTZ: Benzothiazinone
InChIKey: International Chemical Identifier key
3D: Three-dimensional
GA: Genetic algorithm
eval: Energy evaluations
MD: Molecular dynamics
GAFF2: General AMBER force field
RESP: Restrained electrostatic potential
MM-GBSA: Molecular mechanics-generalized Born surface area
G: Energy term
*E*_MM_: Molecular mechanics gas-phase energy
*G*_solv_: Solvation energy
*E*_int_: Internal molecular mechanics energy
*E*_vdW_: Van der Waals energy
*E*_ele_: Electrostatic energy
MW: Molecular weight
HBD: H-Bond donors
TPSA: Topological polar surface area
HBA: H-Bond acceptors
Mlog*P*: n-Octanol/water partition coefficient
ADMET: Absorption distribution metabolism excretion toxicity
log*S*: Water solubility
BBB: Blood-brain barrier
CNS: Central nervous system
CYP: Cytochromes P450
Δ*G*_exp_: Experimental binding energy
*R* ^2^: Correlation coefficient
CoM: Center-of-mass
RMSD: Root-mean-square deviation
RMSF: Root-mean-square fluctuation
Rg: Radius of gyration
SASA: Solvent accessible surface area
