# Supplementary material for: Benzothiazinone analogs as Anti-Mycobacterium tuberculosis DprE1 irreversible inhibitors: Covalent docking, validation, and molecular dynamics simulations
Source: PLoS One. 2024 Nov 25;19(11):e0314422. doi: 10.1371/journal.pone.0314422 (PMC11588222; doi:10.1371/journal.pone.0314422)
Supplement: S1 Fig — (DOCX) [file pone.0314422.s001.docx]

**
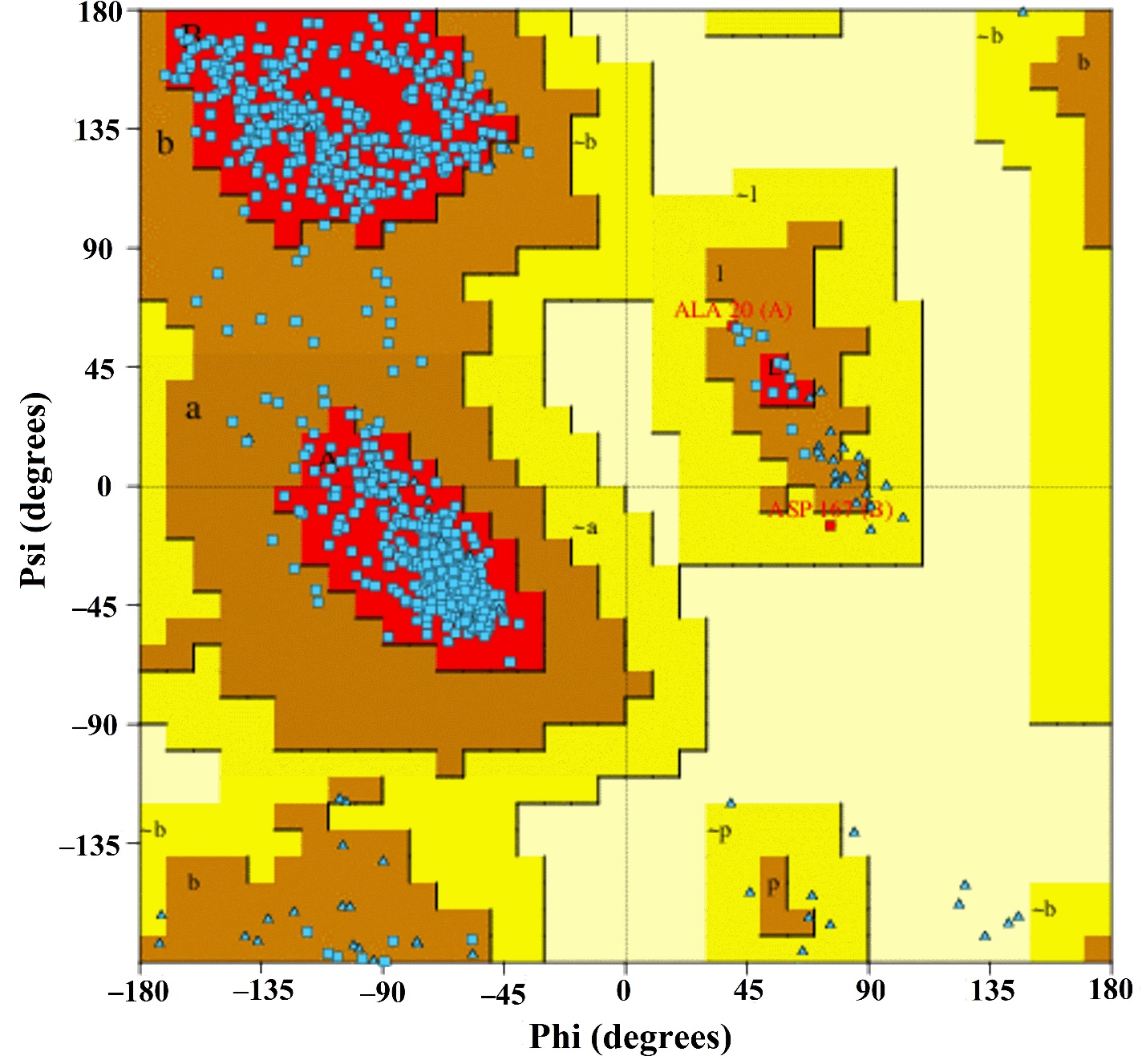
**

**S1 Fig.** Ramachandran plot for validating the investigated DprE1 enzyme using the PROCHECK server.
