# Supplementary material for: Benzothiazinone analogs as Anti-Mycobacterium tuberculosis DprE1 irreversible inhibitors: Covalent docking, validation, and molecular dynamics simulations
Source: PLoS One. 2024 Nov 25;19(11):e0314422. doi: 10.1371/journal.pone.0314422 (PMC11588222; doi:10.1371/journal.pone.0314422)
Supplement: S2 Fig — (DOCX) [file pone.0314422.s002.docx]

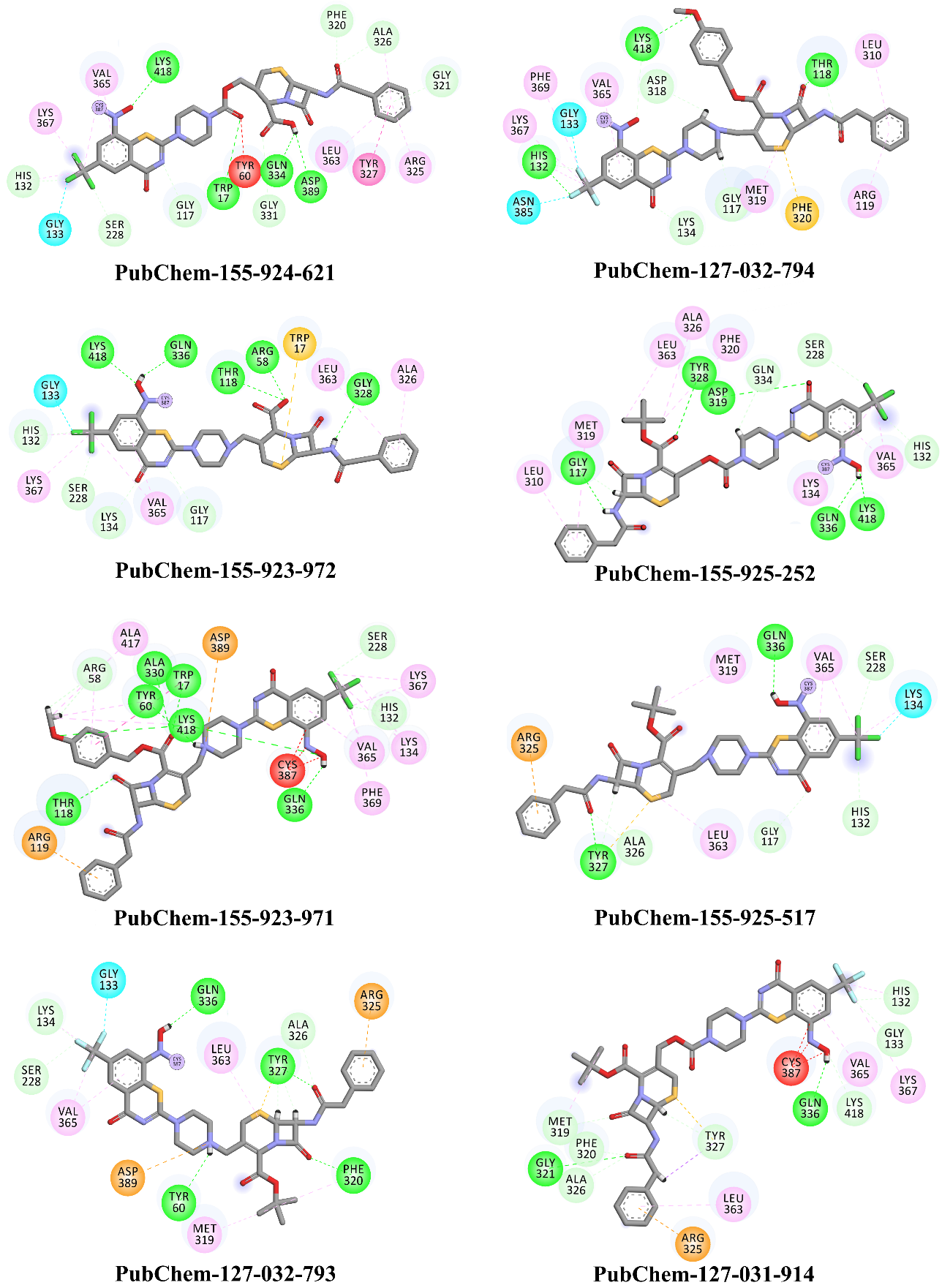


**S2 Fig**. 2D representations of the binding modes of the top 94 potent BTZ analogs complexed with DprE1 enzyme.


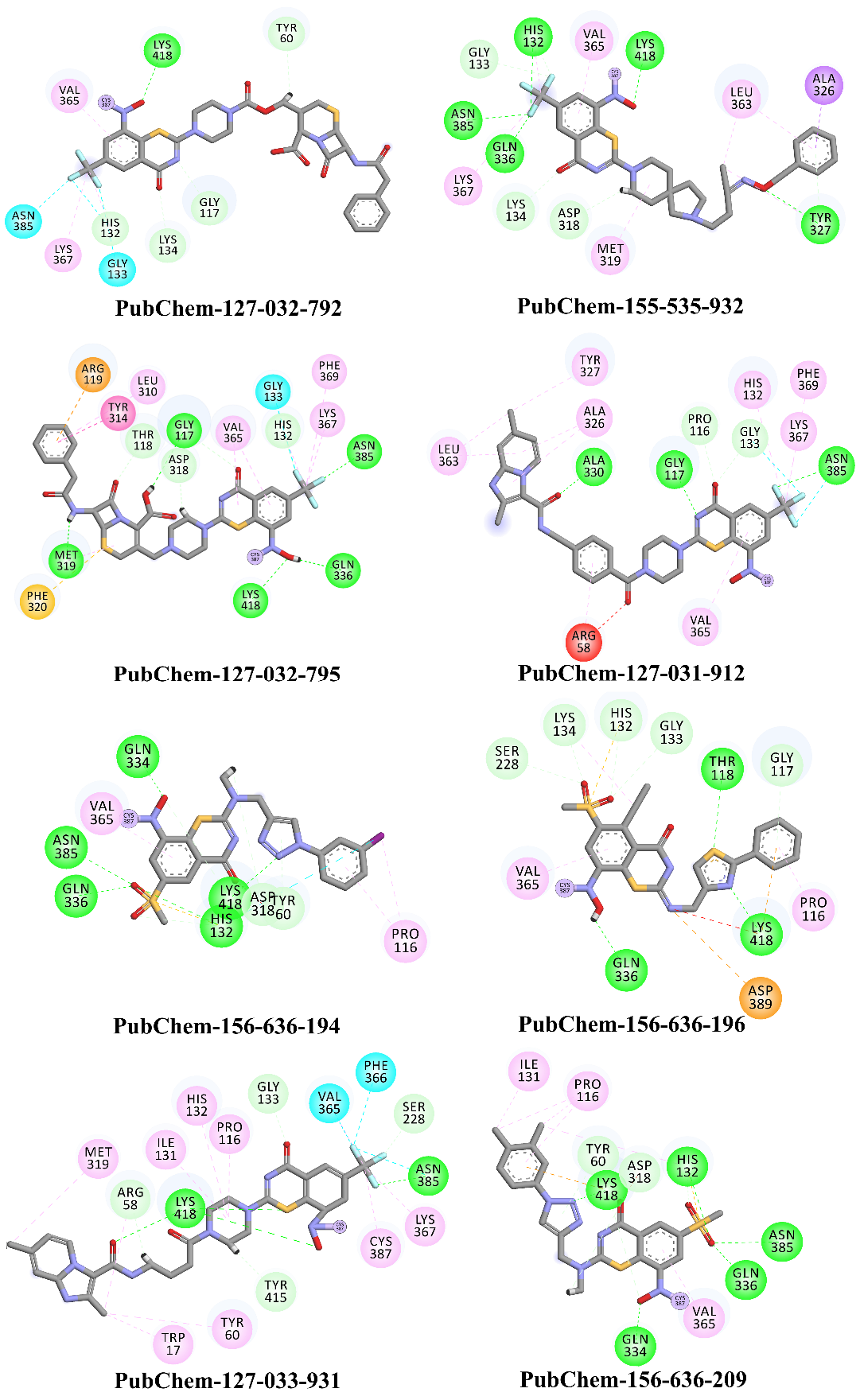


**S2 Fig**. *Continued*.

**
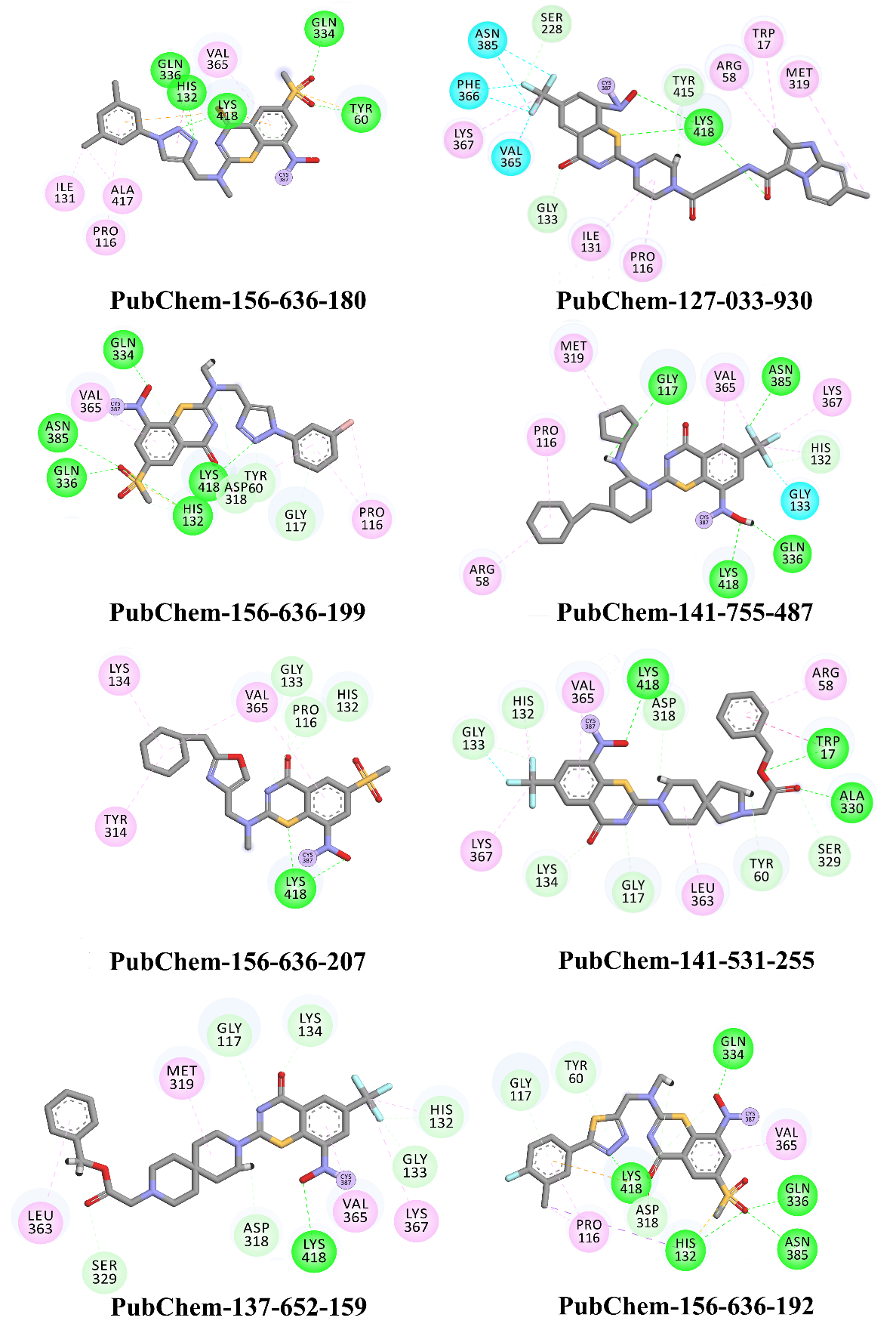
**

**S2 Fig**. *Continued*.


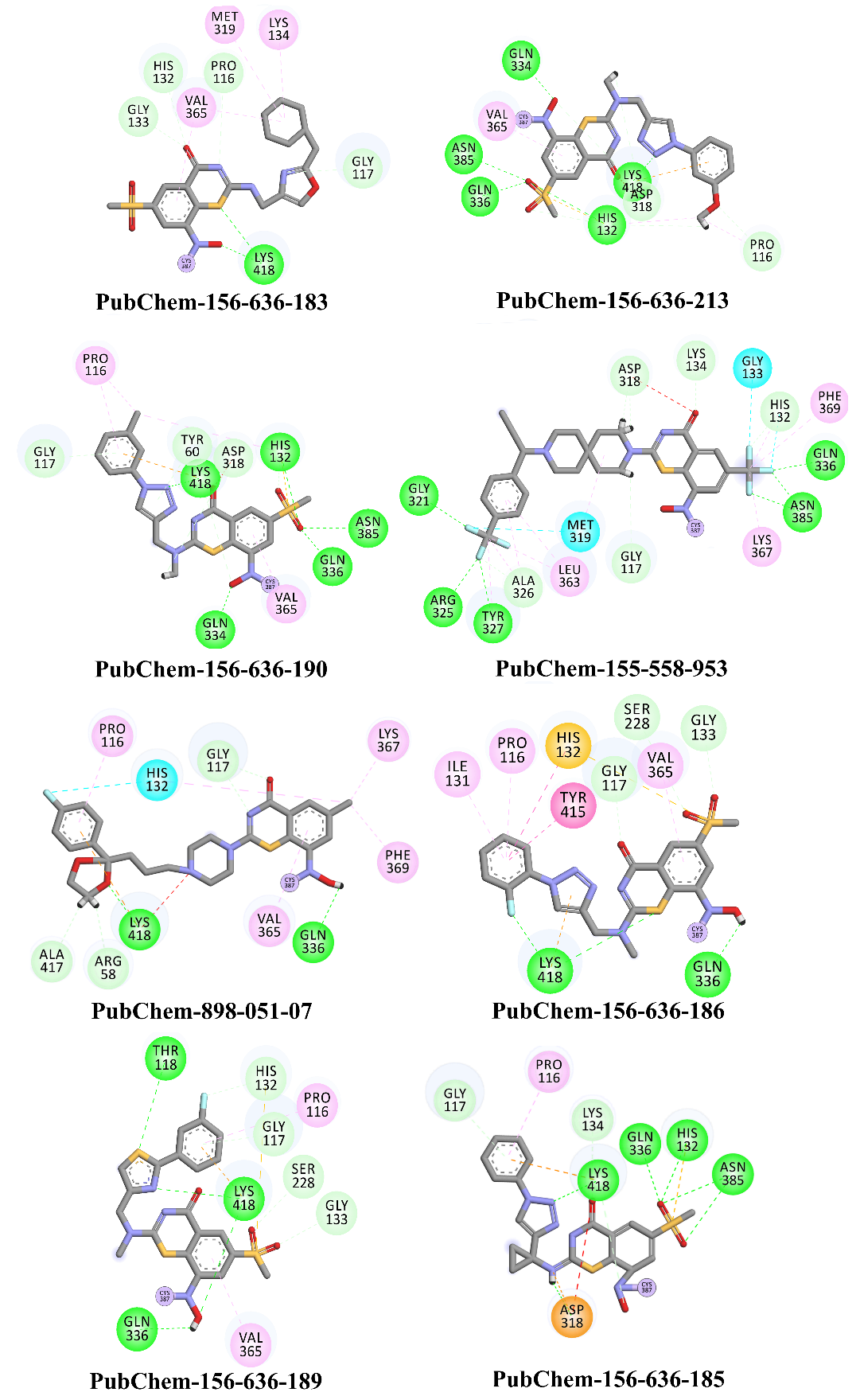


**S2 Fig**. *Continued*.


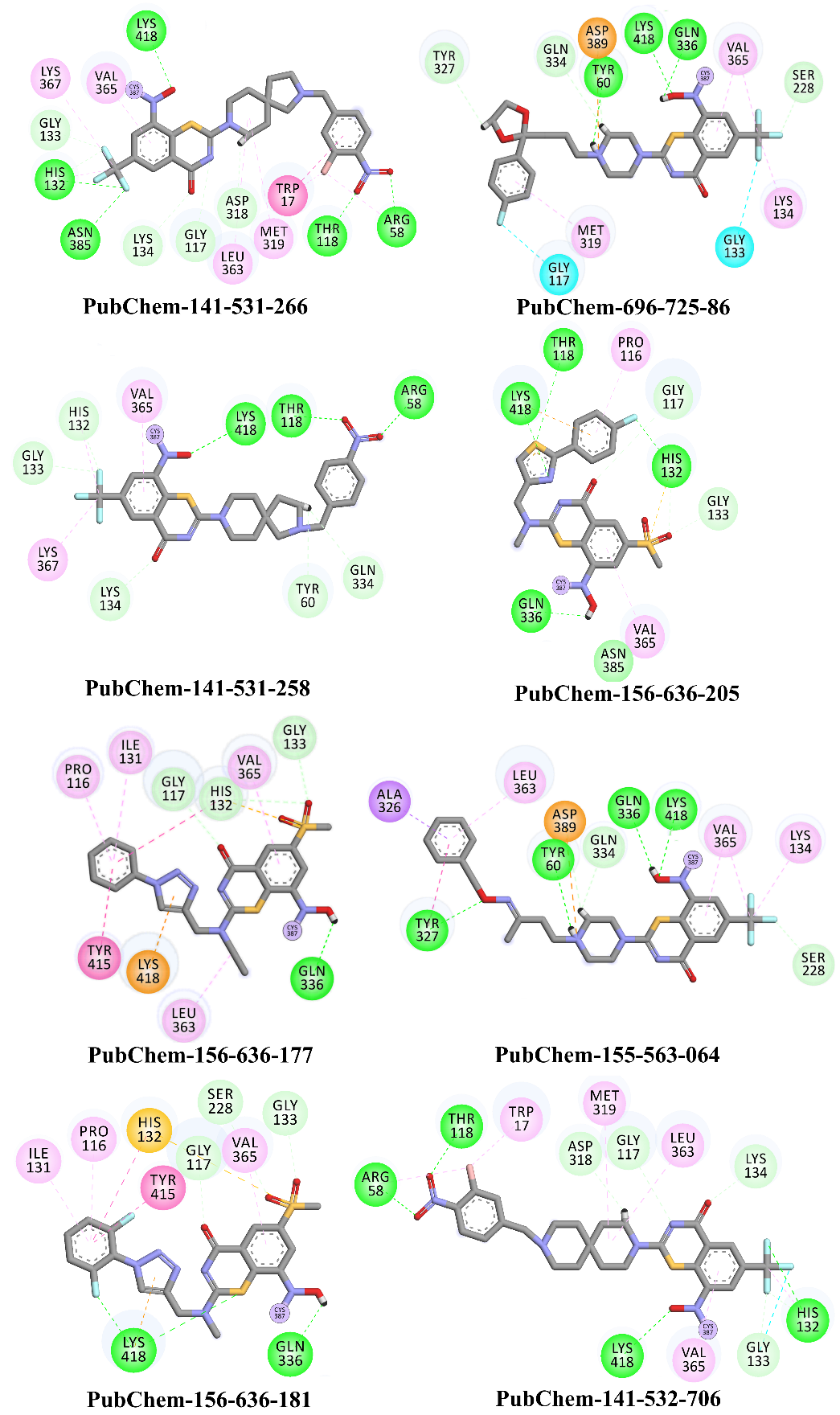


**S2 Fig**. *Continued.*


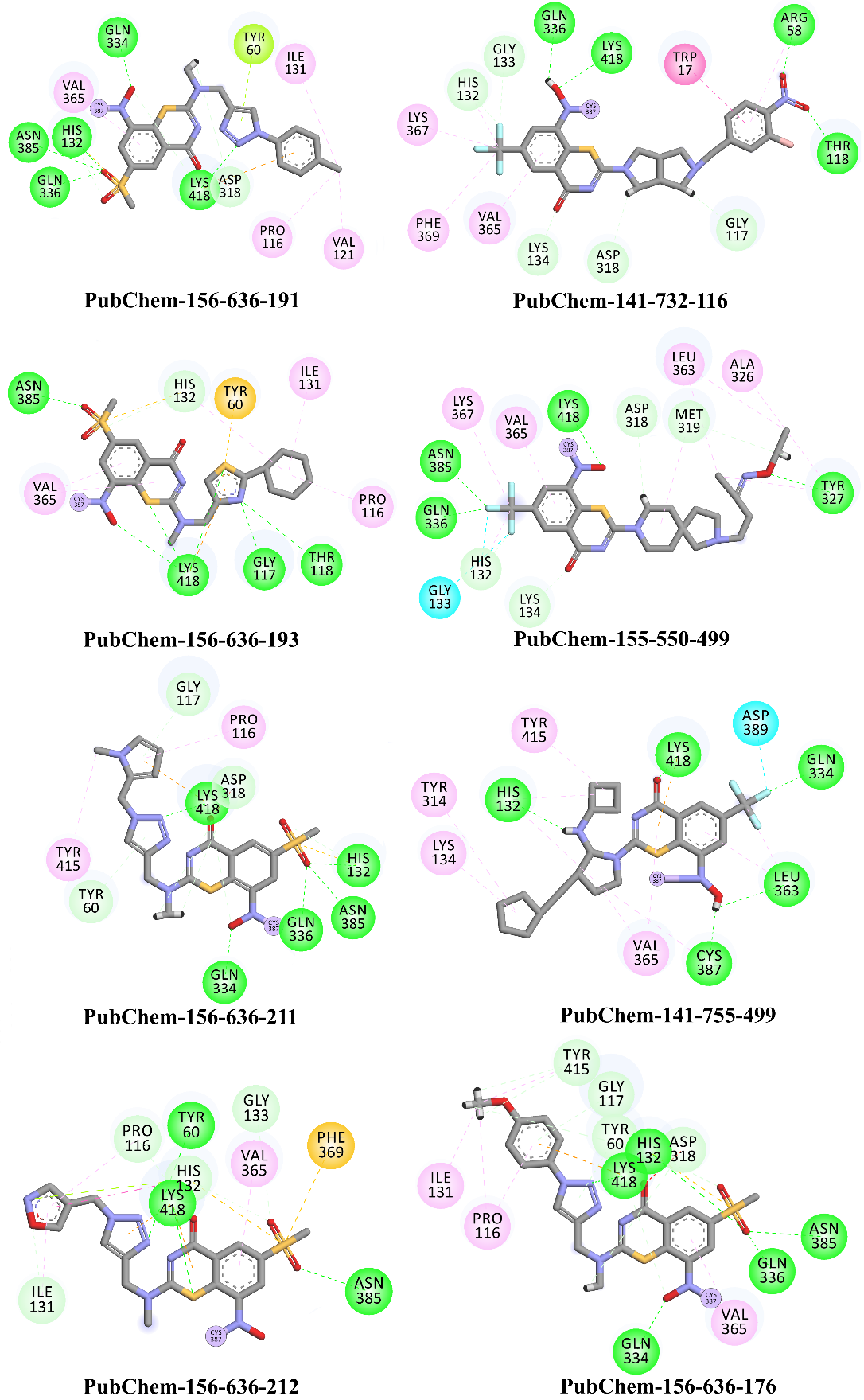


**S2 Fig**. *Continued*.

**
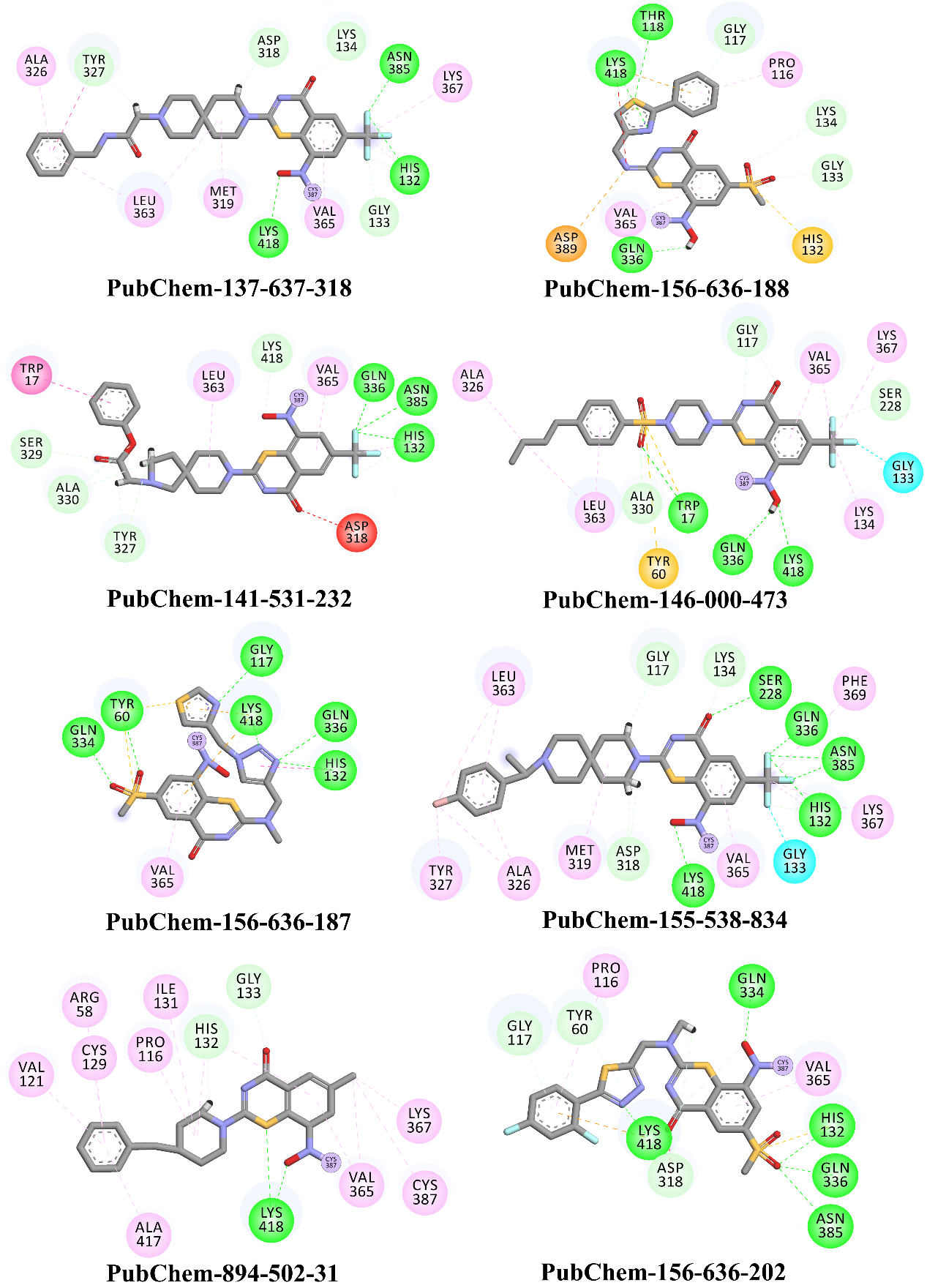
**

**S2 Fig**. *Continued.*


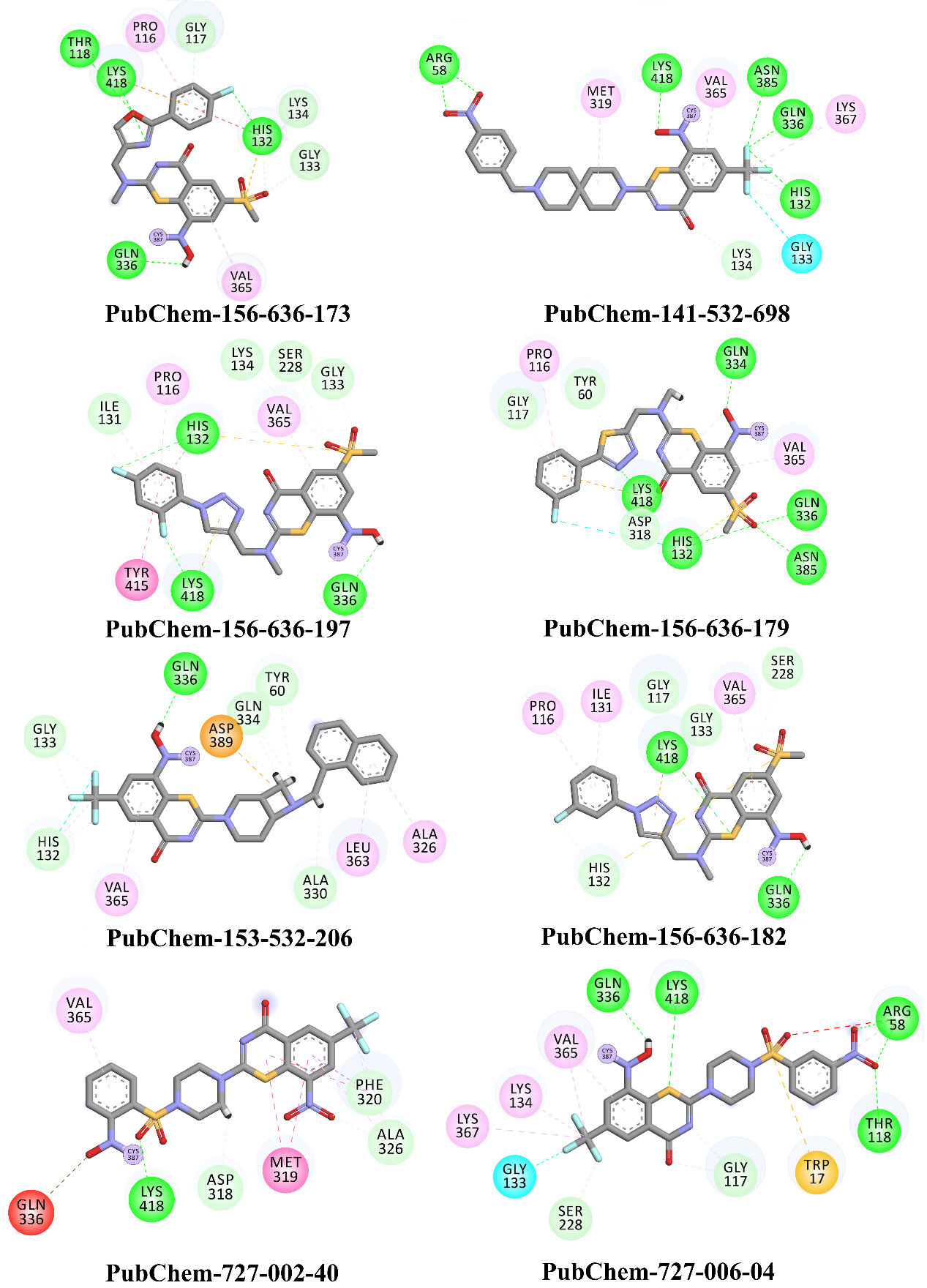


**S2 Fig**. *Continued*.


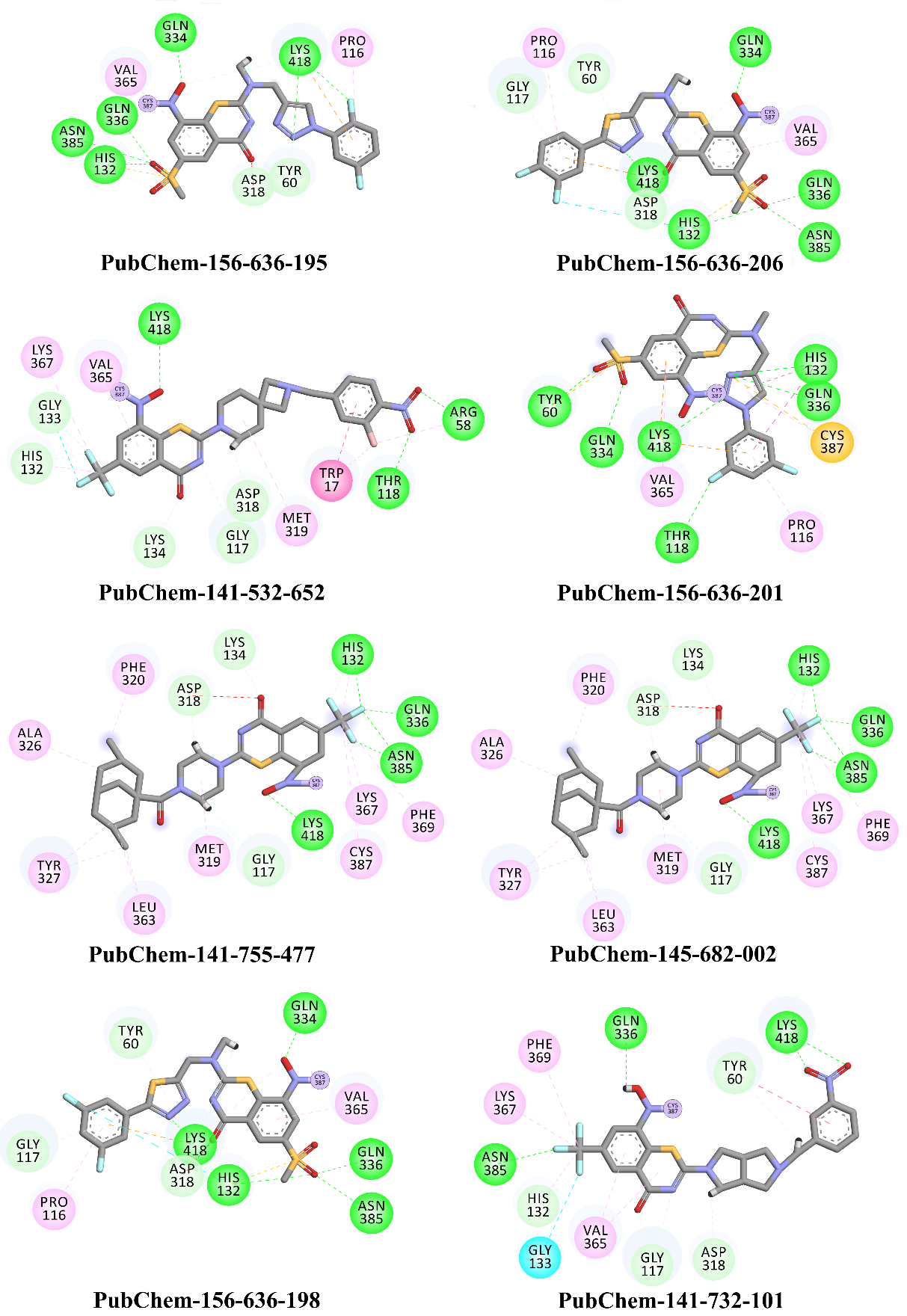


**S2 Fig**. *Continued*.


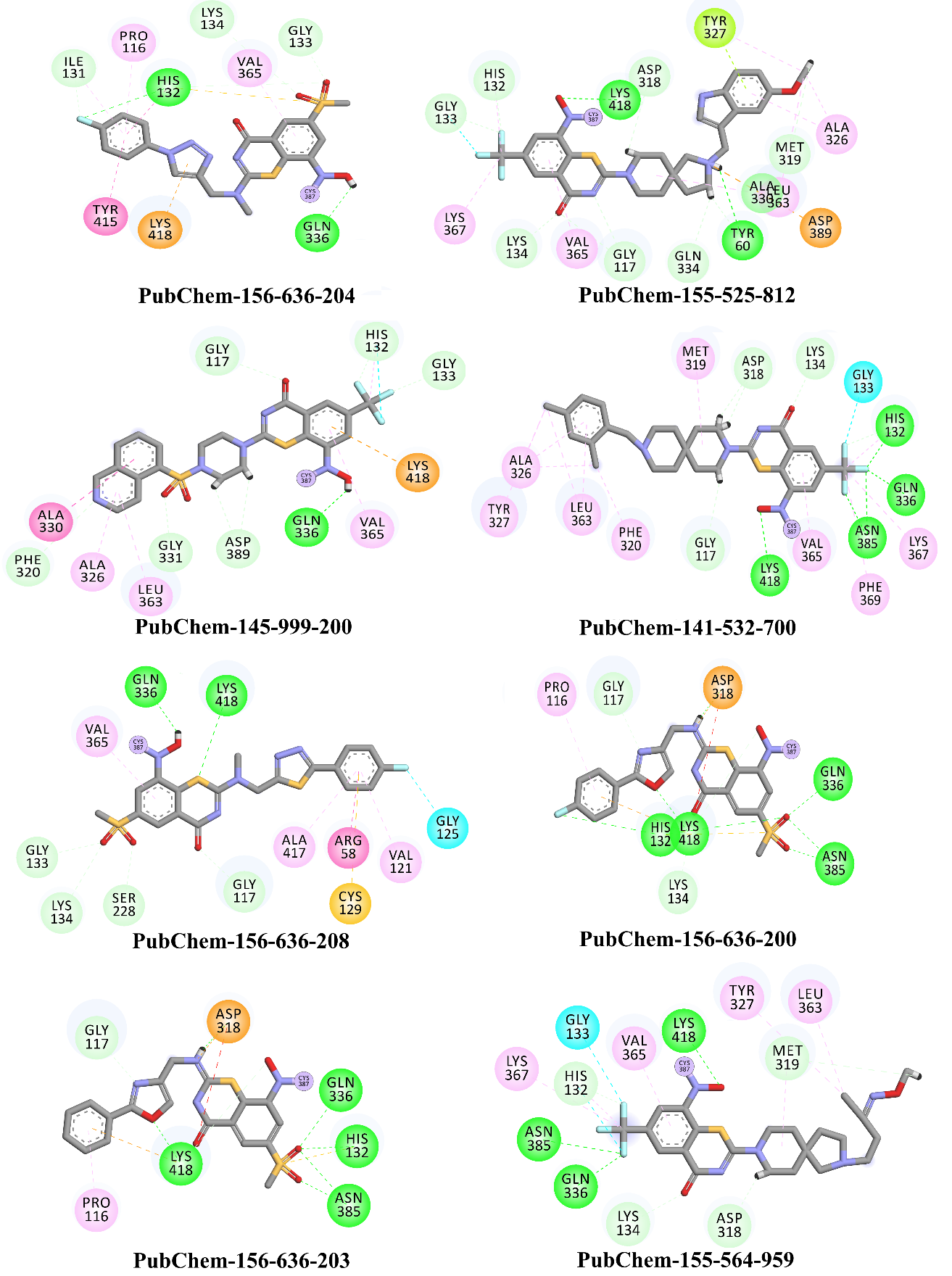


**S2 Fig**. *Continued*.

**
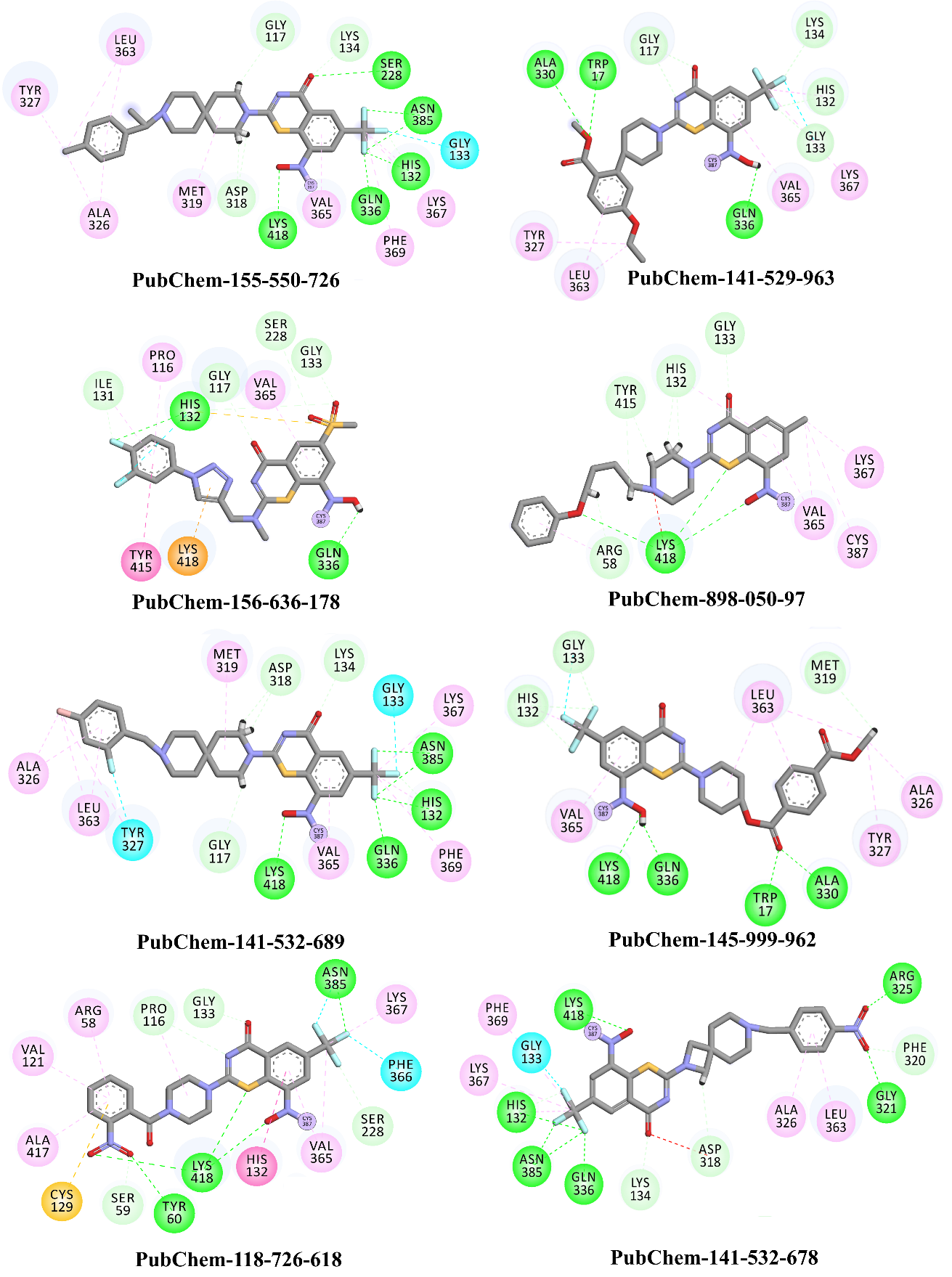
**

**S2 Fig**. *Continued*.

**
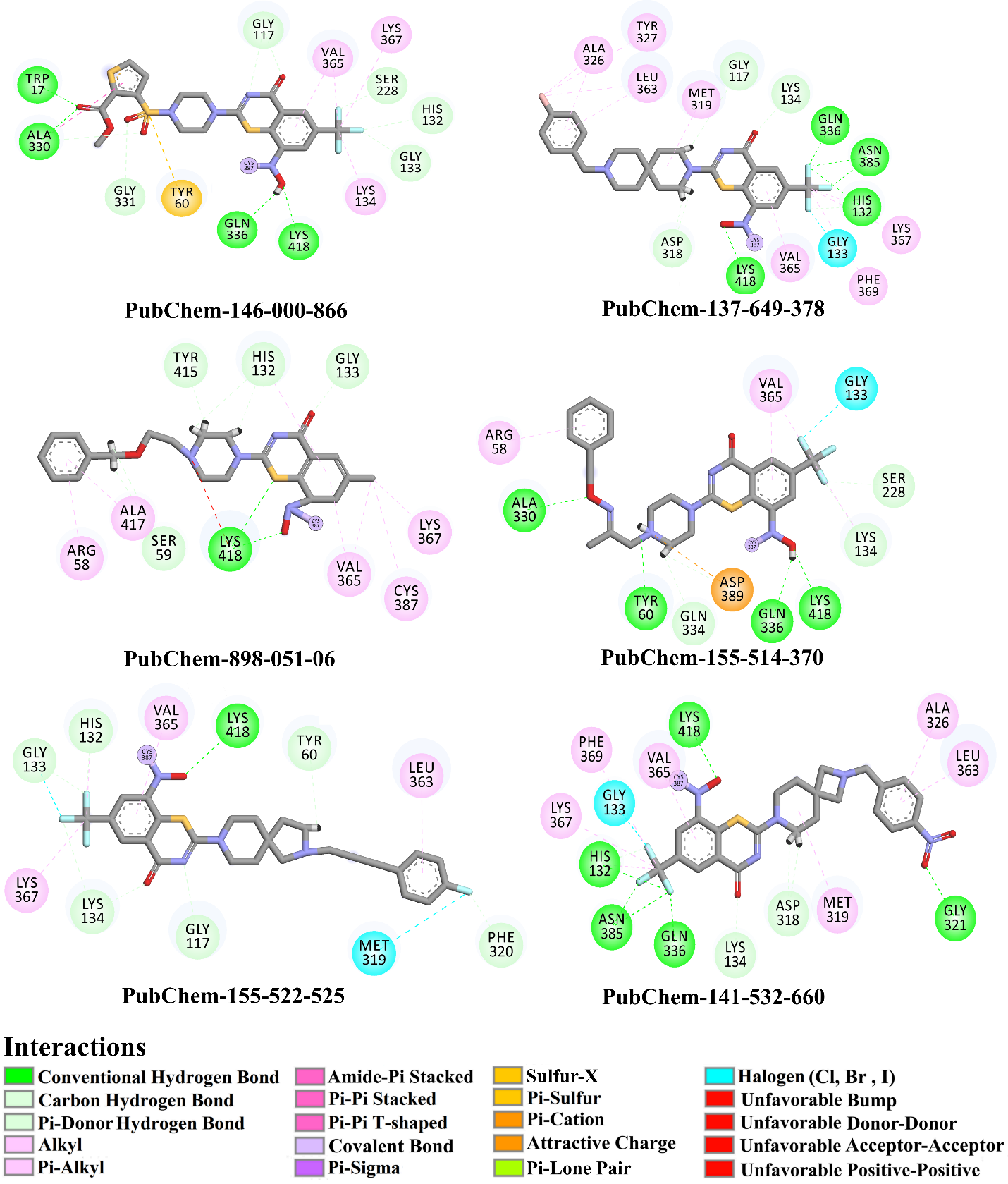
**

**S2 Fig**. *Continued*.
