# Supplementary material for: Benzothiazinone analogs as Anti-Mycobacterium tuberculosis DprE1 irreversible inhibitors: Covalent docking, validation, and molecular dynamics simulations
Source: PLoS One. 2024 Nov 25;19(11):e0314422. doi: 10.1371/journal.pone.0314422 (PMC11588222; doi:10.1371/journal.pone.0314422)
Supplement: S1 Table — (DOCX) [file pone.0314422.s003.docx]

**S1 Table**. Chemical structures, minimum inhibitory concentration (MIC) value, computed covalent docking scores, and MM-GBSA binding energies (in kcal/mol) over 100 ns MD simulations for the test set II inhibitors towards DprE1 enzyme.

| **Compound name** | **Chemical structure** | **Minimum** **inhibitory concentration (MIC) (μM)** | **Experimental binding energy (Δ*G*_exp_) (kcal/mol)** | **Covalent Docking score (kcal/mol)** | **MM-GBSA Binding Energy (kcal/mol)** |
| --- | --- | --- | --- | --- | --- |
| **PBTZ169** |  | 0.0006 | −12.6 | −7.8 | −49.8 |
| **BTZ043** |  | 0.002 | −11.9 | −7.4 | −38.7 |
| **DNB1** |  | 0.69 | −8.4 | −9.4 | −32.0 |
| **VI-9376** |  | 3.1 | −7.5 | −6.3 | −30.7 |
| **BTO** |  | 0.07 | −9.7 | −6.4 | −31.9 |
| **cBT** |  | 4.2 | −7.3 | −8.0 | −35.5 |
