# Supplementary material for: Benzothiazinone analogs as Anti-Mycobacterium tuberculosis DprE1 irreversible inhibitors: Covalent docking, validation, and molecular dynamics simulations
Source: PLoS One. 2024 Nov 25;19(11):e0314422. doi: 10.1371/journal.pone.0314422 (PMC11588222; doi:10.1371/journal.pone.0314422)
Supplement: S2 Table — (DOCX) [file pone.0314422.s004.docx]

### S2 Table. Calculated fast covalent docking scores (in kcal/mol) for PBTZ169 and the top 754 potent BTZ analogs against DprE1 active site.

| No. | PubChem Code | Covalent Docking Score (kcal/mol) | No. | PubChem Code | Covalent Docking Score (kcal/mol) | No. | PubChem Code | Covalent Docking Score (kcal/mol) |
| --- | --- | --- | --- | --- | --- | --- | --- | --- |
|  | PBTZ169 | −7.8 | 52 | PubChem-146-000-473 | −9.5 | 104 | PubChem-141-755-472 | −8.8 |
| 1 | PubChem-155-924-621 | −15.0 | 53 | PubChem-156-636-187 | −9.5 | 105 | PubChem-141-732-109 | −8.8 |
| 2 | PubChem-127-032-794 | −14.3 | 54 | PubChem-155-563-064 | −9.4 | 106 | PubChem-898-018-27 | −8.8 |
| 3 | PubChem-155-925-517 | −14.1 | 55 | PubChem-153-532-206 | −9.4 | 107 | PubChem-122-552-414 | −8.8 |
| 4 | PubChem-155-923-971 | −13.7 | 56 | PubChem-156-636-182 | −9.4 | 108 | PubChem-118-726-617 | −8.8 |
| 5 | PubChem-155-925-252 | −13.4 | 57 | PubChem-141-755-499 | −9.4 | 109 | PubChem-898-050-97 | −8.8 |
| 6 | PubChem-127-032-793 | −13.1 | 58 | PubChem-141-531-232 | −9.4 | 110 | PubChem-898-051-06 | −8.8 |
| 7 | PubChem-155-923-972 | −12.8 | 59 | PubChem-156-636-197 | −9.4 | 111 | PubChem-727-005-25 | −8.8 |
| 8 | PubChem-127-032-792 | −11.6 | 60 | PubChem-141-532-698 | −9.4 | 112 | PubChem-141-755-480 | −8.8 |
| 9 | PubChem-127-031-914 | −11.5 | 61 | PubChem-156-636-179 | −9.4 | 113 | PubChem-137-634-174 | −8.7 |
| 10 | PubChem-127-032-795 | −11.0 | 62 | PubChem-156-636-195 | −9.4 | 114 | PubChem-714-607-79 | −8.7 |
| 11 | PubChem-156-636-194 | −10.9 | 63 | PubChem-137-637-318 | −9.3 | 115 | PubChem-145-999-223 | −8.7 |
| 12 | PubChem-156-636-196 | −10.9 | 64 | PubChem-156-636-201 | −9.3 | 116 | PubChem-141-529-981 | −8.7 |
| 13 | PubChem-127-033-931 | −10.6 | 65 | PubChem-141-532-652 | −9.3 | 117 | PubChem-141-532-707 | −8.7 |
| 14 | PubChem-155-535-932 | −10.5 | 66 | PubChem-145-682-002 | −9.3 | 118 | PubChem-156-636-174 | −8.7 |
| 15 | PubChem-156-636-199 | −10.5 | 67 | PubChem-156-636-198 | −9.3 | 119 | PubChem-141-532-685 | −8.7 |
| 16 | PubChem-156-636-209 | −10.4 | 68 | PubChem-156-636-206 | −9.3 | 120 | PubChem-141-532-705 | −8.7 |
| 17 | PubChem-156-636-180 | −10.4 | 69 | PubChem-155-525-812 | −9.3 | 121 | PubChem-141-532-688 | −8.7 |
| 18 | PubChem-127-031-912 | −10.3 | 70 | PubChem-156-636-204 | −9.3 | 122 | PubChem-141-532-649 | −8.7 |
| 19 | PubChem-156-636-192 | −10.2 | 71 | PubChem-145-999-200 | −9.2 | 123 | PubChem-141-532-640 | −8.6 |
| 20 | PubChem-141-755-487 | −10.2 | 72 | PubChem-141-732-101 | −9.2 | 124 | PubChem-155-562-434 | −8.6 |
| 21 | PubChem-137-652-159 | −10.2 | 73 | PubChem-156-636-208 | −9.2 | 125 | PubChem-145-999-339 | −8.6 |
| 22 | PubChem-156-636-213 | −10.2 | 74 | PubChem-727-006-04 | −9.2 | 126 | PubChem-141-529-965 | −8.6 |
| 23 | PubChem-141-531-255 | −10.0 | 75 | PubChem-141-532-700 | −9.2 | 127 | PubChem-146-000-962 | −8.6 |
| 24 | PubChem-156-636-190 | −10.0 | 76 | PubChem-155-550-726 | −9.2 | 128 | PubChem-141-532-644 | −8.6 |
| 25 | PubChem-156-636-183 | −10.0 | 77 | PubChem-156-636-178 | −9.1 | 129 | PubChem-129-904-732 | −8.6 |
| 26 | PubChem-155-558-953 | −9.9 | 78 | PubChem-156-636-203 | −9.1 | 130 | PubChem-141-532-690 | −8.6 |
| 27 | PubChem-156-636-186 | −9.9 | 79 | PubChem-155-564-959 | −9.1 | 131 | PubChem-141-532-646 | −8.6 |
| 28 | PubChem-156-636-189 | −9.9 | 80 | PubChem-141-529-963 | −9.1 | 132 | PubChem-155-541-557 | −8.6 |
| 29 | PubChem-156-636-207 | −9.9 | 81 | PubChem-141-532-689 | −9.1 | 133 | PubChem-141-755-475 | −8.6 |
| 30 | PubChem-141-531-266 | −9.8 | 82 | PubChem-156-636-200 | −9.1 | 134 | PubChem-145-984-191 | −8.6 |
| 31 | PubChem-156-636-205 | −9.8 | 83 | PubChem-118-726-618 | −9.1 | 135 | PubChem-137-656-146 | −8.6 |
| 32 | PubChem-156-636-177 | −9.8 | 84 | PubChem-146-000-866 | −9.0 | 136 | PubChem-141-532-686 | −8.6 |
| 33 | PubChem-141-531-258 | −9.8 | 85 | PubChem-137-649-378 | −9.0 | 137 | PubChem-141-532-680 | −8.6 |
| 34 | PubChem-898-051-07 | −9.8 | 86 | PubChem-727-002-40 | −9.0 | 138 | PubChem-156-636-210 | −8.6 |
| 35 | PubChem-156-636-181 | −9.8 | 87 | PubChem-145-999-962 | −9.0 | 139 | PubChem-137-644-321 | −8.6 |
| 36 | PubChem-141-532-706 | −9.8 | 88 | PubChem-155-522-525 | −9.0 | 140 | PubChem-145-998-587 | −8.5 |
| 37 | PubChem-127-033-930 | −9.8 | 89 | PubChem-127-034-462 | −9.0 | 141 | PubChem-141-532-693 | −8.5 |
| 38 | PubChem-156-636-191 | −9.8 | 90 | PubChem-141-532-678 | −9.0 | 142 | PubChem-727-002-39 | −8.5 |
| 39 | PubChem-696-725-86 | −9.7 | 91 | PubChem-141-532-660 | −9.0 | 143 | PubChem-161-854-875 | −8.5 |
| 40 | PubChem-156-636-193 | −9.7 | 92 | PubChem-156-636-184 | −9.0 | 144 | PubChem-573-881-13 | −8.5 |
| 41 | PubChem-156-636-185 | −9.7 | 93 | PubChem-141-532-692 | −8.9 | 145 | PubChem-875-023-69 | −8.5 |
| 42 | PubChem-141-732-116 | −9.7 | 94 | PubChem-155-547-100 | −8.9 | 146 | PubChem-898-020-15 | −8.5 |
| 43 | PubChem-156-636-211 | −9.7 | 95 | PubChem-155-540-811 | −8.9 | 147 | PubChem-141-532-704 | −8.5 |
| 44 | PubChem-155-550-499 | −9.6 | 96 | PubChem-145-999-092 | −8.9 | 148 | PubChem-898-051-03 | −8.5 |
| 45 | PubChem-156-636-176 | −9.6 | 97 | PubChem-141-755-482 | −8.9 | 149 | PubChem-155-514-370 | −8.5 |
| 46 | PubChem-156-636-188 | −9.6 | 98 | PubChem-573-878-81 | −8.9 | 150 | PubChem-142-758-240 | −8.5 |
| 47 | PubChem-156-636-212 | −9.6 | 99 | PubChem-156-703-637 | −8.8 | 151 | PubChem-696-761-86 | −8.5 |
| 48 | PubChem-894-502-31 | −9.5 | 100 | PubChem-727-004-32 | −8.8 | 152 | PubChem-573-878-80 | −8.5 |
| 49 | PubChem-156-636-202 | −9.5 | 101 | PubChem-141-531-241 | −8.8 | 153 | PubChem-137-653-833 | −8.5 |
| 50 | PubChem-155-538-834 | −9.5 | 102 | PubChem-137-632-150 | −8.8 | 154 | PubChem-156-776-528 | −8.5 |
| 51 | PubChem-156-636-173 | −9.5 | 103 | PubChem-155-568-069 | −8.8 | 155 | PubChem-573-878-82 | −8.5 |

### S2 Table. *Continued*.

| No. | PubChem Code | Covalent Docking Score (kcal/mol) | No. | PubChem Code | Covalent Docking Score (kcal/mol) | No. | PubChem Code | Covalent Docking Score (kcal/mol) |
| --- | --- | --- | --- | --- | --- | --- | --- | --- |
| 156 | PubChem-118-726-621 | −8.5 | 208 | PubChem-573-881-14 | −8.2 | 260 | PubChem-141-531-239 | −8.0 |
| 157 | PubChem-141-532-696 | −8.4 | 209 | PubChem-141-532-697 | −8.2 | 261 | PubChem-141-531-237 | −8.0 |
| 158 | PubChem-146-000-472 | −8.4 | 210 | PubChem-137-657-258 | −8.2 | 262 | PubChem-141-732-083 | −8.0 |
| 159 | PubChem-141-531-248 | −8.4 | 211 | PubChem-146-000-011 | −8.2 | 263 | PubChem-141-732-081 | −8.0 |
| 160 | PubChem-118-726-620 | −8.4 | 212 | PubChem-141-732-095 | −8.2 | 264 | PubChem-141-531-260 | −8.0 |
| 161 | PubChem-141-755-496 | −8.4 | 213 | PubChem-118-726-632 | −8.2 | 265 | PubChem-137-641-460 | −8.0 |
| 162 | PubChem-141-532-684 | −8.4 | 214 | PubChem-158-904-351 | −8.2 | 266 | PubChem-141-531-252 | −8.0 |
| 163 | PubChem-145-999-091 | −8.4 | 215 | PubChem-118-570-041 | −8.2 | 267 | PubChem-156-776-527 | −8.0 |
| 164 | PubChem-146-000-167 | −8.4 | 216 | PubChem-141-531-242 | −8.2 | 268 | PubChem-141-732-114 | −8.0 |
| 165 | PubChem-141-532-638 | −8.4 | 217 | PubChem-141-755-494 | −8.2 | 269 | PubChem-134-372-729 | −8.0 |
| 166 | PubChem-727-004-31 | −8.4 | 218 | PubChem-727-004-33 | −8.2 | 270 | PubChem-156-703-636 | −8.0 |
| 167 | PubChem-155-538-457 | −8.4 | 219 | PubChem-696-761-85 | −8.2 | 271 | PubChem-156-703-649 | −8.0 |
| 168 | PubChem-141-532-639 | −8.4 | 220 | PubChem-122-552-420 | −8.2 | 272 | PubChem-141-532-648 | −8.0 |
| 169 | PubChem-141-532-651 | −8.4 | 221 | PubChem-727-004-34 | −8.2 | 273 | PubChem-141-531-243 | −8.0 |
| 170 | PubChem-141-755-474 | −8.4 | 222 | PubChem-141-732-104 | −8.2 | 274 | PubChem-141-532-664 | −8.0 |
| 171 | PubChem-141-532-653 | −8.4 | 223 | PubChem-141-531-263 | −8.2 | 275 | PubChem-141-532-675 | −8.0 |
| 172 | PubChem-137-651-027 | −8.4 | 224 | PubChem-714-607-78 | −8.2 | 276 | PubChem-156-703-651 | −8.0 |
| 173 | PubChem-155-435-068 | −8.4 | 225 | PubChem-156-703-653 | −8.2 | 277 | PubChem-137-646-426 | −8.0 |
| 174 | PubChem-146-000-368 | −8.4 | 226 | PubChem-155-557-174 | −8.2 | 278 | PubChem-118-712-952 | −8.0 |
| 175 | PubChem-155-512-871 | −8.4 | 227 | PubChem-141-532-663 | −8.2 | 279 | PubChem-141-532-654 | −8.0 |
| 176 | PubChem-898-051-08 | −8.4 | 228 | PubChem-141-532-667 | −8.1 | 280 | PubChem-141-755-492 | −8.0 |
| 177 | PubChem-141-532-694 | −8.4 | 229 | PubChem-141-532-658 | −8.1 | 281 | PubChem-898-050-88 | −8.0 |
| 178 | PubChem-155-568-053 | −8.4 | 230 | PubChem-141-732-088 | −8.1 | 282 | PubChem-122-552-401 | −8.0 |
| 179 | PubChem-141-755-477 | −8.3 | 231 | PubChem-144-404-685 | −8.1 | 283 | PubChem-122-552-400 | −8.0 |
| 180 | PubChem-146-000-697 | −8.3 | 232 | PubChem-141-532-671 | −8.1 | 284 | PubChem-155-551-745 | −8.0 |
| 181 | PubChem-898-050-95 | −8.3 | 233 | PubChem-141-531-244 | −8.1 | 285 | PubChem-146-000-203 | −8.0 |
| 182 | PubChem-141-532-647 | −8.3 | 234 | PubChem-137-656-990 | −8.1 | 286 | PubChem-141-532-673 | −8.0 |
| 183 | PubChem-141-532-645 | −8.3 | 235 | PubChem-715-164-17 | −8.1 | 287 | PubChem-141-532-655 | −8.0 |
| 184 | PubChem-898-050-91 | −8.3 | 236 | PubChem-727-003-36 | −8.1 | 288 | PubChem-145-998-704 | −7.9 |
| 185 | PubChem-161-180-922 | −8.3 | 237 | PubChem-141-755-484 | −8.1 | 289 | PubChem-146-000-168 | −7.9 |
| 186 | PubChem-141-532-701 | −8.3 | 238 | PubChem-141-532-677 | −8.1 | 290 | PubChem-141-732-102 | −7.9 |
| 187 | PubChem-141-532-643 | −8.3 | 239 | PubChem-155-533-314 | −8.1 | 291 | PubChem-898-022-46 | −7.9 |
| 188 | PubChem-141-531-247 | −8.3 | 240 | PubChem-141-532-708 | −8.1 | 292 | PubChem-141-732-117 | −7.9 |
| 189 | PubChem-141-531-230 | −8.3 | 241 | PubChem-141-532-665 | −8.1 | 293 | PubChem-141-532-709 | −7.9 |
| 190 | PubChem-137-661-096 | −8.3 | 242 | PubChem-141-532-681 | −8.1 | 294 | PubChem-715-006-96 | −7.9 |
| 191 | PubChem-141-531-236 | −8.3 | 243 | PubChem-156-703-646 | −8.1 | 295 | PubChem-141-531-259 | −7.9 |
| 192 | PubChem-674-271-15 | −8.3 | 244 | PubChem-146-000-658 | −8.1 | 296 | PubChem-141-531-250 | −7.9 |
| 193 | PubChem-118-726-627 | −8.3 | 245 | PubChem-141-532-676 | −8.1 | 297 | PubChem-160-162-190 | −7.9 |
| 194 | PubChem-141-531-235 | −8.3 | 246 | PubChem-898-051-04 | −8.1 | 298 | PubChem-142-308-757 | −7.9 |
| 195 | PubChem-145-998-586 | −8.3 | 247 | PubChem-898-020-56 | −8.1 | 299 | PubChem-141-732-111 | −7.9 |
| 196 | PubChem-145-999-885 | −8.3 | 248 | PubChem-141-531-240 | −8.1 | 300 | PubChem-141-732-089 | −7.9 |
| 197 | PubChem-145-998-585 | −8.3 | 249 | PubChem-145-984-054 | −8.1 | 301 | PubChem-137-633-247 | −7.9 |
| 198 | PubChem-146-001-133 | −8.3 | 250 | PubChem-141-732-084 | −8.1 | 302 | PubChem-134-372-749 | −7.9 |
| 199 | PubChem-141-531-262 | −8.3 | 251 | PubChem-727-001-56 | −8.0 | 303 | PubChem-141-755-489 | −7.9 |
| 200 | PubChem-141-755-485 | −8.3 | 252 | PubChem-122-552-410 | −8.0 | 304 | PubChem-155-542-479 | −7.9 |
| 201 | PubChem-248-300-94 | −8.3 | 253 | PubChem-122-552-409 | −8.0 | 305 | PubChem-134-262-692 | −7.9 |
| 202 | PubChem-141-531-251 | −8.3 | 254 | PubChem-723-750-17 | −8.0 | 306 | PubChem-122-552-402 | −7.9 |
| 203 | PubChem-739-471-95 | −8.3 | 255 | PubChem-118-726-628 | −8.0 | 307 | PubChem-137-659-885 | −7.9 |
| 204 | PubChem-134-366-636 | −8.2 | 256 | PubChem-573-881-15 | −8.0 | 308 | PubChem-141-732-110 | −7.9 |
| 205 | PubChem-141-532-662 | −8.2 | 257 | PubChem-155-533-696 | −8.0 | 309 | PubChem-141-531-234 | −7.9 |
| 206 | PubChem-141-531-253 | −8.2 | 258 | PubChem-141-532-679 | −8.0 | 310 | PubChem-118-726-633 | −7.9 |
| 207 | PubChem-727-003-35 | −8.2 | 259 | PubChem-144-404-686 | −8.0 | 311 | PubChem-118-726-626 | −7.9 |

## S2 Table. Continued.

| No. | PubChem Code | Covalent Docking Score (kcal/mol) | No. | PubChem Code | Covalent Docking Score (kcal/mol) | No. | PubChem Code | Covalent Docking Score (kcal/mol) |
| --- | --- | --- | --- | --- | --- | --- | --- | --- |
| 312 | PubChem-674-276-33 | −7.9 | 364 | PubChem-141-531-249 | −7.7 | 416 | PubChem-715-163-51 | −7.5 |
| 313 | PubChem-898-022-85 | −7.9 | 365 | PubChem-141-532-637 | −7.7 | 417 | PubChem-146-000-908 | −7.5 |
| 314 | PubChem-894-502-30 | −7.9 | 366 | PubChem-715-164-88 | −7.7 | 418 | PubChem-145-984-115 | −7.5 |
| 315 | PubChem-141-531-256 | −7.9 | 367 | PubChem-141-732-093 | −7.7 | 419 | PubChem-137-637-768 | −7.5 |
| 316 | PubChem-145-998-336 | −7.8 | 368 | PubChem-145-998-334 | −7.7 | 420 | PubChem-141-532-672 | −7.5 |
| 317 | PubChem-141-532-691 | −7.8 | 369 | PubChem-145-998-335 | −7.7 | 421 | PubChem-684-449-30 | −7.5 |
| 318 | PubChem-137-655-686 | −7.8 | 370 | PubChem-251-383-28 | −7.7 | 422 | PubChem-141-732-106 | −7.5 |
| 319 | PubChem-141-532-683 | −7.8 | 371 | PubChem-141-531-233 | −7.7 | 423 | PubChem-141-531-268 | −7.5 |
| 320 | PubChem-118-726-622 | −7.8 | 372 | PubChem-141-532-669 | −7.6 | 424 | PubChem-141-532-668 | −7.5 |
| 321 | PubChem-156-703-641 | −7.8 | 373 | PubChem-141-531-238 | −7.6 | 425 | PubChem-141-532-703 | −7.5 |
| 322 | PubChem-145-982-981 | −7.8 | 374 | PubChem-156-703-657 | −7.6 | 426 | PubChem-155-561-676 | −7.5 |
| 323 | PubChem-141-732-091 | −7.8 | 375 | PubChem-739-471-94 | −7.6 | 427 | PubChem-141-532-642 | −7.5 |
| 324 | PubChem-137-639-860 | −7.8 | 376 | PubChem-426-459-41 | −7.6 | 428 | PubChem-141-732-113 | −7.5 |
| 325 | PubChem-137-635-021 | −7.8 | 377 | PubChem-141-532-670 | −7.6 | 429 | PubChem-141-755-486 | −7.5 |
| 326 | PubChem-141-532-699 | −7.8 | 378 | PubChem-573-881-16 | −7.6 | 430 | PubChem-155-534-305 | −7.5 |
| 327 | PubChem-145-985-702 | −7.8 | 379 | PubChem-715-012-06 | −7.6 | 431 | PubChem-137-659-315 | −7.5 |
| 328 | PubChem-141-732-094 | −7.8 | 380 | PubChem-141-532-661 | −7.6 | 432 | PubChem-898-050-98 | −7.5 |
| 329 | PubChem-141-532-682 | −7.8 | 381 | PubChem-155-527-213 | −7.6 | 433 | PubChem-141-532-659 | −7.5 |
| 330 | PubChem-137-657-350 | −7.8 | 382 | PubChem-714-571-12 | −7.6 | 434 | PubChem-739-472-03 | −7.5 |
| 331 | PubChem-141-532-702 | −7.8 | 383 | PubChem-141-732-099 | −7.6 | 435 | PubChem-715-008-65 | −7.4 |
| 332 | PubChem-155-708-694 | −7.8 | 384 | PubChem-141-732-100 | −7.6 | 436 | PubChem-141-531-245 | −7.4 |
| 333 | PubChem-155-708-693 | −7.8 | 385 | PubChem-141-531-267 | −7.6 | 437 | PubChem-141-529-334 | −7.4 |
| 334 | PubChem-118-726-629 | −7.8 | 386 | PubChem-715-164-13 | −7.6 | 438 | PubChem-141-529-349 | −7.4 |
| 335 | PubChem-739-471-97 | −7.8 | 387 | PubChem-146-000-411 | −7.6 | 439 | PubChem-146-001-056 | −7.4 |
| 336 | PubChem-573-881-12 | −7.8 | 388 | PubChem-714-735-88 | −7.6 | 440 | PubChem-155-527-741 | −7.4 |
| 337 | PubChem-715-013-74 | −7.8 | 389 | PubChem-118-726-624 | −7.6 | 441 | PubChem-141-532-666 | −7.4 |
| 338 | PubChem-727-002-38 | −7.8 | 390 | PubChem-155-510-954 | −7.6 | 442 | PubChem-155-510-586 | −7.4 |
| 339 | PubChem-141-532-687 | −7.8 | 391 | PubChem-141-732-090 | −7.6 | 443 | PubChem-715-164-89 | −7.4 |
| 340 | PubChem-141-732-087 | −7.8 | 392 | PubChem-145-998-602 | −7.6 | 444 | PubChem-156-703-644 | −7.4 |
| 341 | PubChem-137-657-434 | −7.8 | 393 | PubChem-155-537-827 | −7.6 | 445 | PubChem-141-755-488 | −7.4 |
| 342 | PubChem-141-732-107 | −7.8 | 394 | PubChem-118-726-630 | −7.6 | 446 | PubChem-141-531-254 | −7.4 |
| 343 | PubChem-727-001-55 | −7.8 | 395 | PubChem-122-552-419 | −7.6 | 447 | PubChem-137-636-844 | −7.4 |
| 344 | PubChem-141-532-695 | −7.8 | 396 | PubChem-141-532-674 | −7.6 | 448 | PubChem-141-192-625 | −7.4 |
| 345 | PubChem-141-529-985 | −7.8 | 397 | PubChem-898-019-23 | −7.6 | 449 | PubChem-739-472-01 | −7.4 |
| 346 | PubChem-426-461-86 | −7.8 | 398 | PubChem-141-529-960 | −7.6 | 450 | PubChem-145-998-328 | −7.4 |
| 347 | PubChem-248-302-60 | −7.8 | 399 | PubChem-118-726-623 | −7.6 | 451 | PubChem-155-561-850 | −7.4 |
| 348 | PubChem-141-531-264 | −7.8 | 400 | PubChem-141-532-657 | −7.5 | 452 | PubChem-141-732-092 | −7.4 |
| 349 | PubChem-102-004-363 | −7.8 | 401 | PubChem-155-548-369 | −7.5 | 453 | PubChem-145-999-892 | −7.4 |
| 350 | PubChem-141-531-261 | −7.7 | 402 | PubChem-714-625-06 | −7.5 | 454 | PubChem-155-532-293 | −7.4 |
| 351 | PubChem-156-703-640 | −7.7 | 403 | PubChem-718-191-23 | −7.5 | 455 | PubChem-141-529-966 | −7.4 |
| 352 | PubChem-155-546-535 | −7.7 | 404 | PubChem-141-732-096 | −7.5 | 456 | PubChem-155-532-417 | −7.4 |
| 353 | PubChem-141-755-481 | −7.7 | 405 | PubChem-141-732-108 | −7.5 | 457 | PubChem-141-531-265 | −7.4 |
| 354 | PubChem-727-005-21 | −7.7 | 406 | PubChem-141-531-246 | −7.5 | 458 | PubChem-155-518-376 | −7.4 |
| 355 | PubChem-141-755-491 | −7.7 | 407 | PubChem-141-532-641 | −7.5 | 459 | PubChem-155-538-207 | −7.4 |
| 356 | PubChem-118-726-637 | −7.7 | 408 | PubChem-155-516-987 | −7.5 | 460 | PubChem-714-589-82 | −7.4 |
| 357 | PubChem-155-547-692 | −7.7 | 409 | PubChem-156-703-655 | −7.5 | 461 | PubChem-715-012-07 | −7.4 |
| 358 | PubChem-155-562-623 | −7.7 | 410 | PubChem-671-490-34 | −7.5 | 462 | PubChem-715-013-73 | −7.4 |
| 359 | PubChem-248-300-99 | −7.7 | 411 | PubChem-739-471-98 | −7.5 | 463 | PubChem-155-521-453 | −7.3 |
| 360 | PubChem-739-471-99 | −7.7 | 412 | PubChem-898-050-96 | −7.5 | 464 | PubChem-715-164-16 | −7.3 |
| 361 | PubChem-141-732-118 | −7.7 | 413 | PubChem-154-361-008 | −7.5 | 465 | PubChem-118-557-786 | −7.3 |
| 362 | PubChem-118-726-625 | −7.7 | 414 | PubChem-248-300-98 | −7.5 | 466 | PubChem-141-755-479 | −7.3 |
| 363 | PubChem-141-531-231 | −7.7 | 415 | PubChem-714-607-81 | −7.5 | 467 | PubChem-141-529-979 | −7.3 |

## S2 Table. Continued.

| No. | PubChem Code | Covalent Docking Score (kcal/mol) | No. | PubChem Code | Covalent Docking Score (kcal/mol) | No. | PubChem Code | Covalent Docking Score (kcal/mol) |
| --- | --- | --- | --- | --- | --- | --- | --- | --- |
| 468 | PubChem-155-538-275 | −7.3 | 520 | PubChem-122-552-416 | −7.1 | 572 | PubChem-426-459-40 | −6.8 |
| 469 | PubChem-155-556-180 | −7.3 | 521 | PubChem-155-528-256 | −7.1 | 573 | PubChem-714-536-11 | −6.8 |
| 470 | PubChem-156-703-639 | −7.3 | 522 | PubChem-122-552-417 | −7.1 | 574 | PubChem-898-021-47 | −6.8 |
| 471 | PubChem-696-763-96 | −7.3 | 523 | PubChem-122-552-418 | −7.1 | 575 | PubChem-122-552-405 | −6.8 |
| 472 | PubChem-155-708-698 | −7.3 | 524 | PubChem-141-732-098 | −7.1 | 576 | PubChem-122-552-406 | −6.8 |
| 473 | PubChem-155-708-699 | −7.3 | 525 | PubChem-141-732-103 | −7.1 | 577 | PubChem-898-050-89 | −6.8 |
| 474 | PubChem-727-003-39 | −7.3 | 526 | PubChem-684-285-83 | −7.1 | 578 | PubChem-141-755-495 | −6.8 |
| 475 | PubChem-248-300-97 | −7.3 | 527 | PubChem-141-529-969 | −7.1 | 579 | PubChem-153-947-707 | −6.8 |
| 476 | PubChem-141-532-656 | −7.3 | 528 | PubChem-145-999-884 | −7.1 | 580 | PubChem-156-703-642 | −6.8 |
| 477 | PubChem-684-294-51 | −7.3 | 529 | PubChem-141-529-975 | −7.1 | 581 | PubChem-158-904-351 | −6.8 |
| 478 | PubChem-573-878-83 | −7.3 | 530 | PubChem-156-703-658 | −7.0 | 582 | PubChem-718-189-83 | −6.8 |
| 479 | PubChem-145-998-627 | −7.3 | 531 | PubChem-155-708-702 | −7.0 | 583 | PubChem-145-999-788 | −6.7 |
| 480 | PubChem-715-015-62 | −7.3 | 532 | PubChem-155-708-703 | −7.0 | 584 | PubChem-715-006-95 | −6.7 |
| 481 | PubChem-145-998-447 | −7.3 | 533 | PubChem-718-189-85 | −7.0 | 585 | PubChem-146-000-907 | −6.7 |
| 482 | PubChem-141-529-976 | −7.3 | 534 | PubChem-141-755-473 | −7.0 | 586 | PubChem-141-529-335 | −6.7 |
| 483 | PubChem-426-459-43 | −7.3 | 535 | PubChem-145-984-833 | −7.0 | 587 | PubChem-141-529-339 | −6.7 |
| 484 | PubChem-137-647-039 | −7.3 | 536 | PubChem-671-490-70 | −7.0 | 588 | PubChem-141-529-343 | −6.7 |
| 485 | PubChem-426-461-90 | −7.3 | 537 | PubChem-684-285-84 | −7.0 | 589 | PubChem-141-529-344 | −6.7 |
| 486 | PubChem-155-558-088 | −7.3 | 538 | PubChem-898-050-90 | −7.0 | 590 | PubChem-141-529-345 | −6.7 |
| 487 | PubChem-118-726-619 | −7.3 | 539 | PubChem-141-732-115 | −7.0 | 591 | PubChem-122-552-403 | −6.7 |
| 488 | PubChem-122-552-407 | −7.3 | 540 | PubChem-153-947-708 | −7.0 | 592 | PubChem-696-732-41 | −6.7 |
| 489 | PubChem-122-552-408 | −7.3 | 541 | PubChem-715-164-18 | −7.0 | 593 | PubChem-141-529-337 | −6.7 |
| 490 | PubChem-155-531-576 | −7.3 | 542 | PubChem-898-050-99 | −7.0 | 594 | PubChem-141-529-338 | −6.7 |
| 491 | PubChem-715-015-59 | −7.3 | 543 | PubChem-898-051-01 | −7.0 | 595 | PubChem-715-010-46 | −6.7 |
| 492 | PubChem-714-589-83 | −7.3 | 544 | PubChem-141-732-112 | −7.0 | 596 | PubChem-156-703-656 | −6.7 |
| 493 | PubChem-137-644-845 | −7.2 | 545 | PubChem-141-755-476 | −7.0 | 597 | PubChem-426-461-88 | −6.7 |
| 494 | PubChem-145-982-963 | −7.2 | 546 | PubChem-155-512-226 | −7.0 | 598 | PubChem-426-461-87 | −6.6 |
| 495 | PubChem-141-755-497 | −7.2 | 547 | PubChem-145-985-088 | −7.0 | 599 | PubChem-714-517-92 | −6.6 |
| 496 | PubChem-155-547-523 | −7.2 | 548 | PubChem-156-703-635 | −6.9 | 600 | PubChem-141-529-970 | −6.6 |
| 497 | PubChem-134-366-638 | −7.2 | 549 | PubChem-156-703-645 | −6.9 | 601 | PubChem-715-010-45 | −6.6 |
| 498 | PubChem-141-732-082 | −7.2 | 550 | PubChem-426-459-44 | −6.9 | 602 | PubChem-898-019-18 | −6.6 |
| 499 | PubChem-141-529-984 | −7.2 | 551 | PubChem-155-539-470 | −6.9 | 603 | PubChem-718-191-24 | −6.6 |
| 500 | PubChem-145-982-597 | −7.2 | 552 | PubChem-739-472-00 | −6.9 | 604 | PubChem-715-005-40 | −6.6 |
| 501 | PubChem-153-947-711 | −7.2 | 553 | PubChem-714-735-76 | −6.9 | 605 | PubChem-141-529-959 | −6.6 |
| 502 | PubChem-715-015-61 | −7.2 | 554 | PubChem-141-529-342 | −6.9 | 606 | PubChem-898-020-72 | −6.6 |
| 503 | PubChem-715-164-15 | −7.2 | 555 | PubChem-129-905-096 | −6.9 | 607 | PubChem-248-300-96 | −6.6 |
| 504 | PubChem-141-529-973 | −7.2 | 556 | PubChem-134-372-765 | −6.9 | 608 | PubChem-872-870-05 | −6.5 |
| 505 | PubChem-141-532-650 | −7.2 | 557 | PubChem-141-732-105 | −6.9 | 609 | PubChem-145-983-350 | −6.5 |
| 506 | PubChem-156-703-654 | −7.2 | 558 | PubChem-141-529-967 | −6.9 | 610 | PubChem-118-726-634 | −6.5 |
| 507 | PubChem-715-164-14 | −7.2 | 559 | PubChem-144-382-790 | −6.9 | 611 | PubChem-158-991-255 | −6.5 |
| 508 | PubChem-727-005-20 | −7.2 | 560 | PubChem-156-703-652 | −6.9 | 612 | PubChem-714-625-07 | −6.5 |
| 509 | PubChem-141-529-977 | −7.2 | 561 | PubChem-141-732-085 | −6.9 | 613 | PubChem-898-021-25 | −6.5 |
| 510 | PubChem-155-523-945 | −7.2 | 562 | PubChem-155-537-737 | −6.9 | 614 | PubChem-898-021-49 | −6.5 |
| 511 | PubChem-141-532-636 | −7.2 | 563 | PubChem-155-549-287 | −6.9 | 615 | PubChem-155-535-096 | −6.5 |
| 512 | PubChem-898-050-93 | −7.2 | 564 | PubChem-118-726-631 | −6.8 | 616 | PubChem-155-540-727 | −6.5 |
| 513 | PubChem-155-541-020 | −7.1 | 565 | PubChem-118-726-636 | −6.8 | 617 | PubChem-145-982-671 | −6.5 |
| 514 | PubChem-727-004-29 | −7.1 | 566 | PubChem-141-732-097 | −6.8 | 618 | PubChem-715-005-39 | −6.5 |
| 515 | PubChem-145-983-840 | −7.1 | 567 | PubChem-898-050-94 | −6.8 | 619 | PubChem-135-203-618 | −6.4 |
| 516 | PubChem-727-001-54 | −7.1 | 568 | PubChem-141-732-086 | −6.8 | 620 | PubChem-898-051-09 | −6.4 |
| 517 | PubChem-156-703-647 | −7.1 | 569 | PubChem-156-703-650 | −6.8 | 621 | PubChem-141-755-490 | −6.4 |
| 518 | PubChem-146-000-696 | −7.1 | 570 | PubChem-141-529-972 | −6.8 | 622 | PubChem-718-188-45 | −6.4 |
| 519 | PubChem-122-552-415 | −7.1 | 571 | PubChem-145-984-719 | −6.8 | 623 | PubChem-122-552-411 | −6.4 |

## S2 Table. Continued.

| No. | PubChem Code | Covalent Docking Score (kcal/mol) | No. | PubChem Code | Covalent Docking Score (kcal/mol) | No. | PubChem Code | Covalent Docking Score (kcal/mol) |
| --- | --- | --- | --- | --- | --- | --- | --- | --- |
| 624 | PubChem-898-020-57 | −6.4 | 668 | PubChem-145-998-467 | −6.0 | 712 | PubChem-718-188-48 | −5.2 |
| 625 | PubChem-715-008-64 | −6.3 | 669 | PubChem-872-869-78 | −6.0 | 713 | PubChem-727-001-53 | −5.2 |
| 626 | PubChem-156-703-648 | −6.3 | 670 | PubChem-674-263-84 | −5.9 | 714 | PubChem-718-189-84 | −5.2 |
| 627 | PubChem-718-187-08 | −6.3 | 671 | PubChem-739-471-96 | −5.9 | 715 | PubChem-129-100-79 | −5.2 |
| 628 | PubChem-122-552-412 | −6.3 | 672 | PubChem-141-529-336 | −5.9 | 716 | PubChem-718-188-49 | −4.9 |
| 629 | PubChem-156-703-638 | −6.3 | 673 | PubChem-141-529-346 | −5.9 | 717 | PubChem-145-998-619 | −4.9 |
| 630 | PubChem-898-020-11 | −6.3 | 674 | PubChem-898-051-02 | −5.9 | 718 | PubChem-727-003-38 | −4.9 |
| 631 | PubChem-739-472-02 | −6.3 | 675 | PubChem-898-020-26 | −5.9 | 719 | PubChem-147-038-106 | −4.9 |
| 632 | PubChem-118-566-797 | −6.3 | 676 | PubChem-727-005-23 | −5.9 | 720 | PubChem-148-608-135 | −4.9 |
| 633 | PubChem-157-078-851 | −6.3 | 677 | PubChem-134-372-780 | −5.9 | 721 | PubChem-144-553-323 | −4.8 |
| 634 | PubChem-426-459-42 | −6.3 | 678 | PubChem-727-003-37 | −5.8 | 722 | PubChem-714-517-91 | −4.8 |
| 635 | PubChem-714-625-08 | −6.2 | 679 | PubChem-898-021-12 | −5.8 | 723 | PubChem-684-302-18 | −4.8 |
| 636 | PubChem-898-063-18 | −6.2 | 680 | PubChem-134-372-787 | −5.8 | 724 | PubChem-141-446-808 | −4.8 |
| 637 | PubChem-137-643-776 | −6.2 | 681 | PubChem-739-472-04 | −5.8 | 725 | PubChem-727-002-41 | −4.7 |
| 638 | PubChem-714-625-05 | −6.2 | 682 | PubChem-718-187-05 | −5.8 | 726 | PubChem-718-188-47 | −4.7 |
| 639 | PubChem-714-571-13 | −6.2 | 683 | PubChem-146-000-706 | −5.8 | 727 | PubChem-134-714-860 | −4.6 |
| 640 | PubChem-898-020-49 | −6.2 | 684 | PubChem-146-001-006 | −5.7 | 728 | PubChem-146-000-516 | −4.6 |
| 641 | PubChem-898-063-17 | −6.2 | 685 | PubChem-715-010-47 | −5.7 | 729 | PubChem-146-000-917 | −4.6 |
| 642 | PubChem-141-529-340 | −6.2 | 686 | PubChem-145-998-345 | −5.7 | 730 | PubChem-134-714-869 | −4.6 |
| 643 | PubChem-145-985-482 | −6.2 | 687 | PubChem-715-010-48 | −5.7 | 731 | PubChem-545-757-87 | −4.6 |
| 644 | PubChem-714-607-80 | −6.2 | 688 | PubChem-898-063-19 | −5.7 | 732 | PubChem-718-188-50 | −4.5 |
| 645 | PubChem-671-491-02 | −6.2 | 689 | PubChem-161-836-665 | −5.7 | 733 | PubChem-157-182-637 | −4.5 |
| 646 | PubChem-718-187-07 | −6.2 | 690 | PubChem-727-005-22 | −5.7 | 734 | PubChem-718-188-51 | −4.5 |
| 647 | PubChem-153-326-277 | −6.2 | 691 | PubChem-141-538-938 | −5.6 | 735 | PubChem-141-529-341 | −4.5 |
| 648 | PubChem-145-998-448 | −6.1 | 692 | PubChem-156-703-643 | −5.6 | 736 | PubChem-144-404-689 | −4.5 |
| 649 | PubChem-727-005-24 | −6.1 | 693 | PubChem-718-189-81 | −5.6 | 737 | PubChem-135-203-612 | −4.5 |
| 650 | PubChem-145-998-344 | −6.1 | 694 | PubChem-141-529-348 | −5.6 | 738 | PubChem-145-682-010 | −4.5 |
| 651 | PubChem-122-552-404 | −6.1 | 695 | PubChem-146-000-918 | −5.6 | 739 | PubChem-141-538-939 | −4.4 |
| 652 | PubChem-898-018-42 | −6.1 | 696 | PubChem-248-299-25 | −5.6 | 740 | PubChem-141-443-663 | −4.4 |
| 653 | PubChem-149-839-401 | −6.1 | 697 | PubChem-718-189-80 | −5.5 | 741 | PubChem-545-757-22 | −4.4 |
| 654 | PubChem-898-027-46 | −6.1 | 698 | PubChem-248-300-95 | −5.5 | 742 | PubChem-680-133-54 | −4.3 |
| 655 | PubChem-718-187-06 | −6.1 | 699 | PubChem-727-006-05 | −5.5 | 743 | PubChem-718-188-46 | −4.3 |
| 656 | PubChem-898-020-24 | −6.1 | 700 | PubChem-718-189-82 | −5.5 | 744 | PubChem-150-336-399 | −4.2 |
| 657 | PubChem-155-542-912 | −6.1 | 701 | PubChem-718-187-09 | −5.4 | 745 | PubChem-715-015-63 | −4.1 |
| 658 | PubChem-155-557-437 | −6.1 | 702 | PubChem-153-947-705 | −5.4 | 746 | PubChem-141-446-810 | −4.1 |
| 659 | PubChem-118-726-635 | −6.0 | 703 | PubChem-149-337-969 | −5.4 | 747 | PubChem-153-947-701 | −4.0 |
| 660 | PubChem-727-006-03 | −6.0 | 704 | PubChem-714-499-68 | −5.4 | 748 | PubChem-141-529-347 | −3.9 |
| 661 | PubChem-872-870-29 | −6.0 | 705 | PubChem-671-490-93 | −5.3 | 749 | PubChem-696-729-55 | −3.8 |
| 662 | PubChem-898-020-51 | −6.0 | 706 | PubChem-141-446-806 | −5.3 | 750 | PubChem-153-920-774 | −3.7 |
| 663 | PubChem-134-372-734 | −6.0 | 707 | PubChem-569-267-31 | −5.3 | 751 | PubChem-158-785-570 | −1.4 |
| 664 | PubChem-155-708-710 | −6.0 | 708 | PubChem-674-276-50 | −5.3 | 752 | PubChem-159-503-497 | −0.8 |
| 665 | PubChem-134-372-766 | −6.0 | 709 | PubChem-397-422-54 | −5.2 | 753 | PubChem-141-755-471 | −0.7 |
| 666 | PubChem-715-010-44 | −6.0 | 710 | PubChem-153-326-282 | −5.2 | 754 | PubChem-155-708-708 | −0.5 |
| 667 | PubChem-153-947-704 | −6.0 | 711 | PubChem-160-236-858 | −5.2 |  |  |  |
