# Supplementary material for: Benzothiazinone analogs as Anti-Mycobacterium tuberculosis DprE1 irreversible inhibitors: Covalent docking, validation, and molecular dynamics simulations
Source: PLoS One. 2024 Nov 25;19(11):e0314422. doi: 10.1371/journal.pone.0314422 (PMC11588222; doi:10.1371/journal.pone.0314422)
Supplement: S3 Table — (DOCX) [file pone.0314422.s005.docx]

### S3 Table. Calculated fast and expensive covalent docking scores (in kcal/mol) for PBTZ169 and the top 349 potent BTZ analogs against DprE1 active site ^a^.

| No. | PubChem Code | Covalent Docking Score (kcal/mol) | | No. | PubChem Code | Covalent Docking Score (kcal/mol) | |
| --- | --- | --- | --- | --- | --- | --- | --- |
|  |  | Fast | Expensive |  |  | Fast | Expensive |
|  | PBTZ169 | −7.8 | −7.8 | 50 | PubChem-156-636-188 | −9.6 | −9.6 |
| 1 | PubChem-155-924-621 | −15.0 | −15.7 | 51 | PubChem-141-531-232 | −9.4 | −9.6 |
| 2 | PubChem-127-032-794 | −14.3 | −14.7 | 52 | PubChem-146-000-473 | −9.5 | −9.5 |
| 3 | PubChem-127-032-793 | −13.1 | −13.6 | 53 | PubChem-156-636-187 | −9.5 | −9.5 |
| 4 | PubChem-155-923-972 | −12.8 | −13.3 | 54 | PubChem-155-538-834 | −9.5 | −9.5 |
| 5 | PubChem-155-925-252 | −13.4 | −12.7 | 55 | PubChem-894-502-31 | −9.5 | −9.5 |
| 6 | PubChem-155-923-971 | −13.7 | −12.4 | 56 | PubChem-156-636-202 | −9.5 | −9.5 |
| 7 | PubChem-127-031-914 | −11.5 | −12.0 | 57 | PubChem-156-636-173 | −9.5 | −9.5 |
| 8 | PubChem-127-032-792 | −11.6 | −12.3 | 58 | PubChem-141-532-698 | −9.4 | −9.5 |
| 9 | PubChem-155-925-517 | −14.1 | −12.1 | 59 | PubChem-156-636-197 | −9.4 | −9.5 |
| 10 | PubChem-155-535-932 | −10.5 | −11.8 | 60 | PubChem-156-636-179 | −9.4 | −9.5 |
| 11 | PubChem-127-032-795 | −11.0 | −11.6 | 61 | PubChem-153-532-206 | −9.4 | −9.5 |
| 12 | PubChem-127-031-912 | −10.3 | −11.0 | 62 | PubChem-156-636-182 | −9.4 | −9.5 |
| 13 | PubChem-156-636-194 | −10.9 | −10.9 | 63 | PubChem-727-002-40 | −9.0 | −9.4 |
| 14 | PubChem-156-636-196 | −10.9 | −10.9 | 64 | PubChem-727-006-04 | −9.2 | −9.4 |
| 15 | PubChem-127-033-931 | −10.6 | −10.8 | 65 | PubChem-156-636-195 | −9.4 | −9.4 |
| 16 | PubChem-156-636-209 | −10.4 | −10.5 | 66 | PubChem-156-636-206 | −9.3 | −9.4 |
| 17 | PubChem-156-636-180 | −10.4 | −10.5 | 67 | PubChem-141-532-652 | −9.3 | −9.4 |
| 18 | PubChem-127-033-930 | −9.8 | −10.5 | 68 | PubChem-156-636-201 | −9.3 | −9.4 |
| 19 | PubChem-156-636-199 | −10.5 | −10.5 | 69 | PubChem-141-755-477 | −8.3 | −9.3 |
| 20 | PubChem-141-755-487 | −10.2 | −10.4 | 70 | PubChem-145-682-002 | −9.3 | −9.3 |
| 21 | PubChem-156-636-207 | −9.9 | −10.3 | 71 | PubChem-156-636-198 | −9.3 | −9.3 |
| 22 | PubChem-141-531-255 | −10.0 | −10.2 | 72 | PubChem-141-732-101 | −9.2 | −9.3 |
| 23 | PubChem-137-652-159 | −10.2 | −10.2 | 73 | PubChem-156-636-204 | −9.3 | −9.3 |
| 24 | PubChem-156-636-192 | −10.2 | −10.2 | 74 | PubChem-155-525-812 | −9.3 | −9.3 |
| 25 | PubChem-156-636-183 | −10.0 | −10.2 | 75 | PubChem-145-999-200 | −9.2 | −9.3 |
| 26 | PubChem-156-636-213 | −10.2 | −10.2 | 76 | PubChem-141-532-700 | −9.2 | −9.2 |
| 27 | PubChem-156-636-190 | −10.0 | −10.0 | 77 | PubChem-156-636-208 | −9.2 | −9.2 |
| 28 | PubChem-155-558-953 | −9.9 | −10.0 | 78 | PubChem-156-636-200 | −9.1 | −9.2 |
| 29 | PubChem-898-051-07 | −9.8 | −9.9 | 79 | PubChem-156-636-203 | −9.1 | −9.2 |
| 30 | PubChem-156-636-186 | −9.9 | −9.9 | 80 | PubChem-155-564-959 | −9.1 | −9.2 |
| 31 | PubChem-156-636-189 | −9.9 | −9.9 | 81 | PubChem-155-550-726 | −9.2 | −9.2 |
| 32 | PubChem-156-636-185 | −9.7 | −9.9 | 82 | PubChem-141-529-963 | −9.1 | −9.2 |
| 33 | PubChem-141-531-266 | −9.8 | −9.9 | 83 | PubChem-156-636-178 | −9.1 | −9.2 |
| 34 | PubChem-696-725-86 | −9.7 | −9.8 | 84 | PubChem-898-050-97 | −8.8 | −9.2 |
| 35 | PubChem-141-531-258 | −9.8 | −9.8 | 85 | PubChem-141-532-689 | −9.1 | −9.1 |
| 36 | PubChem-156-636-205 | −9.8 | −9.8 | 86 | PubChem-145-999-962 | −9.0 | −9.1 |
| 37 | PubChem-156-636-177 | −9.8 | −9.8 | 87 | PubChem-118-726-618 | −9.1 | −9.1 |
| 38 | PubChem-155-563-064 | −9.4 | −9.8 | 88 | PubChem-141-532-678 | −9.0 | −9.1 |
| 39 | PubChem-156-636-181 | −9.8 | −9.8 | 89 | PubChem-146-000-866 | −9.0 | −9.1 |
| 40 | PubChem-141-532-706 | −9.8 | −9.8 | 90 | PubChem-137-649-378 | −9.0 | −9.1 |
| 41 | PubChem-156-636-191 | −9.8 | −9.8 | 91 | PubChem-898-051-06 | −8.8 | −9.0 |
| 42 | PubChem-141-732-116 | −9.7 | −9.8 | 92 | PubChem-155-514-370 | −8.5 | −9.0 |
| 43 | PubChem-156-636-193 | −9.7 | −9.8 | 93 | PubChem-155-522-525 | −9.0 | −9.0 |
| 44 | PubChem-155-550-499 | −9.6 | −9.7 | 94 | PubChem-141-532-660 | −9.0 | −9.0 |
| 45 | PubChem-156-636-211 | −9.7 | −9.7 | 95 | PubChem-155-547-100 | −8.9 | −8.9 |
| 46 | PubChem-141-755-499 | −9.4 | −9.7 | 96 | PubChem-145-999-092 | −8.9 | −8.9 |
| 47 | PubChem-156-636-212 | −9.6 | −9.7 | 97 | PubChem-141-732-109 | −8.8 | −8.9 |
| 48 | PubChem-156-636-176 | −9.6 | −9.6 | 98 | PubChem-898-018-27 | −8.8 | −8.9 |
| 49 | PubChem-137-637-318 | −9.3 | −9.6 | 99 | PubChem-141-755-482 | −8.9 | −8.9 |

### S3 Table. *Continued*.

| No. | PubChem Code | Covalent Docking Score (kcal/mol) | | No. | PubChem Code | Covalent Docking Score (kcal/mol) | |
| --- | --- | --- | --- | --- | --- | --- | --- |
|  |  | Fast | Expensive |  |  | Fast | Expensive |
| 100 | PubChem-141-532-692 | −8.9 | −8.9 | 151 | PubChem-137-644-321 | −8.6 | −8.6 |
| 101 | PubChem-122-552-414 | −8.8 | −8.9 | 152 | PubChem-161-854-875 | −8.5 | −8.5 |
| 102 | PubChem-156-636-184 | −9.0 | −8.9 | 153 | PubChem-145-998-585 | −8.3 | −8.5 |
| 103 | PubChem-573-878-81 | −8.9 | −8.9 | 154 | PubChem-696-761-86 | −8.5 | −8.5 |
| 104 | PubChem-141-531-241 | −8.8 | −8.9 | 155 | PubChem-141-532-704 | −8.5 | −8.5 |
| 105 | PubChem-155-540-811 | −8.9 | −8.9 | 156 | PubChem-898-051-08 | −8.4 | −8.5 |
| 106 | PubChem-156-703-637 | −8.8 | −8.9 | 157 | PubChem-875-023-69 | −8.5 | −8.5 |
| 107 | PubChem-727-004-32 | −8.8 | −8.9 | 158 | PubChem-573-878-80 | −8.5 | −8.5 |
| 108 | PubChem-141-532-693 | −8.5 | −8.9 | 159 | PubChem-118-726-621 | −8.5 | −8.5 |
| 109 | PubChem-137-632-150 | −8.8 | −8.8 | 160 | PubChem-898-050-91 | −8.3 | −8.5 |
| 110 | PubChem-141-755-480 | −8.8 | −8.8 | 161 | PubChem-161-180-922 | −8.3 | −8.5 |
| 111 | PubChem-155-568-069 | −8.8 | −8.8 | 162 | PubChem-146-000-368 | −8.4 | −8.5 |
| 112 | PubChem-145-999-223 | −8.7 | −8.8 | 163 | PubChem-137-653-833 | −8.5 | −8.5 |
| 113 | PubChem-141-755-472 | −8.8 | −8.8 | 164 | PubChem-156-776-528 | −8.5 | −8.5 |
| 114 | PubChem-727-005-25 | −8.8 | −8.8 | 165 | PubChem-141-532-696 | −8.4 | −8.5 |
| 115 | PubChem-141-529-981 | −8.7 | −8.8 | 166 | PubChem-141-532-651 | −8.4 | −8.5 |
| 116 | PubChem-137-634-174 | −8.7 | −8.8 | 167 | PubChem-141-531-248 | −8.4 | −8.5 |
| 117 | PubChem-118-726-617 | −8.8 | −8.8 | 168 | PubChem-146-000-697 | −8.3 | −8.5 |
| 118 | PubChem-898-020-15 | −8.5 | −8.8 | 169 | PubChem-141-755-496 | −8.4 | −8.5 |
| 119 | PubChem-714-607-79 | −8.7 | −8.8 | 170 | PubChem-118-726-620 | −8.4 | −8.5 |
| 120 | PubChem-141-532-707 | −8.7 | −8.8 | 171 | PubChem-145-999-091 | −8.4 | −8.4 |
| 121 | PubChem-141-532-685 | −8.7 | −8.8 | 172 | PubChem-155-538-457 | −8.4 | −8.4 |
| 122 | PubChem-156-636-174 | −8.7 | −8.7 | 173 | PubChem-146-000-167 | −8.4 | −8.4 |
| 123 | PubChem-141-755-494 | −8.2 | −8.7 | 174 | PubChem-141-532-684 | −8.4 | −8.4 |
| 124 | PubChem-141-755-475 | −8.6 | −8.7 | 175 | PubChem-118-712-952 | −8.0 | −8.4 |
| 125 | PubChem-141-532-705 | −8.7 | −8.7 | 176 | PubChem-141-532-638 | −8.4 | −8.4 |
| 126 | PubChem-127-034-462 | −9.0 | −8.7 | 177 | PubChem-141-532-701 | −8.3 | −8.4 |
| 127 | PubChem-155-562-434 | −8.6 | −8.7 | 178 | PubChem-141-532-643 | −8.3 | −8.4 |
| 128 | PubChem-145-999-339 | −8.6 | −8.7 | 179 | PubChem-727-004-31 | −8.4 | −8.4 |
| 129 | PubChem-141-532-688 | −8.7 | −8.7 | 180 | PubChem-141-755-474 | −8.4 | −8.4 |
| 130 | PubChem-141-532-649 | −8.7 | −8.7 | 181 | PubChem-141-532-694 | −8.4 | −8.4 |
| 131 | PubChem-129-904-732 | −8.6 | −8.7 | 182 | PubChem-715-164-17 | −8.1 | −8.4 |
| 132 | PubChem-146-000-472 | −8.4 | −8.7 | 183 | PubChem-141-532-653 | −8.4 | −8.4 |
| 133 | PubChem-141-529-965 | −8.6 | −8.7 | 184 | PubChem-137-651-027 | −8.4 | −8.4 |
| 134 | PubChem-573-878-82 | −8.5 | −8.7 | 185 | PubChem-155-435-068 | −8.4 | −8.4 |
| 135 | PubChem-141-532-640 | −8.6 | −8.7 | 186 | PubChem-141-532-639 | −8.4 | −8.4 |
| 136 | PubChem-146-000-962 | −8.6 | −8.6 | 187 | PubChem-898-050-95 | −8.3 | −8.4 |
| 137 | PubChem-141-532-680 | −8.6 | −8.6 | 188 | PubChem-155-568-053 | −8.4 | −8.4 |
| 138 | PubChem-145-984-191 | −8.6 | −8.6 | 189 | PubChem-155-512-871 | −8.4 | −8.4 |
| 139 | PubChem-141-532-644 | −8.6 | −8.6 | 190 | PubChem-573-881-14 | −8.2 | −8.4 |
| 140 | PubChem-573-881-13 | −8.5 | −8.6 | 191 | PubChem-141-531-247 | −8.3 | −8.4 |
| 141 | PubChem-142-758-240 | −8.5 | −8.6 | 192 | PubChem-141-531-236 | −8.3 | −8.4 |
| 142 | PubChem-141-532-690 | −8.6 | −8.6 | 193 | PubChem-137-655-686 | −7.8 | −8.4 |
| 143 | PubChem-137-656-146 | −8.6 | −8.6 | 194 | PubChem-141-532-647 | −8.3 | −8.3 |
| 144 | PubChem-155-541-557 | −8.6 | −8.6 | 195 | PubChem-141-531-230 | −8.3 | −8.3 |
| 145 | PubChem-145-998-704 | −7.9 | −8.6 | 196 | PubChem-898-022-46 | −7.9 | −8.3 |
| 146 | PubChem-141-532-646 | −8.6 | −8.6 | 197 | PubChem-145-998-586 | −8.3 | −8.3 |
| 147 | PubChem-156-636-210 | −8.6 | −8.6 | 198 | PubChem-141-532-697 | −8.2 | −8.3 |
| 148 | PubChem-141-532-686 | −8.6 | −8.6 | 199 | PubChem-141-532-645 | −8.3 | −8.3 |
| 149 | PubChem-573-881-12 | −7.8 | −8.6 | 200 | PubChem-141-531-251 | −8.3 | −8.3 |
| 150 | PubChem-727-002-39 | −8.5 | −8.6 | 201 | PubChem-137-661-096 | −8.3 | −8.3 |

### S3 Table. *Continued*.

| No. | PubChem Code | Covalent Docking Score (kcal/mol) | | No. | PubChem Code | Covalent Docking Score (kcal/mol) | |
| --- | --- | --- | --- | --- | --- | --- | --- |
|  |  | Fast | Expensive |  |  | Fast | Expensive |
| 202 | PubChem-674-271-15 | −8.3 | −8.3 | 253 | PubChem-141-531-244 | −8.1 | −8.1 |
| 203 | PubChem-141-531-262 | −8.3 | −8.3 | 254 | PubChem-141-531-240 | −8.1 | −8.1 |
| 204 | PubChem-118-726-627 | −8.3 | −8.3 | 255 | PubChem-155-533-696 | −8.0 | −8.1 |
| 205 | PubChem-145-999-885 | −8.3 | −8.3 | 256 | PubChem-155-533-314 | −8.1 | −8.1 |
| 206 | PubChem-141-531-235 | −8.3 | −8.3 | 257 | PubChem-141-755-484 | −8.1 | −8.1 |
| 207 | PubChem-137-657-258 | −8.2 | −8.3 | 258 | PubChem-141-532-679 | −8.0 | −8.1 |
| 208 | PubChem-146-001-133 | −8.3 | −8.3 | 259 | PubChem-141-532-677 | −8.1 | −8.1 |
| 209 | PubChem-141-532-662 | −8.2 | −8.3 | 260 | PubChem-141-732-111 | −7.9 | −8.1 |
| 210 | PubChem-141-755-485 | −8.3 | −8.3 | 261 | PubChem-141-532-665 | −8.1 | −8.1 |
| 211 | PubChem-134-366-636 | −8.2 | −8.3 | 262 | PubChem-145-984-054 | −8.1 | −8.1 |
| 212 | PubChem-248-300-94 | −8.3 | −8.3 | 263 | PubChem-122-552-410 | −8.0 | −8.1 |
| 213 | PubChem-146-000-011 | −8.2 | −8.3 | 264 | PubChem-122-552-409 | −8.0 | −8.1 |
| 214 | PubChem-144-404-685 | −8.1 | −8.3 | 265 | PubChem-141-532-681 | −8.1 | −8.1 |
| 215 | PubChem-141-732-104 | −8.2 | −8.3 | 266 | PubChem-141-532-676 | −8.1 | −8.1 |
| 216 | PubChem-141-531-242 | −8.2 | −8.3 | 267 | PubChem-141-532-664 | −8.0 | −8.1 |
| 217 | PubChem-739-471-95 | −8.3 | −8.3 | 268 | PubChem-898-051-04 | −8.1 | −8.1 |
| 218 | PubChem-727-003-36 | −8.1 | −8.3 | 269 | PubChem-155-551-745 | −8.0 | −8.1 |
| 219 | PubChem-573-881-15 | −8.0 | −8.3 | 270 | PubChem-141-732-117 | −7.9 | −8.1 |
| 220 | PubChem-156-703-646 | −8.1 | −8.3 | 271 | PubChem-715-013-74 | −7.8 | −8.1 |
| 221 | PubChem-141-732-095 | −8.2 | −8.3 | 272 | PubChem-156-776-527 | −8.0 | −8.1 |
| 222 | PubChem-141-531-253 | −8.2 | −8.3 | 273 | PubChem-137-639-860 | −7.8 | −8.1 |
| 223 | PubChem-122-552-401 | −8.0 | −8.3 | 274 | PubChem-723-750-17 | −8.0 | −8.1 |
| 224 | PubChem-122-552-400 | −8.0 | −8.3 | 275 | PubChem-144-404-686 | −8.0 | −8.1 |
| 225 | PubChem-727-003-35 | −8.2 | −8.2 | 276 | PubChem-141-732-084 | −8.1 | −8.1 |
| 226 | PubChem-156-703-636 | −8.0 | −8.2 | 277 | PubChem-141-532-654 | −8.0 | −8.1 |
| 227 | PubChem-118-726-632 | −8.2 | −8.2 | 278 | PubChem-141-531-239 | −8.0 | −8.1 |
| 228 | PubChem-727-004-33 | −8.2 | −8.2 | 279 | PubChem-141-531-237 | −8.0 | −8.1 |
| 229 | PubChem-696-761-85 | −8.2 | −8.2 | 280 | PubChem-118-726-628 | −8.0 | −8.1 |
| 230 | PubChem-122-552-420 | −8.2 | −8.2 | 281 | PubChem-146-000-203 | −8.0 | −8.0 |
| 231 | PubChem-898-020-56 | −8.1 | −8.2 | 282 | PubChem-141-732-083 | −8.0 | −8.0 |
| 232 | PubChem-158-904-351 | −8.2 | −8.2 | 283 | PubChem-141-531-260 | −8.0 | −8.0 |
| 233 | PubChem-156-703-653 | −8.2 | −8.2 | 284 | PubChem-137-641-460 | −8.0 | −8.0 |
| 234 | PubChem-141-755-489 | −7.9 | −8.2 | 285 | PubChem-141-732-114 | −8.0 | −8.0 |
| 235 | PubChem-118-570-041 | −8.2 | −8.2 | 286 | PubChem-141-532-675 | −8.0 | −8.0 |
| 236 | PubChem-146-000-658 | −8.1 | −8.2 | 287 | PubChem-141-532-648 | −8.0 | −8.0 |
| 237 | PubChem-141-531-263 | −8.2 | −8.2 | 288 | PubChem-141-531-252 | −8.0 | −8.0 |
| 238 | PubChem-727-004-34 | −8.2 | −8.2 | 289 | PubChem-156-703-651 | −8.0 | −8.0 |
| 239 | PubChem-156-703-649 | −8.0 | −8.2 | 290 | PubChem-137-646-426 | −8.0 | −8.0 |
| 240 | PubChem-714-607-78 | −8.2 | −8.2 | 291 | PubChem-134-372-729 | −8.0 | −8.0 |
| 241 | PubChem-156-703-641 | −7.8 | −8.2 | 292 | PubChem-141-531-243 | −8.0 | −8.0 |
| 242 | PubChem-141-732-081 | −8.0 | −8.2 | 293 | PubChem-141-755-492 | −8.0 | −8.0 |
| 243 | PubChem-155-557-174 | −8.2 | −8.2 | 294 | PubChem-141-532-673 | −8.0 | −8.0 |
| 244 | PubChem-141-532-667 | −8.1 | −8.2 | 295 | PubChem-118-726-633 | −7.9 | −8.0 |
| 245 | PubChem-141-532-663 | −8.2 | −8.2 | 296 | PubChem-898-050-88 | −8.0 | −8.0 |
| 246 | PubChem-141-532-658 | −8.1 | −8.2 | 297 | PubChem-145-998-587 | −8.5 | −8.0 |
| 247 | PubChem-898-051-03 | −8.5 | −8.2 | 298 | PubChem-141-732-089 | −7.9 | −8.0 |
| 248 | PubChem-137-656-990 | −8.1 | −8.2 | 299 | PubChem-141-732-087 | −7.8 | −8.0 |
| 249 | PubChem-141-732-088 | −8.1 | −8.2 | 300 | PubChem-155-542-479 | −7.9 | −8.0 |
| 250 | PubChem-141-532-708 | −8.1 | −8.2 | 301 | PubChem-134-262-692 | −7.9 | −8.0 |
| 251 | PubChem-727-001-56 | −8.0 | −8.1 | 302 | PubChem-122-552-402 | −7.9 | −8.0 |
| 252 | PubChem-141-532-671 | −8.1 | −8.1 | 303 | PubChem-146-000-168 | −7.9 | −8.0 |

## S3 Table. Continued.

| No. | PubChem Code | Covalent Docking Score (kcal/mol) | | No. | PubChem Code | Covalent Docking Score (kcal/mol) | |
| --- | --- | --- | --- | --- | --- | --- | --- |
|  |  | Fast | Expensive |  |  | Fast | Expensive |
| 304 | PubChem-141-532-655 | −8.0 | −8.0 | 327 | PubChem-141-532-683 | −7.8 | −7.9 |
| 305 | PubChem-141-532-709 | −7.9 | −8.0 | 328 | PubChem-141-531-234 | −7.9 | −7.9 |
| 306 | PubChem-141-732-102 | −7.9 | −7.9 | 329 | PubChem-118-726-626 | −7.9 | −7.9 |
| 307 | PubChem-715-006-96 | −7.9 | −7.9 | 330 | PubChem-674-276-33 | −7.9 | −7.9 |
| 308 | PubChem-141-531-259 | −7.9 | −7.9 | 331 | PubChem-141-532-699 | −7.8 | −7.9 |
| 309 | PubChem-141-531-250 | −7.9 | −7.9 | 332 | PubChem-141-532-691 | −7.8 | −7.9 |
| 310 | PubChem-160-162-190 | −7.9 | −7.9 | 333 | PubChem-141-531-256 | −7.9 | −7.9 |
| 311 | PubChem-142-308-757 | −7.9 | −7.9 | 334 | PubChem-137-657-434 | −7.8 | −7.9 |
| 312 | PubChem-137-633-247 | −7.9 | −7.9 | 335 | PubChem-118-726-622 | −7.8 | −7.9 |
| 313 | PubChem-137-657-350 | −7.8 | −7.9 | 336 | PubChem-141-732-091 | −7.8 | −7.8 |
| 314 | PubChem-145-982-981 | −7.8 | −7.9 | 337 | PubChem-141-732-107 | −7.8 | −7.8 |
| 315 | PubChem-137-659-885 | −7.9 | −7.9 | 338 | PubChem-727-002-38 | −7.8 | −7.8 |
| 316 | PubChem-134-372-749 | −7.9 | −7.9 | 339 | PubChem-141-732-094 | −7.8 | −7.8 |
| 317 | PubChem-145-998-336 | −7.8 | −7.9 | 340 | PubChem-141-532-687 | −7.8 | −7.8 |
| 318 | PubChem-145-985-702 | −7.8 | −7.9 | 341 | PubChem-727-001-55 | −7.8 | −7.8 |
| 319 | PubChem-141-532-682 | −7.8 | −7.9 | 342 | PubChem-141-532-695 | −7.8 | −7.8 |
| 320 | PubChem-141-732-110 | −7.9 | −7.9 | 343 | PubChem-141-529-985 | −7.8 | −7.8 |
| 321 | PubChem-141-532-702 | −7.8 | −7.9 | 344 | PubChem-118-726-629 | −7.8 | −7.8 |
| 322 | PubChem-137-635-021 | −7.8 | −7.9 | 345 | PubChem-739-471-97 | −7.8 | −7.8 |
| 323 | PubChem-898-022-85 | −7.9 | −7.9 | 346 | PubChem-141-531-264 | −7.8 | −7.8 |
| 324 | PubChem-894-502-30 | −7.9 | −7.9 | 347 | PubChem-426-461-86 | −7.8 | −7.8 |
| 325 | PubChem-155-708-694 | −7.8 | −7.9 | 348 | PubChem-248-302-60 | −7.8 | −7.8 |
| 326 | PubChem-155-708-693 | −7.8 | −7.9 | 349 | PubChem-102-004-363 | −7.8 | −7.8 |

^a^Data ranked based on expensive covalent docking scores (in kcal/mol) for BTZ analogs against DprE1 active site.
