# Supplementary material for: Benzothiazinone analogs as Anti-Mycobacterium tuberculosis DprE1 irreversible inhibitors: Covalent docking, validation, and molecular dynamics simulations
Source: PLoS One. 2024 Nov 25;19(11):e0314422. doi: 10.1371/journal.pone.0314422 (PMC11588222; doi:10.1371/journal.pone.0314422)
Supplement: S4 Table — (DOCX) [file pone.0314422.s006.docx]

**S4 Table**. Estimated fast and expensive covalent docking scores and MM-GBSA binding energies (in kcal/mol) over 1 ns MD simulations for PBTZ169 and the top 94 potent BTZ analogs within DprE1 active site ^a^.

| No. | PubChem Code | Covalent Docking Score (kcal/mol) | | MM-GBSA Binding Energy (kcal/mol) |
| --- | --- | --- | --- | --- |
|  |  | Fast | Expensive |  |
|  | PBTZ169 | −7.8 | −7.8 | −37.9 |
| 1 | PubChem-127-031-914 | −11.5 | −12.0 | −73.5 |
| 2 | PubChem-155-924-621 | −15.0 | −15.7 | −68.5 |
| 3 | PubChem-137-637-318 | −9.3 | −9.6 | −64.1 |
| 4 | PubChem-127-032-795 | −11.0 | −11.6 | −64.0 |
| 5 | PubChem-127-032-794 | −14.3 | −14.7 | −63.3 |
| 6 | PubChem-141-529-963 | −9.1 | −9.2 | −61.3 |
| 7 | PubChem-155-925-517 | −14.1 | −12.1 | −61.2 |
| 8 | PubChem-155-923-972 | −12.8 | −13.3 | −60.6 |
| 9 | PubChem-727-006-04 | −9.2 | −9.4 | −58.3 |
| 10 | PubChem-153-532-206 | −9.4 | −9.5 | −57.2 |
| 11 | PubChem-898-051-07 | −9.8 | −9.9 | −57.0 |
| 12 | PubChem-141-755-477 | −8.3 | −9.3 | −56.5 |
| 13 | PubChem-155-563-064 | −9.4 | −9.8 | −55.9 |
| 14 | PubChem-696-725-86 | −9.7 | −9.8 | −55.8 |
| 15 | PubChem-155-923-971 | −13.7 | −12.4 | −55.2 |
| 16 | PubChem-156-636-173 | −9.5 | −9.5 | −54.3 |
| 17 | PubChem-156-636-185 | −9.7 | −9.9 | −54.1 |
| 18 | PubChem-141-531-255 | −10.0 | −10.2 | −53.8 |
| 19 | PubChem-155-925-252 | −13.4 | −12.7 | −53.6 |
| 20 | PubChem-141-755-487 | −10.2 | −10.4 | −53.2 |
| 21 | PubChem-141-532-689 | −9.1 | −9.1 | −52.6 |
| 22 | PubChem-127-032-792 | −11.6 | −12.3 | −52.2 |
| 23 | PubChem-156-636-176 | −9.6 | −9.6 | −51.8 |
| 24 | PubChem-145-999-962 | −9.0 | −9.1 | −51.3 |
| 25 | PubChem-127-033-930 | −9.8 | −10.5 | −50.7 |
| 26 | PubChem-141-532-700 | −9.2 | −9.2 | −50.6 |
| 27 | PubChem-146-000-473 | −9.5 | −9.5 | −49.9 |
| 28 | PubChem-156-636-186 | −9.9 | −9.9 | −49.7 |
| 29 | PubChem-156-636-177 | −9.8 | −9.8 | −49.7 |
| 30 | PubChem-156-636-182 | −9.4 | −9.5 | −49.7 |
| 31 | PubChem-156-636-181 | −9.8 | −9.8 | −49.1 |
| 32 | PubChem-145-999-200 | −9.2 | −9.3 | −49.0 |
| 33 | PubChem-141-732-101 | −9.2 | −9.3 | −49.0 |
| 34 | PubChem-156-636-191 | −9.8 | −9.8 | −48.4 |
| 35 | PubChem-127-032-793 | −13.1 | −13.6 | −48.2 |
| 36 | PubChem-156-636-213 | −10.2 | −10.2 | −48.0 |
| 37 | PubChem-141-532-698 | −9.4 | −9.5 | −48.0 |
| 38 | PubChem-141-755-499 | −9.4 | −9.7 | −47.2 |
| 39 | PubChem-898-050-97 | −8.8 | −9.2 | −47.1 |
| 40 | PubChem-127-031-912 | −10.3 | −11.0 | −46.6 |
| 41 | PubChem-145-682-002 | −9.3 | −9.3 | −46.6 |
| 42 | PubChem-156-636-208 | −9.2 | −9.2 | −46.3 |
| 43 | PubChem-146-000-866 | −9.0 | −9.1 | −46.1 |
| 44 | PubChem-156-636-189 | −9.9 | −9.9 | −46.1 |
| 45 | PubChem-155-525-812 | −9.3 | −9.3 | −46.1 |
| 46 | PubChem-155-538-834 | −9.5 | −9.5 | −46.0 |

**S4 Table**. *Continued***.**

| No. | PubChem Code | Covalent Docking Score (kcal/mol) | | MM-GBSA Binding Energy (kcal/mol) |
| --- | --- | --- | --- | --- |
|  |  | Fast | Expensive |  |
| 47 | PubChem-156-636-179 | −9.4 | −9.5 | −45.5 |
| 48 | PubChem-156-636-197 | −9.4 | −9.5 | −45.5 |
| 49 | PubChem-156-636-206 | −9.3 | −9.4 | −45.2 |
| 50 | PubChem-141-531-266 | −9.8 | −9.9 | −45.1 |
| 51 | PubChem-141-532-706 | −9.8 | −9.8 | −44.9 |
| 52 | PubChem-156-636-178 | −9.1 | −9.2 | −44.6 |
| 53 | PubChem-156-636-195 | −9.4 | −9.4 | −44.6 |
| 54 | PubChem-141-732-116 | −9.7 | −9.8 | −44.5 |
| 55 | PubChem-894-502-31 | −9.5 | −9.5 | −44.2 |
| 56 | PubChem-156-636-180 | −10.4 | −10.5 | −44.1 |
| 57 | PubChem-137-649-378 | −9.0 | −9.1 | −44.0 |
| 58 | PubChem-156-636-196 | −10.9 | −10.9 | −44.0 |
| 59 | PubChem-156-636-202 | −9.5 | −9.5 | −43.8 |
| 60 | PubChem-156-636-209 | −10.4 | −10.5 | −43.6 |
| 61 | PubChem-156-636-200 | −9.1 | −9.2 | −43.5 |
| 62 | PubChem-155-550-499 | −9.6 | −9.7 | −43.4 |
| 63 | PubChem-156-636-199 | −10.5 | −10.5 | −43.3 |
| 64 | PubChem-898-051-06 | −8.8 | −9.0 | −43.1 |
| 65 | PubChem-156-636-183 | −10.0 | −10.2 | −42.7 |
| 66 | PubChem-141-531-232 | −9.4 | −9.6 | −42.7 |
| 67 | PubChem-118-726-618 | −9.1 | −9.1 | −42.5 |
| 68 | PubChem-141-532-652 | −9.3 | −9.4 | −42.2 |
| 69 | PubChem-141-531-258 | −9.8 | −9.8 | −42.0 |
| 70 | PubChem-156-636-207 | −9.9 | −10.3 | −41.8 |
| 71 | PubChem-156-636-194 | −10.9 | −10.9 | −41.7 |
| 72 | PubChem-127-033-931 | −10.6 | −10.8 | −41.7 |
| 73 | PubChem-156-636-198 | −9.3 | −9.3 | −41.2 |
| 74 | PubChem-156-636-190 | −10.0 | −10.0 | −40.8 |
| 75 | PubChem-156-636-193 | −9.7 | −9.8 | −40.6 |
| 76 | PubChem-156-636-205 | −9.8 | −9.8 | −40.2 |
| 77 | PubChem-156-636-204 | −9.3 | −9.3 | −39.8 |
| 78 | PubChem-156-636-188 | −9.6 | −9.6 | −39.6 |
| 79 | PubChem-141-532-660 | −9.0 | −9.0 | −39.1 |
| 80 | PubChem-156-636-203 | −9.1 | −9.2 | −39.0 |
| 81 | PubChem-156-636-192 | −10.2 | −10.2 | −38.9 |
| 82 | PubChem-156-636-211 | −9.7 | −9.7 | −38.5 |
| 83 | PubChem-156-636-187 | −9.5 | −9.5 | −37.4 |
| 84 | PubChem-141-532-678 | −9.0 | −9.1 | −37.3 |
| 85 | PubChem-155-564-959 | −9.1 | −9.2 | −35.2 |
| 86 | PubChem-727-002-40 | −9.0 | −9.4 | −35.0 |
| 87 | PubChem-155-514-370 | −8.5 | −9.0 | −32.9 |
| 88 | PubChem-137-652-159 | −10.2 | −10.2 | −32.7 |
| 89 | PubChem-156-636-201 | −9.3 | −9.4 | −32.5 |
| 90 | PubChem-156-636-212 | −9.6 | −9.7 | −32.5 |
| 91 | PubChem-155-522-525 | −9.0 | −9.0 | −31.4 |
| 92 | PubChem-155-535-932 | −10.5 | −11.8 | −30.0 |
| 93 | PubChem-155-550-726 | −9.2 | −9.2 | −27.9 |
| 94 | PubChem-155-558-953 | −9.9 | −10.0 | −26.1 |

^a^Data ranked based on the MM-GBSA binding energies over the 1 ns MD simulations.
