# Supplementary material for: Benzothiazinone analogs as Anti-Mycobacterium tuberculosis DprE1 irreversible inhibitors: Covalent docking, validation, and molecular dynamics simulations
Source: PLoS One. 2024 Nov 25;19(11):e0314422. doi: 10.1371/journal.pone.0314422 (PMC11588222; doi:10.1371/journal.pone.0314422)
Supplement: S6 Table — (DOCX) [file pone.0314422.s008.docx]

**S6 Table**. Estimated fast and expensive covalent docking scores and MM-GBSA binding energies (in kcal/mol) over 1, 10, and 25 ns MD simulations for PBTZ169 and the top 23 potent BTZ analogs within DprE1 active site ^a^.

| No. | PubChem Code | Covalent Docking Score (kcal/mol) | | MM-GBSA Binding Energy (kcal/mol) | | |
| --- | --- | --- | --- | --- | --- | --- |
|  |  | Fast | Expensive | 1 ns | 10 ns | 25 ns |
|  | PBTZ169 | −7.8 | −7.8 | −37.9 | −41.4 | −42.9 |
| 1 | PubChem-155-924-621 | −15.0 | −15.7 | −68.5 | −69.4 | −73.1 |
| 2 | PubChem-127-031-914 | −11.5 | −12.0 | −73.5 | −71.7 | −65.6 |
| 3 | PubChem-155-923-972 | −12.8 | −13.3 | −60.6 | −61.3 | −62.8 |
| 4 | PubChem-127-032-794 | −14.3 | −14.7 | −63.3 | −67.8 | −59.8 |
| 5 | PubChem-127-032-795 | −11.0 | −11.6 | −64.0 | −65.8 | −59.7 |
| 6 | PubChem-155-925-517 | −14.1 | −12.1 | −61.2 | −63.7 | −59.5 |
| 7 | PubChem-156-636-173 | −9.5 | −9.5 | −54.3 | −57.2 | −59.4 |
| 8 | PubChem-898-051-07 | −9.8 | −9.9 | −57.0 | −58.5 | −58.7 |
| 9 | PubChem-155-925-252 | −13.4 | −12.7 | −53.6 | −56.3 | −57.7 |
| 10 | PubChem-137-637-318 | −9.3 | −9.6 | −64.1 | −62.3 | −56.9 |
| 11 | PubChem-141-755-477 | −8.3 | −9.3 | −56.5 | −56.1 | −54.1 |
| 12 | PubChem-155-923-971 | −13.7 | −12.4 | −55.2 | −58.0 | −53.6 |
| 13 | PubChem-156-636-185 | −9.7 | −9.9 | −54.1 | −54.6 | −53.2 |
| 14 | PubChem-141-531-255 | −10.0 | −10.2 | −53.8 | −52.4 | −53.0 |
| 15 | PubChem-156-636-177 | −9.8 | −9.8 | −49.7 | −51.3 | −52.5 |
| 16 | PubChem-141-755-487 | −10.2 | −10.4 | −53.2 | −51.8 | −51.7 |
| 17 | PubChem-127-033-930 | −9.8 | −10.5 | −50.7 | −51.1 | −51.5 |
| 18 | PubChem-155-563-064 | −9.4 | −9.8 | −55.9 | −52.6 | −50.1 |
| 19 | PubChem-696-725-86 | −9.7 | −9.8 | −55.8 | −53.6 | −50.0 |
| 20 | PubChem-141-529-963 | −9.1 | −9.2 | −61.3 | −57.0 | −49.7 |
| 21 | PubChem-727-006-04 | −9.2 | −9.4 | −58.3 | −54.8 | −49.7 |
| 22 | PubChem-153-532-206 | −9.4 | −9.5 | −57.2 | −55.6 | −49.4 |
| 23 | PubChem-145-999-962 | −9.0 | −9.1 | −51.3 | −52.0 | −48.1 |

^a^Data ranked based on the MM-GBSA binding energies over the 25 ns MD simulations.
